# Highly efficient genome editing via CRISPR–Cas9 in human pluripotent stem cells is achieved by transient BCL-XL overexpression

**DOI:** 10.1093/nar/gky804

**Published:** 2018-09-20

**Authors:** Xiao-Lan Li, Guo-Hua Li, Juan Fu, Ya-Wen Fu, Lu Zhang, Wanqiu Chen, Cameron Arakaki, Jian-Ping Zhang, Wei Wen, Mei Zhao, Weisheng V Chen, Gary D Botimer, David Baylink, Leslie Aranda, Hannah Choi, Rachel Bechar, Prue Talbot, Chang-Kai Sun, Tao Cheng, Xiao-Bing Zhang

**Affiliations:** 1 State Key Laboratory of Experimental Hematology, Tianjin 300020, China; 2 Institute of Hematology and Blood Disease Hospital, Tianjin 300020, China; 3 Liaoning Provincial Key Laboratory of Cerebral Diseases, Institute for Brain Disorders, Dalian Medical University, Dalian 116044, China; 4 Department of Obstetrics and Gynecology, the First Affiliated Hospital of Dalian Medical University, Dalian 116044, China; 5 Department of Medicine, Loma Linda University, Loma Linda, CA 92350, USA; 6 CAMS Key Laboratory of Gene Therapy for Blood Diseases, Tianjin 300020, China; 7 Leveragen, Inc., Cambridge, MA 01605, USA; 8 Department of Orthopaedic Surgery, Loma Linda University, Loma Linda, CA 92350, USA; 9 UCR Stem Cell Center and Core, University of California at Riverside, Riverside, CA 92521, USA; 10 Research & Educational Center for the Control Engineering of Translational Precision Medicine (R-ECCE-TPM), School of Biomedical Engineering, Faculty of Electronic Information and Electrical Engineering, Dalian University of Technology, Dalian 116024, China; 11 State Key Laboratory of Fine Chemicals, Dalian R&D Center for Stem Cell and Tissue Engineering, Dalian University of Technology, Dalian 116024, China; 12 Center for Stem Cell Medicine, Chinese Academy of Medical Sciences, Tianjin 300020, China; 13 Department of Stem Cell & Regenerative Medicine, Peking Union Medical College, Tianjin 300020, China; 14 Collaborative Innovation Center for Cancer Medicine, Tianjin 300020, China

## Abstract

Genome editing of human induced pluripotent stem cells (iPSCs) is instrumental for functional genomics, disease modeling, and regenerative medicine. However, low editing efficiency has hampered the applications of CRISPR–Cas9 technology in creating knockin (KI) or knockout (KO) iPSC lines, which is largely due to massive cell death after electroporation with editing plasmids. Here, we report that the transient delivery of BCL-XL increases iPSC survival by ∼10-fold after plasmid transfection, leading to a 20- to 100-fold increase in homology-directed repair (HDR) KI efficiency and a 5-fold increase in non-homologous end joining (NHEJ) KO efficiency. Treatment with a BCL inhibitor ABT-263 further improves HDR efficiency by 70% and KO efficiency by 40%. The increased genome editing efficiency is attributed to higher expressions of Cas9 and sgRNA in surviving cells after electroporation. HDR or NHEJ efficiency reaches 95% with dual editing followed by selection of cells with HDR insertion of a selective gene. Moreover, KO efficiency of 100% can be achieved in a bulk population of cells with biallelic HDR KO followed by double selection, abrogating the necessity for single cell cloning. Taken together, these simple yet highly efficient editing strategies provide useful tools for applications ranging from manipulating human iPSC genomes to creating gene-modified animal models.

## INTRODUCTION

Human embryonic stem cells (ESCs) provide a sufficient cell source for regenerative medicine due to their unlimited self-renewal capacity (1). The discovery of patient-specific induced pluripotent stem cells (iPSCs) solved both the immunogenic problem associated with the transplantation of allogeneic cells as well as ethical concerns (2,3). Recently, considerable progress has been made to generate iPSCs from readily available cell sources like peripheral blood and the use of non-integrating vectors that express reprogramming factors (4). However, to realize the full potential of iPSCs in regenerative medicine and disease modeling, disease-causing genes often need to be corrected or modified prior to conducting therapy.

Gene targeting in mouse ESCs was achieved decades ago, albeit at extremely low efficiencies (5). Further studies led to a realization that the early success had unwittingly exploited the cell’s intrinsic repair mechanism after spontaneous genomic DNA breaks (6). However, naturally occurring double-stranded DNA breaks (DSBs) surrounding a target locus are extremely rare, often limiting the targeting efficiency to levels to one in a million, even with the use of homology arms (HA) extending 10 kb pairs (7). To enhance gene targeting, tremendous effort over the past two decades has focused on creating DSBs at certain loci by targetable endonucleases. While the development of engineered endonucleases, like zinc-finger nucleases or transcription activator-like effector nucleases, have generated excitement, their limitations in design or cloning have rendered them impractical for routine laboratory use (8,9). The latest generation of RNA-guided endonuclease, or CRISPR–Cas9, has been widely used due to its simplicity in vector design and robustness in performance (10–12). CRISPR–Cas9 is an adaptive immune system that evolved in bacteria and archaea to identify and destroy invading agents such as bacteriophages or plasmids (13). The commonly used Cas9 is from *Streptococcus pyogenes* (Sp), which we used in this study.

DSBs created by endonucleases are primarily repaired by non-homologous end joining (NHEJ) or homology-directed repair (HDR) (6,14). In the absence of a template, the NHEJ pathway is utilized, introducing variable insertions or deletions (indels) at the DSB site, which may disrupt the open reading frame of the gene and generate a knockout (KO) allele. This editing approach is relatively efficient and has been widely used in genetic engineering and functional genomics research (15,16). In the presence of a donor template flanked with homology arms (HAs), the HDR pathway can be used to integrate the sequence between HAs to create a precise DNA deletion, substitution, or insertion, leading to the correction of pathologic genes or the targeted integration of a gene or DNA fragment of interest. Unfortunately, HDR-mediated knockin (KI) using a conventional plasmid template is typically inefficient. Recently, we reported a 5- to 10-fold increase in HDR KI efficiency by using a double cut donor plasmid design, which is a conventional targeting vector flanked on either side by a Cas9–single guide RNA (sgRNA) recognition sequence (17). We also found that HAs of 300–600 bp in length are sufficient to guide precise genome editing. This finding has been independently reproduced in other labs (18,19). A similar gene targeting strategy that takes advantage of the highly efficient double cut HDR donor design (pDonor-sg) is used in this study.

Although efficient genome editing has been achieved in many tumor cell lines (12,20), efforts to precisely insert a large fragment into the genome of human pluripotent stem cells (PSCs) have been challenging. HDR efficiencies of 0.1–1% after creating DSBs using artificial nucleases have been reported by different labs (21–23). Up to 5% HDR insertion of a fluorescent protein in human iPSCs has been reported, but this is cell line-dependent (24).

The inefficiency in editing human PSCs is largely due to low cell viability after manipulation. In contrast to mouse PSCs, the dissociation of human PSCs into a single cell suspension often induces massive cell death. The use of a ROCK inhibitor considerably increases cloning efficiency (25) by preventing anoikis, which is dissociation-induced apoptosis (26,27). This has solved, however, only one problem. To precisely edit PSCs, the CRISPR components Cas9 and sgRNA, together with a DNA donor template, need to be delivered into cells. The most efficient way for vector delivery is electroporation or its improved version nucleofection (28,29), which still induces massive cell death (30,31). Furthermore, electroporation of DNA causes additional cell death, as previously reported in multiple cell lines (32–34). Although Cas9–sgRNA can be delivered in the less toxic forms of protein and RNA, the introduction of donor templates using plasmid vectors is the simplest approach, especially for the insertion of a large DNA fragment.

As massive cell death during and following nucleofection of plasmid vectors remains a major barrier in iPSC genome editing, we hypothesized that minimizing cell death during this process would considerably improve genome editing efficiency. Previous reports have shown that viral vector-mediated stable or transient overexpression of BCL2 or BCL-XL increases human PSC single cell survival (35,36). While studying cellular reprogramming and genome editing (37–39), we fortuitously found that overexpression of BCL-XL in iPSCs may greatly reduce cell death after electroporation, accompanied with a striking increase in editing efficiency. BCL-XL, the isoform 1 or anti-apoptotic isoform of BCL2-like 1 (BCL2L1) gene, maintains the integrity of the outer mitochondrial membrane and prevents the release of mitochondrial contents such as cytochrome *c*, an activator of apoptosis (40). Other anti-apoptotic factors in the BCL2 family include BCL2 and MCL1, whose roles in anti-apoptosis have been extensively studied (41). However, there are no reports to date on their roles in genome editing of human iPSCs or other cell lines.

In this study, we report a striking effect of BCL-XL on enhancing genome editing efficiency in human iPSCs. We find that co-transfection of BCL-XL expressing plasmids leads to ∼10-fold increase in iPSC survival after electroporation, as well as a 20- to 100-fold increase in HDR KI efficiency and ∼5-fold increase in NHEJ KO efficiency at multiple loci. Treatment of BCL-XL transfected iPSCs with the BCL inhibitor ABT-263 (42) further increases KI efficiency by 70% and KO efficiency by 40%. Similar results were obtained at multiple loci (*PRDM14, CTNNB1, OCT4, CD326*, and *CD9*) in six iPSC lines from different donors, demonstrating the reproducibility of this approach. HDR KI of a fluorescent reporter has been achieved in 10–50% of iPSCs without selection. The use of a simple selection strategy, i.e. dual or biallelic editing followed by the selection of drug-resistant cells, can achieve KI efficiency of 95% and KO efficiency of 100%. The unprecedented editing efficiency in a population of unselected iPSCs may enable one to avoid the tedious single cell cloning step in some applications, such as when creating reporter iPSC lines. Even for applications that need isogenic pairs to evaluate the effect of gene mutations in a given disease phenotype, one only needs to screen a small number of clones instead of hundreds of single cell clones.

## MATERIALS AND METHODS

### Lentiviral BCL-XL vector construction and virus production

The complementary DNA (cDNA) for the puromycin resistance gene (Puro) and BCL-XL were amplified by polymerase chain reaction (PCR) using KAPA HiFi polymerase (KAPA Biosystems) and purified using the GeneJET Gel Extraction Kit (Thermo Fisher Scientific). The fragments BCL-XL, E2A linker, and Puro were inserted into a lentiviral vector with the EF1 promoter using the NEBuilder HiFi DNA Assembly Kit (New England Biolabs). All constructs were verified by Sanger sequencing (MCLAB). All the correct clones were grown in CircleGrow Medium (MP Biomedicals) and DNA plasmids were purified using Endo-Free Plasmid Maxi Kits (Qiagen). A standard calcium phosphate precipitation protocol was used for lentivirus production. The lentiviral vectors were concentrated 100-fold by centrifugation at 6,000 g for 24 hours at 4°C to reach biological titers of 2–10 × 10^7^/ml.

### sgRNA design

The sgRNA vectors were constructed as detailed previously (43). In brief, we used the CHOPCHOP website (https://chopchop.rc.fas.harvard.edu/) (44) to design high-performance sgRNAs targeting *GFP* (sgDocut), human *PRDM14, CTNNB1, OCT4, CD9, CD326, BBC3, BAX, EEF1A1, GAPDH*,*AAVS1*, and mouse *EEF1A1*. The sgRNAs with a base G at the 5′ end, which initiates U6-promoter-mediated transcription, were preferentially chosen. The sgRNAs used in this paper are listed in Supplementary Table S2.

### Plasmid construction

All Cas9, sgRNA, BCL-XL, BCL2, and MCL1 plasmids were constructed with the NEBuilder HiFi DNA Assembly Kit (New England Biolabs). First, PCR products were produced using KAPA HiFi polymerase (KAPA Biosystems) and purified using the GeneJET Gel Extraction Kit (Thermo Fisher Scientific). The linear PCR products were then assembled into plasmids in a DNA assembly reaction (20 μl) on ice, according to the manufacturer’s instructions. The reaction contained NEBuilder HiFi DNA Assembly Master Mix (10 μl), equal ratios of PCR products (0.2–0.5 pM), and water. The ligation reaction was briefly vortexed and centrifuged prior to incubation at 50°C for 5–30 minutes. NEB 5-α Competent *Escherichia coli* cells were then transformed with the assembled DNA products and plated on LB agar with ampicillin. Multiple colonies were chosen for Sanger sequencing (MCLAB) to identify the correct clones.

### Construction of pDonor-sg double cut donor plasmids

The pDonor-sg vectors were constructed as detailed previously (43). The double cut donor plasmids used in this study were generated using the NEBuilder HiFi DNA Assembly kit (New England Biolabs), as detailed above. In short, all the fragments included in a pDonor-sg (left HA, desired KI fragments, right HA) were amplified by PCR using KAPA HiFi polymerase (KAPA Biosystems) and purified using the GeneJET Gel Extraction Kit (Thermo Fisher Scientific). The HA sequences ∼600 bp in length were amplified from human genomic DNA, and a sgDocut (donor cut) recognition sequence was added upstream of the left HA and downstream of the right HA. All the vectors were verified by Sanger sequencing (MCLAB).

### Human iPSC culture

The iPSC lines were generated from different anonymous adult donors by peripheral blood (PB) reprogramming using episomal vectors that express OCT4, SOX2, MYC, KLF4, and BCL-XL (37,38,45). Blood samples were obtained from the Tianjin Blood Center with the approval of the local research ethics committee. All feeder-free iPSCs (passages 6–15) were routinely maintained on Matrigel (Corning)-coated tissue-culture plates (BD) in mTeSR1 (Stemcell Technologies) (46,47). On the first day after passaging with Accutase (Stemcell Technologies), 10 μM ROCK inhibitor Y-27632 (Millipore) was added to the culture medium. Human iPSCs were cultured at 37°C with 5% CO_2_ and were fed with fresh medium every day.

### iPSC-BCL-XL cell line establishment

iPSCs were transduced with lentiviral vectors (Lenti-EF1-BCL-XL-E2A-Puro-Wpre) at a low multiplicity of infection (MOI) of 0.1–0.2, and stably transduced cells were selected by culturing them in mTeSR1 medium supplemented with 1 μg/ml puromycin for one week.

### Electroporation of human iPSCs

For genome editing in human iPSCs, cells were transfected by electroporation using the Amaxa Human Stem Cell Nucleofector^®^ Kit 2 (Lonza) and the program B-016, according to the manufacturer’s instructions. Briefly, a 70 μl electroporation solution was prepared for each reaction, including 57.4 μl of the nucleofector solution, 12.6 μl of the supplement, and plasmids. Generally 1 μg of Cas9 plasmid, 0.5 μg of sgRNA plasmid, 0.5 μg of sgDocut plasmid (for cutting pDonor in some experiments), 1 μg of pDonor plasmid, and 0.5 μg of BCL2 family gene plasmids were used. iPSCs with 60–70% confluency were used for electroporation. iPSCs were dissociated with the addition of 500 μl of Accutase, gently pipetted less than three times, and filtered with a 70 μm filter to obtain a single cell suspension. Approximately 1–1.5 × 10^6^ cells were centrifuged at 400 g for 5 minutes and the supernatant was carefully aspirated by vacuum. The cells were then resuspended in the 70 μl electroporation solution and carefully transferred into the cuvette. Electroporation was conducted on an Amaxa Nucleofector II. After electroporation the cuvette was incubated at 37°C for ∼5 minutes since we found that this improved cell survival, similar to a previous report (48). The cells were then seeded onto Matrigel-coated plates in mTeSR1 medium with 10 μM ROCK inhibitor Y-27632. Cells were gently handled during each step to reduce physical damage to the cells. One day later cultures were fed with fresh mTeSR1 medium without the ROCK inhibitor.

### Flow cytometry

Attached cells were dissociated with Accutase to obtain a single cell suspension and analyzed on a BD FACSAria III flow cytometer. For suspended cells, 80% of cells from the culture medium were transferred into a 5 ml FACS tube and analyzed directly. Cells were first gated for the intact cell population using forward scatter versus side scatter plots, and then gated for single cells based on forward scatter W versus forward scatter H. For HDR-mediated KI of a fluorescent reporter into a target gene (*PRDM14, CTNNB1, OCT4, EEF1A1*, and *GAPDH*), the fluorescence-positive cell population was considered the HDR KI cells. For NHEJ-mediated KO of a target gene (*CD9* and *CD326*), 1 μl of Anti-Human CD9 FITC (eBioscience) or Anti-Human CD326 PE (eBioscience) was added into a 100 μl single cell suspension (1–5 × 10^5^ cells), incubated for 20 minutes in the dark at room temperature to stain cells, washed with 2 ml of FACS buffer, and resuspended in 300 μl of FACS buffer for FACS analysis. The fluorescence-negative cell population was gated and labeled as KO cells. Electroporation without relevant sgRNAs was carried out as negative controls.

### Dynamics of BCL-XL expression at mRNA and protein levels after electroporation

To easily detect the expression levels of BCL-XL at both the messenger RNA (mRNA) and the protein levels, the pEF1-BCL-XL-mNeonGreen vector was constructed according to the protocol in the ‘Plasmid construction’ section. After transfection, the EF1 promoter drives expression of the BCL-XL-mNeonGreen fusion gene, and the fluorescence intensity detected by FACS accurately reflects the BCL-XL protein expression level. iPSCs were electroporated with 0.5 or 1 μg of pEF1-BCL-XL-mNeonGreen and the cells were harvested at 4, 6, 8, 16, 24, 48, and 72 hours after electroporation for flow cytometry and reverse transcription quantitative polymerase chain reaction (RT-qPCR) analysis. These methods are detailed in the ‘Flow cytometry’ and ‘RT-qPCR analysis’ sections, respectively.

### Dynamic changes of editing plasmid copy numbers after electroporation

iPSCs were harvested at 2, 4, 6, 8, 24, 72 hours, or 7 days after electroporation of CRISPR plasmids with or without BCL-XL. Genomic DNA was extracted using the Gentra Puregene Blood Kit (Qiagen) with RNase digestion. Amplification and detection were carried out on a 7500 Fast Real-Time qPCR System (Applied Biosystems) in 96-well plates. Each 20 μl reaction system was prepared containing 10 μM primer pairs (detailed below), 10–20 ng of DNA templates, 10 μl of KAPA SYBR^®^ FAST Universal 2x qPCR Master Mix (Kapa Biosystems), 0.4 μl of ROX Low (50x), and nanopure water. The reaction procedure was as follows: hold for 20 seconds at 95°C (enzyme activation) followed by 40 cycles of 3 seconds at 95°C (denaturation) and 30 seconds at 60°C (annealing, extension, and data acquisition). The melting curve was determined after PCR cycling to reassure the specificity of PCR amplification. A non-target control was included to rule out DNA carryover. The following primers were used to examine gDNA (ACTB), total plasmid (Backbone), BCL-XL plasmid, Cas9 plasmid, sgRNA plasmid, and pDonor plasmid (mNeonGreen): ACTB-F: TCGTGCGTGACATTAAGGAG, ACTB-R: GGCAGCTCGTAGCTCTTCTC; Backbone-F1: ATCCTGTTACCAGTGGCTGC, Backbone-R1: CGCTTACCGGATACCTGTCC; BCL-XL-F1: TCCCCATGGCAGCAGTAAAG, BCL-XL-R1: AAAAAGGCCA CAATGCGACC; Cas9-F1: CAGACAGCAACTGCCTGAGA, Cas9-R1: TGTCGAAAGTGCGCTGTTTG; sgRNA-F1: GCAAGTTAAAATAAGGCTAGTCC, sgRNA-R1: CGACTCGGTGCCACTTTTTC; mNeonGreen-F1: CATCAACGGTGTGGACTTTG, mNeonGreen-R1: GTATCCGGAGCCATCTACCA.

### Time-lapse imaging

After electroporation, iPSCs were seeded onto a Matrigel-coated 12-well plate (BD) with 2 ml of mTeSR1 medium and 10 μM ROCK inhibitor Y-27632. The plate was placed in the Biostation Cell Culture Observation System (Nikon) at the University of California Riverside (UCR) Stem Cell Core. When the condensation disappeared one hour later, one spot was chosen from each well for time-lapse imaging. Phase-contrast images were taken under the 20x objective at 10-minute intervals for a duration of 48 hours. Culture medium was not changed during the process so as to minimize perturbations. The time-lapse images were composed into videos with the iMovie software (Supplementary Movies S1–6).

### RT-qPCR analysis

Total RNA was extracted using an RNA Isolation Kit (Exiqon) with a DNaseI (Qiagen) treatment on the column. The RNA was quantified using the NanoDrop 2000 (Thermo Scientific) and diluted to the same concentrations. First-strand cDNA was synthesized from the same volume of RNA (500–1000 ng) using a 5x All-In-One RT MasterMix (Abm) in a 20 μl reaction system, according to the manufacturer’s instructions. cDNA was then diluted to 100 μl by adding 80 μl of nanopure water into each sample, and 5 μl was used for the next qPCR step. Amplification and detection were carried out on a 7500 Fast Real-Time qPCR System (Applied Biosystems) in 96-well plates with three parallel wells for each condition. Each 20 μl reaction system was prepared containing 10 μM gene-specific primer pairs (detailed below), 5 μl of diluted cDNA templates, 10 μl of KAPA SYBR^®^ FAST Universal 2x qPCR Master Mix (Kapa Biosystems), 0.4 μl of ROX Low (50x), and nanopure water. The reaction procedure was as follows: hold for 20 seconds at 95°C (enzyme activation) followed by 40 cycles of 3 seconds at 95°C (denaturation) and 30 seconds at 60°C (annealing, extension, and data acquisition). The melting curve was determined after PCR cycling to reassure the specificity of PCR amplification. A non-target control was included to rule out DNA carryover. The following primers were used: GAPDH-F: GAGTCAACGGATTTGGTCGT, GAPDH-R: TTGATTTTGGAGGGATCTCG; Cas9-F1: CAGACAGCAACTGCCTGAGA, Cas9-R1: TGTCGAAAGTGCGCTGTTTG; Cas9-F2: GGCTACGCCGGATACATTGA, Cas9-R2: TGGGGGATGCTTCCATTGTC; sgRNA-F1: GCAAGTTAAAATAAGGCTAGTCC, sgRNA-R1: CGACTCGGTGCCACTTTTTC; sgRNA-F2: GCTAGAAATAGCAAGTTAAAATAAG, sgRNA-R2: CTCGGTGCCACTTTTTCAAGT; BCL-XL-F1: TCCCCATGGCAGCAGTAAAG, BCL-XL-R1: AAAAAGGCCACAATGCGACC.

### RNA sequencing

RNA sequencing was conducted to investigate the effects of BCL-XL on promoting iPSC survival. Integration-free iPSCs from three different blood donors were used. After the nucleofection of *PRDM14* editing vectors with or without the BCL-XL plasmid, cells were harvested at 2, 4, and 8 hours after electroporation. Total RNA was extracted from iPSCs using an RNA Isolation Kit (Exiqon) with DNaseI (Qiagen) treatment on the column. RNA sequencing was conducted by Novogene (Tianjin, China). In brief, 1 μg of total RNA from each sample was created into Illumina mRNA-seq libraries with a TruSeq RNA kit (version 1, set A) following polyA-selection, fragmentation, first-strand synthesis, and second-strand synthesis steps. The libraries were sequenced on an Illumina HiSeq x10. Approximately 20 million reads of 150 bp paired-end data from each sample were obtained. The data were analyzed using the web-based platform Galaxy. Salmon (49) was used to determine transcripts per million (TPM) by aligning the data with the human transcriptome (Gencode Release 19, GRCh37.p13). DESeq2 was used to identify differentially expressed genes (DEGs) between BCL-XL groups and the control group without BCL-XL (50). Those DEGs were further analyzed for pathway enrichment using DAVID (51) and Ingenuity Pathway Analysis (IPA) .

### BBC3 and BAX knockout iPSC lines

The pD-EF1-Puro-PolyA-sg plasmid was designed and constructed as described previously. Two human iPSC lines were electroporated with plasmids that express Cas9, sgBBC3 (or sgBAX), pD-EF1-Puro-PolyA-sg, and BCL-XL, as described previously. The integration of the EF1-Puro-PolyA expression cassette at the cleavage site leads to the disruption of the open reading frames of *BBC3* or *BAX* and the expression of the Puro resistance gene. Puromycin (1 μg/ml) was added into culture medium for one week to select stable *BBC3* or *BAX* KO cell lines.

### Small molecules

To test the effects of small molecule compounds, iPSCs were equally split into several wells after electroporation with CRISPR plasmids. Pan-caspase inhibitor Z-VAD-FMK (Sigma), TP53 inhibitor pifithrin-α (PFT; Sigma), and BCL inhibitor ABT-263 (Navitoclax; Selleck Chemicals) were first diluted in 50 μl of culture medium to make a master mix. Then 50 μl of the diluted small molecules were added evenly into each well with the desired working concentration and treatment time. The medium was changed with fresh medium thereafter. A parallel well with only DMSO added (0.1%) was carried out as a control. Three days after electroporation, the cells were harvested for FACS analysis to determine editing efficiency.

### Analysis of NHEJ/HDR editing and off-target cleavage by deep sequencing or Sanger sequencing

To compare editing efficiencies of different donor designs, double cut donor pDonor-sg or single-stranded oligodeoxynucleotides (ssODNs) were used (17,52). Three iPSC lines were electroporated with 1 μg of Cas9 plasmid, 0.5 μg of sgRNA plasmid, with or without 0.5 μg of pEF1-BCL-XL or pEF1-BCL2, together with different donor templates. For editing at *EEF1A1* or *GAPDH*, 1 μg of pDonor-sg-PmeMul was used which induces a 12 bp insertion and 6 bp deletion after HDR editing. For editing at *AAVS1, CD326*, and *GAPDH*, 0.5 μl of 40 pM ssODN template (AAVS1–50Mlu50-ssODN, CD326a-50Mlu50-ssODN, or GAPDH-50Mlu50-ssODN) was used, each of which induces a 6 bp insertion after HDR editing. The Ultramer^®^ DNA single-stranded oligonucleotides AAVS1-ssODN and CD326a-ssODN were purchased from IDT. The sequences of these ssODNs are as follows: G*G*GTACTTTTATCTGTCCCCTCCACCCCACAGTGGGGCCACTAGGGACAGacgcgtGATTGGTGACAGAAAAGCCCCATCCTTAGGCCTCCTCCTTCCTAGTCT*C*C (*AAVS1*), C*G*CGCGCAGCATGGCGCCCCCGCAGGTCCTCGCGTTCGGGCTTCTGCTTGacgcgtCCGCGGCGACGGCGACTTTTGCCGCAGCTCAGGAAGGTGAGGCGCGGA*T*T (*CD326*), and A*A*GCTCATTTCCTGGTATGTGGCTGGGGCCAGAGACTGGCTCTTAAAAAGacgcgtTGCAGGGTCTGGCGCCCTCTGGTGGCTGGCTCAGAAAAAGGGCCCTGA*C*A (*GAPDH*). * indicates phosphorothioate (PS) modification (53) which increases oligo stability, and the underlined sequence indicates targeted insertion.

iPSCs treated in different conditions were harvested three days after electroporation for genomic DNA extraction using the Gentra Puregene Blood Kit (Qiagen) with RNase digestion. To prevent artifacts induced by plasmid templates when editing at *EEF1A1* or *GAPDH*, the primary PCR was conducted using primers targeting genomic sequences flanking the HAs of the donor. PCR was conducted with KAPA HiFi DNA polymerase. For primary PCR, the following primers were used: EEF1A1-F1: CCACCAACTCGTCCAACTGA, EEF1A1-R1: CCCACGTTTCAACATGCACA; GAPDH-F1: ATGTTCGTCATGGGTGTGAA, GAPDH-R1: CCAGGCTGAGCTCCACTAAC. The PCR cycling condition was as follows: 95°C hold for 4 minutes, followed by 30 cycles of 98°C for 5 seconds, 64°C for 5 seconds, 68°C for 5 seconds, and 72°C for 30 seconds. Off-target site prediction was performed using the COSMID tool (54). Primers targeting off-target sites were designed by Primer3Plus to have an expected amplicon between 250-285 bp (Supplementary Table S3). The PCR cycling conditions was as follows: 95°C hold for 4 minutes, followed by 30 cycles of 98°C for 5 seconds, 64°C for 5 seconds, 68°C for 5 seconds, and 72°C for 5 seconds. The following primers were used to amplify DNA for deep sequencing: EEF1A1-F2: CCACCTTTGGGTAAGGATGA, EEF1A1-R2: GAGTGGGGTGGCAGGTATTA; GAPDH-F2: CTGACTTCAACAGCGACACC, GAPDH-R2: GGTGGTCCAGGGGTCTTACT; AAVS1-F2: CCCCTATGTCCACTTCAGGA, AAVS1-R2: GGGGGTGTGTCACCAGATAA; CD326-F2: GCTCCTCGTGTCCCACTC, CD326-R2: CTCTTGGTCCCCTCCCTATT.

The PCR products were confirmed by electrophoresis on 1% agarose gels. Approximately 50 ng of PCR products from each on-target and off-target sites were mixed and were sequenced on an Illumina HiSeq x10 by Novogene (Tianjin, China).

High-throughput sequencing data were analyzed using the Galaxy platform (55) and web-based tool Cas-Analyzer (56). We used FLASH (57) to merge double-ended data and split merged data with the Barcode Splitter tool on Galaxy. Next, the demultiplexed data were uploaded to Cas-Analyzer and executed according to a reference PCR sequence and sgRNA sequence. For clarity, we only showed the top 10 possibilities of on-target cleavage in representative samples. To analyze HDR efficiency, donor sequences were provided to Cas-Analyzer. This tool returns results including total read number, HDR read number and percentage, and indels reads (the sum of HDR and NHEJ).

Cells edited with GAPDH-50Mlu50-ssODN were analyzed with Inference of CRISPR Edits (ICE), a web-based analysis tool developed by Synthego (https://www.biorxiv.org/content/early/2018/01/20/251082). In brief, iPSCs were harvested 3 days after nucleofection for PCR amplification, followed by Sanger sequencing. The .ab1 files were uploaded to https://ice.synthego.com/#/ for analysis. The ICE score indicates editing by NHEJ and HDR, and the +6 insertion indicates HDR editing.

### Digital karyotyping by SNP arrays

Genomic DNA samples were extracted from indicated iPSCs and tumor cell lines, and were hybridized to HumanCoreExome arrays (Illumina; 20005132) followed by staining and scanning on the Illumina HiScan system, per standard protocol (58). These arrays can interrogate 550,601 human SNP markers, thus yielding up to 50-fold better resolution (∼100 kb) than conventional karyotyping by Giemsa banding. Log *R* ratio (LRR) and B allele frequency (BAF) were used to detect copy number variants (CNVs). LRR represents logged ratio of observed probe intensity to expected intensity, with any deviations from 0.0 indicating copy number changes. BAF is the proportion of hybridized sample that carries the B allele, as designated by the Infinium assay, so that discrete BAF values of 0.0, 0.5, and 1.0 for each locus (representing AA, AB, and BB) can be seen in a normal sample.

### Mouse ESC culture and electroporation

Mouse ESCs were purchased from ATCC and maintained in Dulbecco’s modified Eagle’s medium (DMEM) supplemented with 5% fetal bovine serum (FBS; Gibco), 5% serum replacement (Gibco), 2 mM glutamine (Gibco), 10 ng/ml mouse leukemia inhibitory factor, 0.1 mM 2-mercaptoethanol (Gibco), 3 μM GSK inhibitor CHIR99021 (Selleck), 1 μM MEK inhibitor (MCE^®^), and 1% penicillin/streptomycin (Invitrogen). Cells were then seeded on 0.1% gelatin-coated (Sigma) tissue-treated 6-well plates. The cells were fed with fresh medium every day and split by 6- to 8-fold every 2–3 days. The electroporation process performed was similar to that used for human iPSCs, except the A-013 program was used.

### K562 cell culture and electroporation

K562 cells (ATCC; CCL-243) were grown in RPMI-1640 medium (VWR Life Science) with 10% FBS (Gibco) and 1% penicillin/streptomycin (Invitrogen). For the electroporation of K562 cells, the Amaxa™ Cell Line Nucleofector™ Kit V (Lonza) and T-016 program were used according to the manufacturer’s instructions.

### Jurkat cell culture and electroporation

Jurkat cells (ATCC; Clone E6–1) were grown in RPMI-1640 medium (VWR Life Science) with 10% FBS (Gibco) and 1% penicillin/streptomycin (Invitrogen). For the electroporation of Jurkat cells, the Amaxa™ Cell Line Nucleofector™ Kit V (Lonza) and X-001 program were used according to the manufacturer’s instructions.

### 293T cell culture and transfection

HEK293T cells were cultured in DMEM supplemented with 10% FBS (Gibco) and 1% penicillin/streptomycin (Invitrogen). For the transfection of HEK293T cells, Lipofectamine 3000 (Life Technologies) was used according to the manufacturer’s instructions.

### Statistics

The *P* values for different groups were calculated and analyzed by paired student’s *t*-test. *, *P* < 0.05; **, *P* < 0.01; ***, *P* < 0.001; ns, not significant.

## RESULTS

### Electroporation of plasmids leads to massive cell death in iPSCs

**Figure 1.**
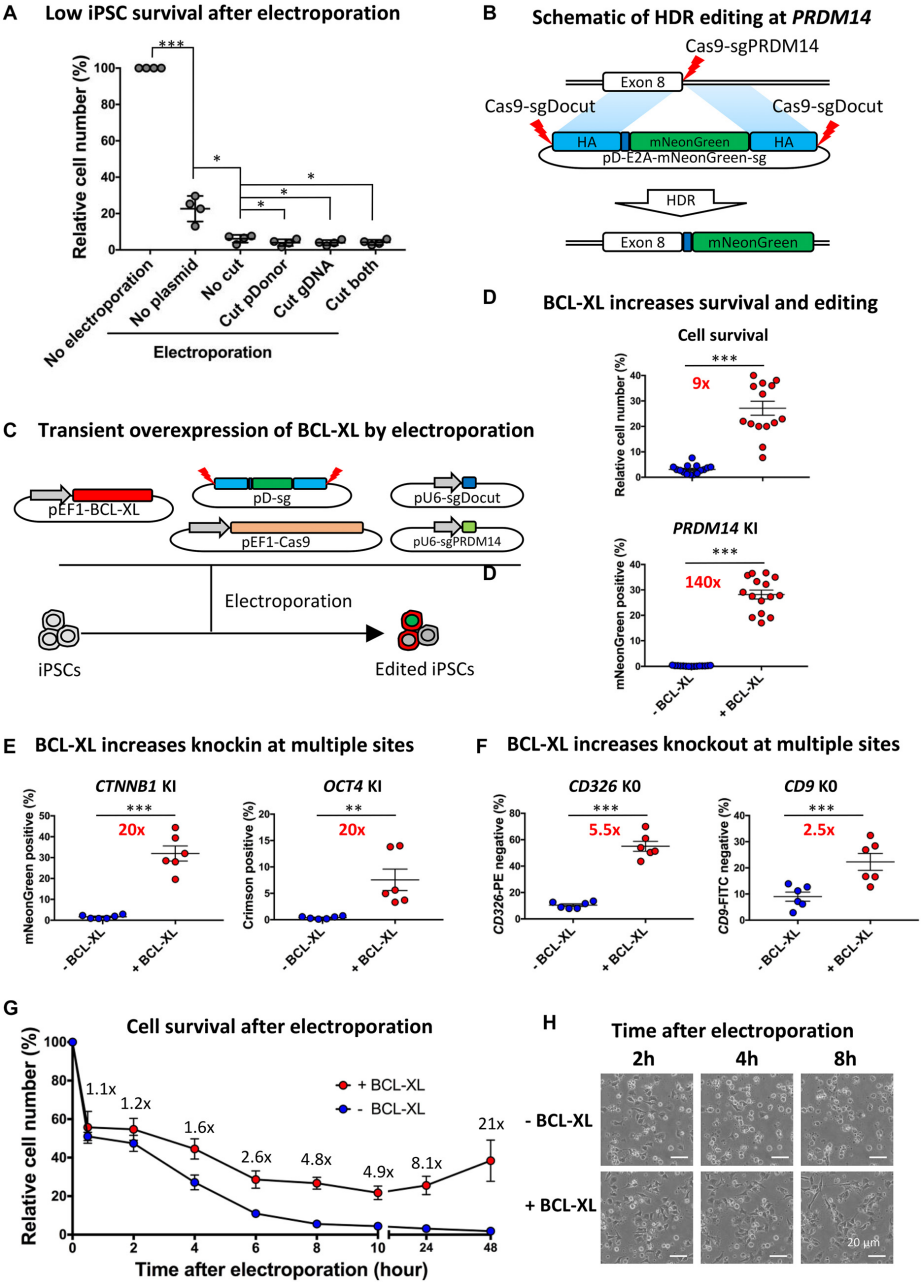
BCL-XL increases editing efficiency by promoting cell survival after electroporation. (**A**) Relative iPSC number on day 1 after electroporation with or without *PDRM14* editing plasmids. To determine the effects of cutting just genome or pD, sgDocut or sgPDRM14 were omitted, respectively; *n* = 4, *P < 0.05, ***P < 0.001. (**B**) Schematic of HDR-mediated editing at *PRDM14*. An sgPRDM14 was designed to target the stop codon. A promotorless double cut HDR donor pD-PRDM14-E2A-mNeonGreen-sg was used to guide HDR insertion of the mNeonGreen reporter. E2A is a self-cleaving linker for multicistronic expression. Left and right HA: light blue (600 bp); E2A: blue; Cas9–sgRNA cleavage site: red lighting. (**C**) Transient BCL-XL overexpression is achieved by transfection with a pEF1-BCL-XL plasmid. sgPRDM14 was designed to cut *PRDM14*, and sgDocut for cutting pD-sg. (**D**) Transient BCL-XL overexpression strikingly increases human iPSC survival and HDR efficiency at *PRDM14* locus. HDR efficiency was determined 3 days after transfection by FACS; *n* = 15, ***P < 0.001. (**E**) Transient BCL-XL overexpression strikingly increases HDR-mediated KI efficiency at both *CTNNB1* and *OCT4*; *n* = 6, **P < 0.01, ***P < 0.001. (**F**) Transient BCL-XL overexpression increases NHEJ-mediated KO efficiency at both *CD9* and *CD326*. KO efficiency was determined 1 week after transfection by FACS; *n* = 6, ***P < 0.001. (**G**) Dynamic changes in relative cell numbers after electroporation with genome editing plasmids with or without BCL-XL; *n* = 6, data presented as mean ± SEM. (**H**) Representative images of iPSCs at 2, 4, or 8 hours after electroporation with or without BCL-XL. Without BCL-XL, massive cell death was observed at 4 or 8 hours after electroporation. For details, see Supplementary Movies S1–6.

Massive cell death is often observed when electroporation is carried out on iPSCs with plasmids, presenting a bottleneck in improving editing efficiency. We reasoned that the high death rate may be attributable to the (i) single cell preparation of iPSCs; (ii) instantaneous cell death resulting from electric shock; (iii) cytotoxicity of plasmids; and/or (iv) cleavage of the genome. To distinguish between these possibilities, we compared the cell survival rates on day 1 for 1–1.5 × 10^6^ iPSCs under the following conditions: no electroporation, electroporation with or without plasmids, and electroporation with or without DNA cleavage (Figure 1A). All groups followed the same cell preparation and electroporation parameters. iPSCs without electroporation survived very well under the protection of the ROCK inhibitor Y-27632, for this reason we set it as a control.

Compared to the control group without electroporation, 75% of iPSCs died after electroporation treatment alone (Figure 1A). We suspect this may be due to irreversible membrane damage induced by the high-voltage electric shock, resulting in instantaneous necrosis of some cells (59). This is supported by the observation of aggregate clumps of cellular debris floating in the medium after electroporation, and the decreased proliferation of cells that survive electroporation. Strikingly, electroporation with plasmids resulted in more severe cell death with only ∼5% cell survival rate, representing a 5-fold decrease in survival compared to no plasmid treatment. In addition, we observed further decreased cell survival when the Cas9–sgRNA construct expressed by the plasmids induced a cleavage of pDonor-sg and/or the genome (Figure 1A). These results demonstrate that the electroporation procedure and plasmid cytotoxicity are the two main factors leading to the massive cell death of iPSCs during genome editing, whereas the DNA damage response after Cas9–sgRNA mediated cleavage only plays a minor role.

### Stable BCL-XL overexpression increases editing efficiency by promoting cell survival after electroporation

In our previous study on HDR editing using double cut HDR donors (17), we observed relatively high levels of cell survival and editing efficiency after electroporation of iPSCs. These iPSCs were generated by lentiviral transduction of Yamanaka reprogramming factors together with BCL-XL (60,61). This result is in striking contrast to what we observed with integration-free iPSCs generated with episomal vectors (38), strongly suggesting that BCL-XL improves iPSC survival after nucleofection. To verify this assumption, we transduced three different integration-free iPSC lines with a lentiviral vector that expressed both BCL-XL and puromycin resistance gene (MOI = 0.1). iPSC-Lenti-BCL-XL lines that stably overexpressed BCL-XL were established following puromycin selection (Supplementary Figure S2A). As a control, iPSCs-Lenti-control lines were also established by transduction with the Puro resistance gene only.

We tested the effects of stable BCL-XL overexpression by using the previously verified *PDRM14* HDR editing vector system (17). CRISPR plasmids including pEF1-Cas9, pU6-sgPRDM14, pD-PRDM14-E2A-mNeonGreen-sg, and pU6-sgDocut were co-electroporated into iPSC-Lenti-BCL-XL lines and iPSC-Lenti-control lines, leading to the precise integration of E2A-mNeonGreen at the *PRDM14* stop codon after HDR editing. Since *PRDM14* is actively expressed in iPSCs, its endogenous transcription machinery drives expression of mNeonGreen, thus mNeonGreen-positive cells represent HDR-edited events (Figure 1B). Cell survival was determined by the number of live cells on day 1 relative to the number of cells used for electroporation. Similar to the previous study (Figure 1A), only 3% of iPSC-Lenti-control cells survived on day 1, whereas 90% of iPSC-Lenti-BCL-XL cells survived, suggesting that stable BCL-XL overexpression leads to a 30-fold improvement in cell survival after electroporation with plasmids (Supplementary Figure S2B). Gene editing efficiency at *PRDM14* was increased from 0.17% in the control group to ∼15% in the iPSC-Lenti-BCL-XL lines, a 90-fold improvement (Supplementary Figure S2B). These results suggest that increased iPSC survival by BCL-XL leads to enhanced HDR editing in surviving cells.

### Transient BCL-XL overexpression increases editing efficiency by selecting iPSCs harboring high copies of CRISPR plasmids

Stable BCL-XL overexpression may affect downstream applications of edited iPSCs, therefore we tested whether transient BCL-XL overexpression from a non-integrating vector could also achieve the same effects. For this purpose, we constructed the plasmid pEF1-BCL-XL encoding BCL-XL under the control of the EF1 promoter. We electroporated iPSCs with the pEF1-BCL-XL plasmid together with CRISPR plasmids composed of pEF1-Cas9, pU6-sgPRDM14, pDonor-sg, and pU6-sgDocut. (Figure 1C). To examine the dynamic changes of BCL-XL after electroporation, we created a BCL-XL-mNeonGreen fusion gene, allowing for measuring BCL-XL at the protein level by FACS and at the mRNA level by RT-qPCR. We observed high levels of BCL-XL expression 12–24 hours after transfection, followed by a significant decrease in BCL-XL at both the protein and mRNA levels (*P* < 0.05) (Supplementary Figure S3). These data demonstrate that transient transfection of plasmids only leads to 1–2 days of high-level expression.

Cell survival on day 1 and HDR KI efficiency on day 3 were examined, as detailed above. Compared with 3% cell survival in the control group without BCL-XL, 27% of cells in the transiently overexpressed BCL-XL group survived, a 9-fold increase (Figure 1D). The increased survival is accompanied with ∼150-fold improvement in KI efficiency (0.17 versus 25%) at the *PRDM14* locus (Figure 1D). Of note, transient BCL-XL overexpression exhibited higher editing efficiency compared to stable BCL-XL overexpression (25 versus 15%) (Figure 1D and Supplementary Figure S2B). We speculate this result may be attributed to greater selection pressure after transient BCL-XL transfection because cells harboring greater copy numbers of BCL-XL and editing plasmids are more likely to survive the stress and have higher editing efficiency.

To test this hypothesis, we transfected cells with editing plasmids (1 μg of Cas9, 1 μg of Donor plasmid, and 0.5 μg of sgRNA) with or without BCL-XL (0.5 μg), and examined dynamic changes of plasmid copy numbers per cell by qPCR (Supplementary Figure S4). As expected, the BCL-XL group had a relatively higher copy number (∼400 versus 300), although the difference was not statistically significant (paired *T* test). Of interest, the average copy number per cell in the BCL-XL group increased from ∼400 to 600 between 2 and 6 hours, suggesting that cells with high copies of BCL-XL are more likely to survive (Supplementary Figure S4A). In addition, copy numbers were maintained at ∼400 up until 24 hours after transfection, which can be explained by TP53 activation-induced cell cycle arrest (see the ‘RNA-seq analysis’ section). In contrast, between 6 and 24 hours, the copy numbers in the control group without BCL-XL abruptly dropped from ∼300 to 30, suggesting a strong selection against iPSCs carrying more plasmids (Supplementary Figure S4B). This result is consistent with massive cell death observed between 6 and 24 hours in the control group (Figure 1G). In the BCL-XL group, the copy number decreased by ∼3-fold from day 1–3, largely due to the dilution effects of cell proliferation. One week after transfection, the residual plasmids were barely detectable (Supplementary Figure S4A). As expected, the dynamic changes of all the CRISPR component plasmids followed the same pattern in each group. Together these data consolidate the conclusion that addition of BCL-XL promotes the survival of plasmid-transfected iPSCs, abrogating the negative selection against high-copy cells. As a result, the relatively higher levels of CRISPR components in surviving cells lead to efficient editing.

To generalize the effects of BCL-XL on iPSC survival and HDR-mediated KI, we conducted similar experiments at two other loci, *CTNNB1* and *OCT4*. First we conducted a dose-finding experiment and found that as little as 0.1 μg of BCL-XL plasmid had a considerable effect in promoting iPSC survival and enhancing editing, while increasing the dose from 0.25 to 1 μg of BCL-XL plasmid tended to increase the beneficial effects. In contrast, further increasing the dose to 2 μg showed a trend of decreased effects (Supplementary Figure S5). As such, 0.5 ug of BCL-XL plasmid was used in the following experiments. Gene editing strategies at *CTNNB1* and *OCT4* are illustrated in Supplementary Figure S6A and B, respectively. After HDR KI of the promoterless pDonor vector, expression of the fluorescent reporter mNeonGreen or Crimson was driven by the endogenous *CTNNB1* or *OCT4* transcription machinery. To further increase reproducibility of our studies, we carried out these experiments using six different iPSC lines. Again, BCL-XL co-transfection led to a dramatic improvement in KI efficiency of ∼20-fold at both *CTNNB1* (1.6 versus 32%) and *OCT4* (0.4 versus 7.6%) (Figure 1E; Supplementary Figure S6A and B).

We reasoned that BCL-XL increases the survival of stressed iPSCs after electroporation, and thus should also increase the efficiency of genome editing other than HDR KI. Therefore we investigated the effects of BCL-XL on NHEJ-mediated KO at *CD326* (also known as *EPCAM*) and *CD9*. We chose to knockout these two genes because they are expressed at high levels in human iPSCs and can be detected by antibody staining of surface markers, followed by flow cytometry. Experiments were carried out in six different iPSC lines, and gene editing strategies are illustrated in Supplementary Figure S6C and D. As expected, we observed significant improvement in KO efficiency by BCL-XL, ∼5.5-fold increase at *CD326* (10 versus 55%) and ∼2.5-fold increase at *CD9* (9 versus 22%) (Figure 1F; Supplementary Figure S6C and D). Interestingly, we observed considerably lower improvement from BCL-XL in NHEJ KO versus HDR KI, which may be due to higher baseline editing levels in KO versus KI without BCL-XL (∼10 versus 0.2–1%). The discrepancy between NHEJ and HDR repair can be further explained by cell cycle arrest at G1 after the electroporation of plasmids induced stress and CRISPR cleavage induced TP53 activation, because cells at G1 with DSBs are predominantly repaired by NHEJ (62).

Taken together, these studies demonstrate that transient BCL-XL overexpression in human PSCs considerably increases both HDR knockin efficiency and NHEJ-mediated knockout efficiency.

### Analysis of cell death dynamics after electroporation with or without BCL-XL

To investigate the dynamics of cell death after the electroporation of iPSCs with plasmids in the absence or presence of BCL-XL, we counted live cells 0.5–48 hours post-transfection. About 0.5 hours after electroporation, ∼50% of iPSCs in both groups survived the shock. From 0.5–2 hours, cells in both groups stably attached and no additional cell death was observed (Figure 1G). However, from 2–8 hours, the vast majority of cells in the control group began to die. In contrast, the ever-increasing expression of BCL-XL (Supplementary Figure S3) protected cells from death, leading to a 60% increase in survival at 4 hours. Six hours later, with the accumulation of BCL-XL, cell death was not significantly increased henceforth. Twenty-four hours after electroporation with BCL-XL, cells began to divide (Supplementary Movies S1–6), leading to an increase in relative survival rates from ∼10- to 20-fold compared to the no BCL-XL controls (Figure 1G).

To consolidate and visualize the protective effects of BCL-XL after electroporation of iPSCs with plasmids, we conducted a time-lapse analysis (Supplementary Movies S1–6). Starting 2 hours after electroporation, many cells released multiple small apoptotic bodies indicating apoptosis, whereas a few cells lost viability with the remaining corpse, suggestive of necrosis. Some cells continued to die 8 hours later in the control group, but at a decreased rate. In contrast, the rate of cell death in the BCL-XL group from 2–8 hours was considerably lower. In addition, surviving cells in the BCL-XL group displayed healthy morphology and higher mobility compared with the no BCL-XL controls. Around 12 hours later, surviving iPSCs in the BCL-XL group actively communicated with each other by extending pseudopodia and clustered together. Approximately 24 hours later, a few cells started to divide and proliferate. Representative images of iPSCs at 2, 4, or 8 hours after electroporation (Figure 1H) were extracted from the time-lapse movies (Supplementary Movies S3 and 4). These time-lapse studies confirm that rapid cell death occurs 2–8 hours after electroporation, while overexpressed BCL-XL prevents cell death starting 4 hours after electroporation (Figure 1G).

### BCL2 or MCL1 is inferior to BCL-XL in iPSC survival and editing

**Figure 2.**
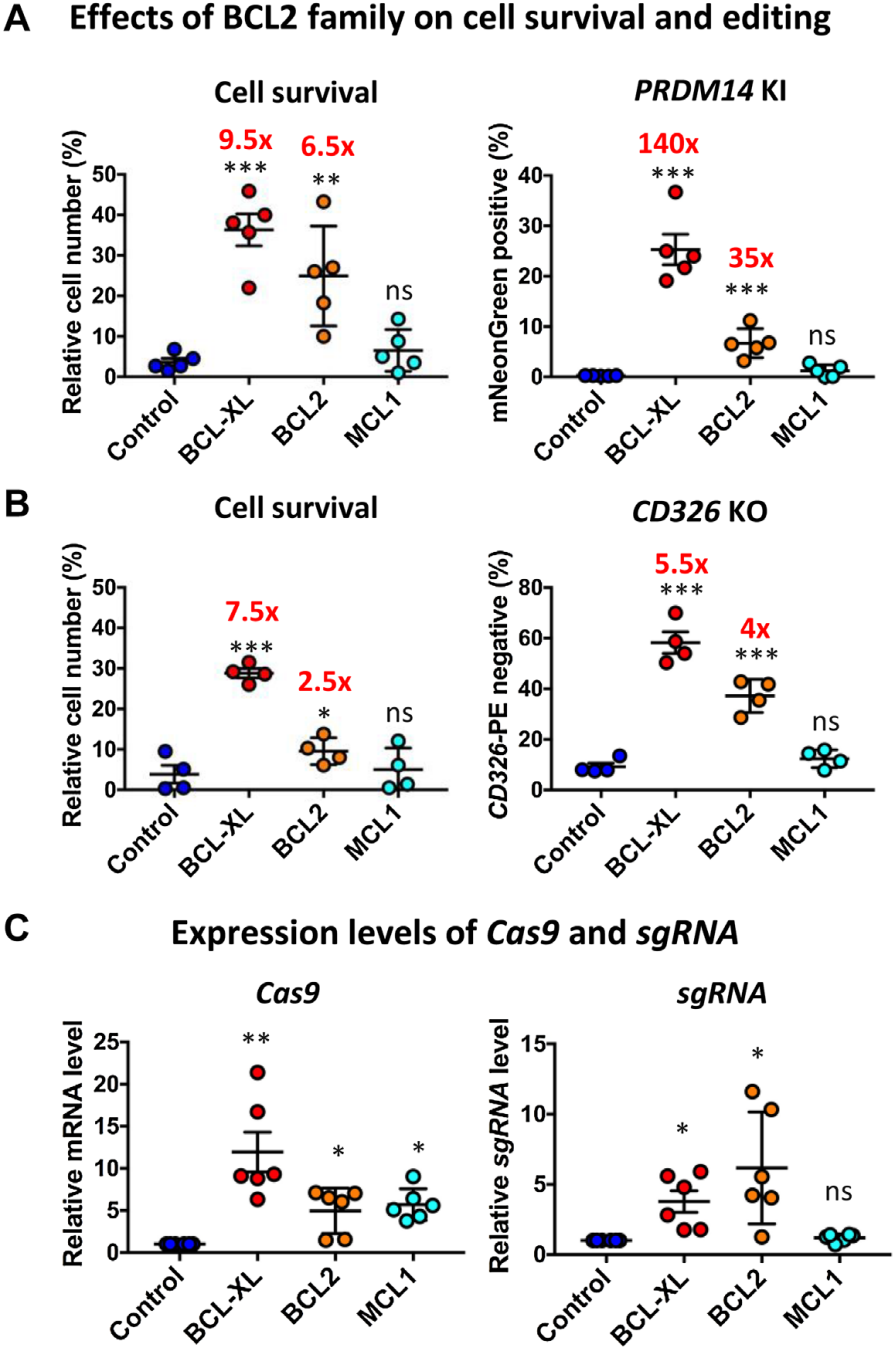
BCL2 or MCL1 is inferior to BCL-XL in promoting iPSC survival and editing efficiency. (**A** and **B**) Effects of BCL2 and MCL1 on human iPSC survival and editing. Cell survival was determined by cell count on day 1 after electroporation. KI or KO efficiency was determined on day 3 or 7 by FACS; *n* = 4–5. (**C**) Relative expression of Cas9 and sgRNA by RT-qPCR; *n* = 6. RT-qPCR analysis was conducted 8 hours after electroporation; *P < 0.05; **P < 0.01; ***P < 0.001; ns = not significant.

Having demonstrated the significantly beneficial effects of BCL-XL on iPSC survival and editing after electroporation, we asked whether other members in the BCL2 family could also be used to facilitate iPSC editing. For this purpose, we constructed pEF1-BCL2 and pEF1-MCL1 plasmids to compare with the pEF1-BCL-XL plasmid. We tested these vectors in both HDR KI and NHEJ KO systems. For the KO system, BCL2 improved survival rate ∼6-fold, but at lower levels compared to BCL-XL alone (∼9-fold), whereas MCL1 did not significantly increase iPSC survival after plasmid transfection. Similarly, BCL2 moderately increased HDR-mediated KI efficiency (0.2 versus 7.5%, ∼35-fold improvement), whereas MCL1 showed no significant improvement in both KI and KO efficiency (Figure 2A). For the KO system, BCL2 also improved survival rate by a lower fold compared to BCL-XL alone (∼2.5 versus ∼7-fold), whereas MCL1 showed no improvement in cell survival. Likewise, BCL2 only moderately improved NHEJ-mediated KO efficiency (9.2 versus 37%, ∼4-fold improvement) (Figure 2B). These data demonstrate that the striking effects of BCL-XL cannot be replaced by either BCL2 or MCL1.

BCL-XL or BCL2-mediated increase in editing efficiency is associated with increased cell survival (Figure 2A and B). The above studies show that BCL promotes the survival of iPSCs transfected with more copies of editing plasmids (Supplementary Figure S4), which is expected to increase RNA expression of Cas9–sgRNA. As expected, RT-qPCR analysis showed a 5 to 10-fold increase in *Cas9* and 3- to 6-fold increase in sgRNA levels (Figure 2C). Collectively, these data indicate that iPSCs carrying more plasmids are better protected from cell death by BCL.

### RNA-seq analysis reveals anti-apoptotic effects of BCL-XL

In an attempt to investigate the potential mechanisms underlying BCL-XL-mediated cell survival after electroporation of plasmids, we conducted RNA-seq analysis on samples harvested at 2, 4, or 8 hours when massive cell death was observed. At early time points, no DEGs were identified, likely due to low levels of BCL-XL (Supplementary Figure S7A). With considerably increased expression of BCL-XL at 8 hours (74-fold higher than control), 51 genes were upregulated (Supplementary Figure S7B). Consistent with the well-established function of BCL-XL as an anti-apoptotic factor, gene ontology (GO) analysis of these 51 genes revealed the enrichment of multiple biological processes, in particular the negative regulation of apoptosis, DNA damage response, and cellular response to DNA damage stimulus (Supplementary Figure S7C). These results are consistent with the well-established function of BCL-XL as a potent anti-apoptotic factor. The top 10 enriched biological processes also include DNA damage response (signal transduction by *TP53*) and cellular response to DNA damage stimulus (Supplementary Figure S7C). Furthermore, the analysis of regulatory network by IPA showed that BCL-XL overexpression is associated with enhanced synthesis and repair of DNA, in addition to reduced degradation, metabolism, and fragmentation of DNA (Supplementary Figure S7D).

While our work was in revision, two studies on TP53-mediated DNA damage response after CRISPR–Cas9 genome editing were published (63,64). Hence, we reanalyzed our RNA-seq data, focusing on TP53 target genes (65,66). Similar to the study on CRISPR–Cas9 engineering in human PSCs, we also found the upregulation of a similar set of TP53 target genes such as *BBC3, BTG2, CDKN1A, MDM2*, and *POLH* (Supplementary Figure S7E) (64). In our dataset, we identified additional TP53 target genes such as *ALOX5* (67), *GADD45A* (68), *KITLG* (69), *PGF*, and *TNFSF9* (Supplementary Figure S7E). These genes play critical roles in cell cycle arrest, apoptosis, and DNA damage repair. Most of these genes’ expressions followed the same pattern: (i) increased expression from 2–4 hours then decreased expression from 4-8 hours in the control group; (ii) continuous increase from 2–8 hours in the BCL-XL treated iPSCs. The transcriptome data provide further explanation to our experiment results. Some iPSCs in the control group showed evidence of DNA repair at 4 hours, but most of these cells died from the DNA damage stress after 8 hours. In contrast, BCL-XL protected the edited iPSCs from DNA damage-induced stress and TP53 activation, increasing survival. All of this led to strikingly increased editing efficiency.

### BCL-XL cannot be replaced by inhibition of pro-apoptotic factors

BCL-XL binds to pro-apoptotic counterparts BAX and BAK1, preventing the formation of lethal pores in the mitochondrial outer membrane, and thereby interrupting apoptosis (70). In human ESCs, BAX, not BAK1, is the key inducer of apoptosis (71). We also found that human iPSCs predominantly express BAX instead of BAK1 (∼300 versus 50 TPM) (Supplementary Table S1). We thus hypothesized that a *BAX* KO may promote the survival of stressed iPSCs. To this end, we established four *BAX* KO cell lines from two different iPSC lines by targeting Exon 1 or Exon 2 of *BAX* with Cas9–sgRNA. *BAX* KO in these four lines greatly improved cell survival and HDR-mediated KI at both the *PDRM14* and *CTNNB1* loci (Supplementary Figure S8A), but the KI levels were lower than those BCL-XL alone (Figure 1DE), suggesting that a *BAX* KO cannot replace BCL-XL.

BBC3, also known as PUMA, interacts with BCL2 family members, thus freeing BAX or BAK1 and inducing apoptosis (72). We asked whether a *BBC3* KO could increase survival and editing in iPSCs. Thus we established four *BBC3* KO cell lines by targeting Exon 1 or 2 of *BBC3* in two different iPSC lines. Similar to *BAX* KO, *BBC3* KO greatly improved cell survival and editing (Supplementary Figure S8B), but the effect is still inferior to that of BCL-XL overexpression (Supplementary Figure S6B), suggesting that a *BBC3* KO alone cannot replace BCL-XL either.

After the formation of mitochondrial outer membrane permeabilization (MOMP), cytochrome *c* is released from the mitochondria and triggers a caspase activation cascade, inducing apoptosis. We therefore tested if the pan-caspase inhibitor Z-VAD-FMK (73) could improve iPSC survival. Z-VAD-FMK treatment moderately increased iPSC survival and HDR efficiency. However, this effect of Z-VAD-FMK treatment is obviously inferior compared to that of BCL-XL overexpression (Supplementary Figure S8C).

Our findings that *BAX* KO, *BBC3* KO, or Z-VAD-FMK treatment did not significantly enhance the effects of BCL-XL (Supplementary Figure S8A–C), suggest that BCL-XL overexpression alone may achieve maximum protective effects. These results can be explained by the fact that all these genes belong to the same pathway.

At last, we treated cells with pifithrin-α (PFT), a TP53 inhibitor (74). We found no significant effect of PFT on human iPSC survival or editing efficiency (Supplementary Figure S6D), which is in contrast to a recent report on enhanced effects of blocking TP53 on genome editing (64). This may be due to insufficient effects of pifithrin-α on human PSCs (75).

### Further increase in editing efficiency by selecting against cells with low-level BCL-XL

**Figure 3.**
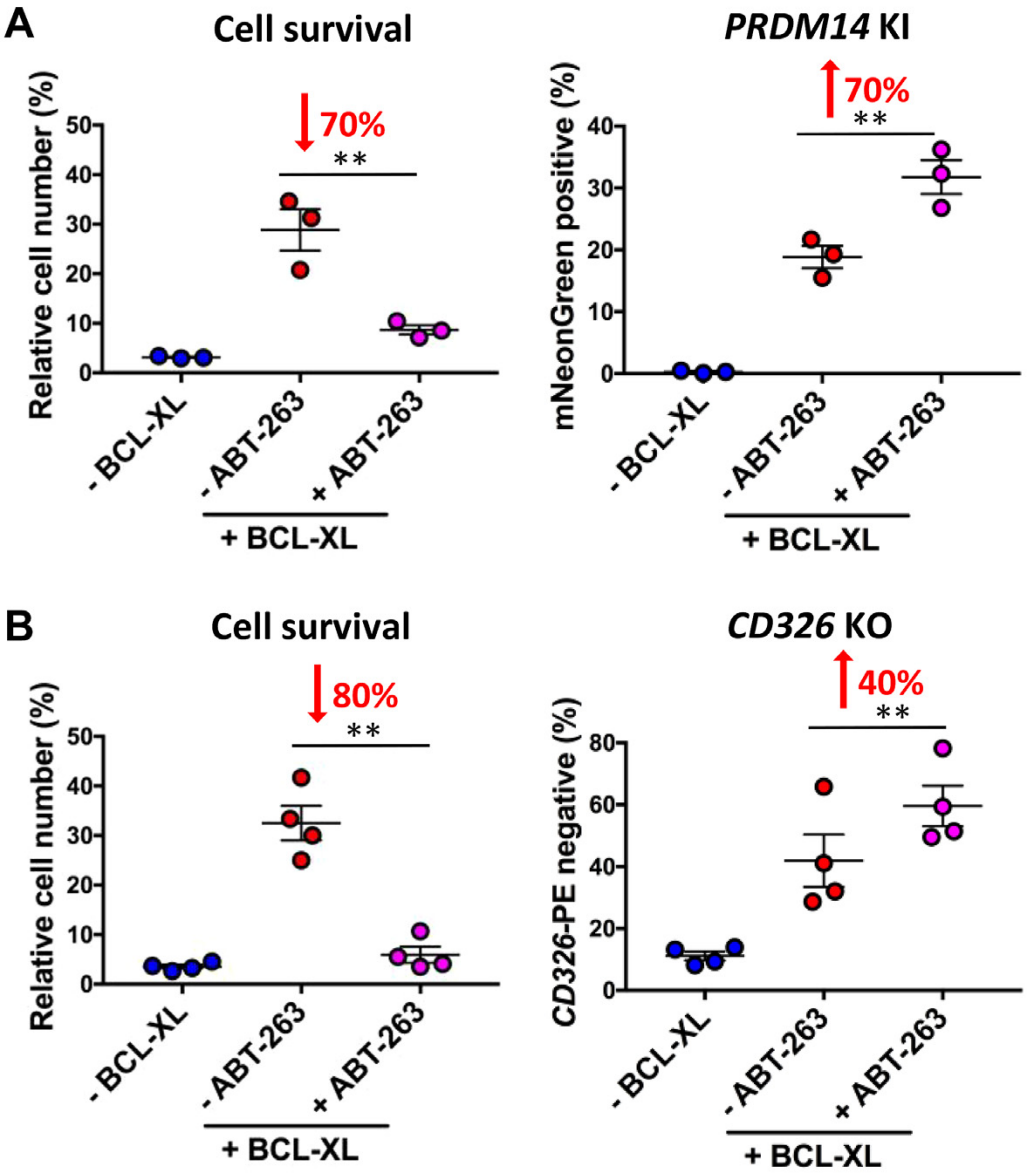
BCL inhibition further increases editing efficiency in BCL-XL transfected iPSCs. (**A**) Combined treatment of ABT-263, a BCL inhibitor, compromises survival yet improves editing. Treatment of ABT-263 (1 μM, 8–24 h) compromises cell survival by 70% (left, *n* = 3), while further improving *PRDM14* KI efficiency (right, *n* = 3). (**B**) Treatment of ABT-263 (1 μM, 8–24 h) compromises cell survival by 80% (left, *n* = 4), while further improving *CD326* KO efficiency (right, *n* = 4); **P < 0.01.

Having demonstrated that iPSCs with a high copy number of CRISPR plasmids have enhanced editing efficiency (Supplementary Figure S4), we hypothesize that further enriching these cells would increase the editing efficiency in a bulk population. To preferentially deplete the cells transfected with low copies of plasmids, we treated the cells with ABT-263 (Navitoclax), a potent inhibitor of BCL-XL, BCL2, and BCL-W (42). As expected, adding ABT-263 (0.2 or 0.5 μM) right after electroporation when no exogenous BCL-XL was expressed, led to a sharp reduction in cell survival and no significant improvement in editing efficiency (Figure 3A). In contrast, when ABT-263 was administered from 8 hours, when robust BCL-XL has been expressed (Supplementary Figure S3), until 24 hours, a dose-dependent gradual decrease in cell survival and increase in editing efficiency was observed. With 1 μM ABT-263, there was 50–70% reduction in cell survival, and 70 and 40% improvement in KI and KO editing efficiency, respectively (Figure 3A and B; Supplementary Figure S9). Consistent with increased HDR, deep sequencing of these samples showed that ABT-263 treatment also increased NHEJ indel frequencies by 50–70% (Supplementary Tables S4A,B and S5A,B). These results indicate that the use of BCL-XL in iPSC genome editing allows for cell selection and enrichment of successfully edited cells by administration of BCL inhibitors.

### Confirmation of editing efficiency by deep sequencing

**Figure 4.**
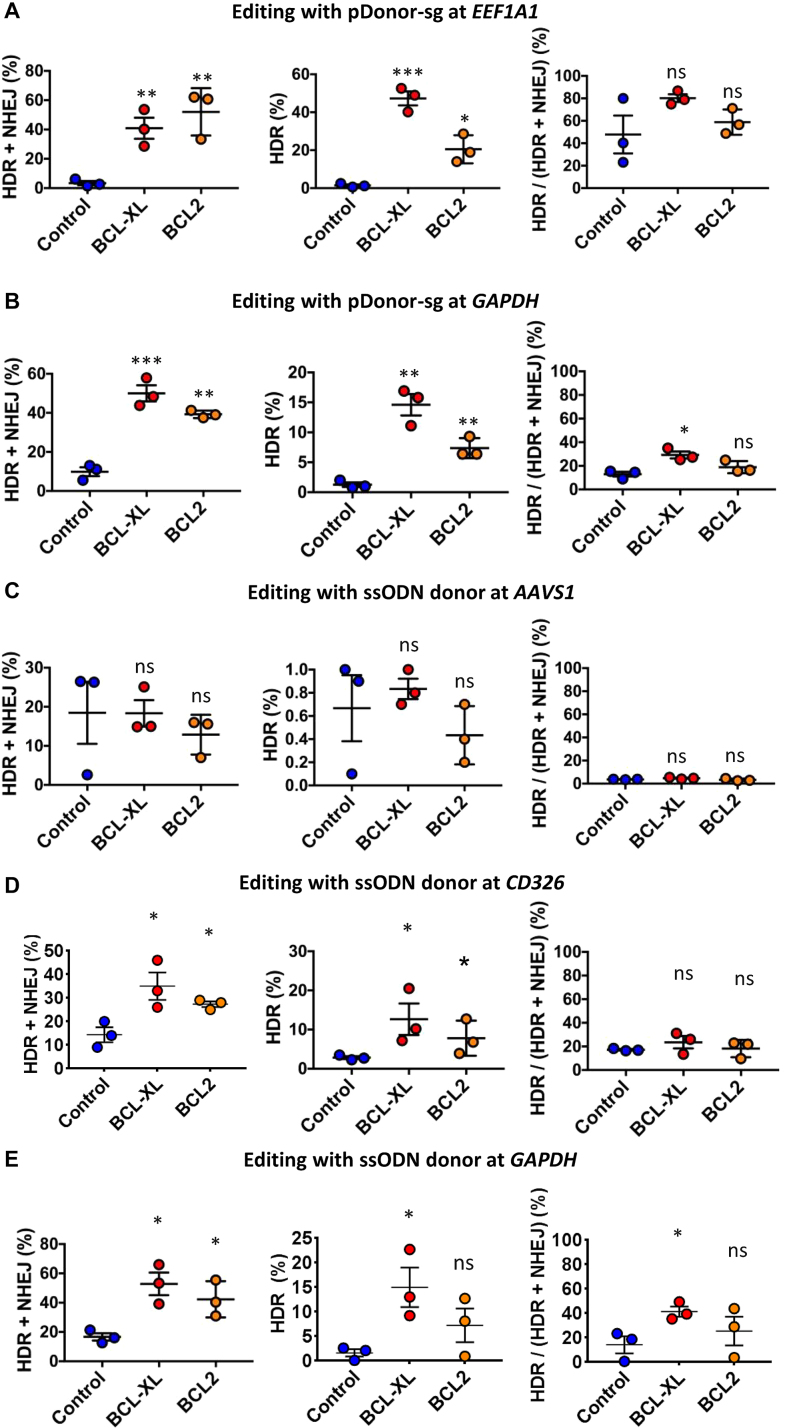
HDR/NHEJ editing efficiencies with double cut donors or ssODN donors as determined by high-throughput sequencing. (**A** and **B**) Indels (HDR + NHEJ), HDR efficiency, and HDR percentage in all edited cells at *EEF1A1* (A) and *GAPDH* (B) with pDonor-sg (double cut donor) in iPSCs; HDR percentage was calculated by the ratio of HDR to HDR plus NHEJ. (**C–E**) Indels (HDR + NHEJ), HDR efficiency, and HDR percentage in all edited cells at *AAVS1* (C), *CD326* (D), and *GAPDH* (E) using ssODN donors (single-stranded oligo DNAs) in iPSCs. The nucleofections were conducted without (Control) or with BCL-XL/BCL2; *n* = 3; *P < 0.05, **P < 0.01, ***P < 0.001; ns = not significant.

In the above studies, we designed donor templates to insert a fluorescent protein expression cassette (1–2 kb) by HDR, followed by quantitating HDR efficiencies by FACS. To further validate these results, we designed pDonors to insert a short fragment of 6–12 bp at *EEF1A1* and *GAPDH*, allowing assessment of both NHEJ and HDR editing by deep sequencing. We expect that BCL-XL also increases HDR editing efficiency when using a template other than plasmids. As such, we decided to edit *AAVS1, CD326*, and *GAPDH* loci using ssODNs. We observed higher levels of NHEJ and HDR editing at *EEF1A1, GAPDH*, and *CD326* loci than those at *AAVS1* (Table 1). At the three loci with higher editing levels, both BCL-XL and BCL2 significantly increased NHEJ and HDR editing efficiencies (Figure 4). Consistent with earlier results (Figure 2), BCL-XL tended to have a greater effect. We also analyzed the ratio of HDR to total indels and found that BCL-XL appeared to increase the proportion of HDR. However, whether BCL-XL affects the damage repair pathway choice is inconclusive since a higher HDR/(HDR+NHEJ) ratio is also associated with higher total indels. Taken together, these data demonstrate that overexpressing BCL-XL is a universal approach for increasing editing efficiency in human PSCs.

**Table 1.** Representative patterns of HDR and NHEJ editing with pDonor-sg or ssODN at different loci

HDR and NHEJ at *EEF1A1* with pDonor-sg			
Total reads = 33,041	Type	Indel (bp)	Read %
ATACAACTGAACAGTAC|TTTGGG	WT	0	37.9%
ATACAACTGAACAGgtttaaacgcgtGGG	**HDR**	−6, +12	49.0%
ATACAACTGAACAGTACcTTTGGG	NHEJ	+1	0.8%
ATACAACTGAACAGTACaTTTGGG	NHEJ	+1	0.8%
ATACAACTGAACA———G	NHEJ	−9	0.7%
ATACAACTGAACAGTAC-TTGGG	NHEJ	−1	0.6%
ATACAACTGAACAGTACttTTTGGG	NHEJ	+2	0.5%
ATACAACTGAACAGTAC–TGGG	NHEJ	−2	0.5%
ATACAACTGAACAGTAC—GGG	NHEJ	−3	0.3%
ATACAACTGAACAGTA-TTTGGG	NHEJ	−1	0.3%
ATACAACTGAACAG—-TTGGG	NHEJ	−4	0.3%
ATACAACTGAACAGTACtTTTGGG	NHEJ	+1	0.3%
HDR and NHEJ at *GAPDH* with pDonor-sg			
Total reads = 139,341	Type	Indel (bp)	Read %
GACTGGCTCTTAAAAAG|TGCAGG	WT	0	42.3%
GACTGGCTCTTgtttaaacgcgtTGCAGG	**HDR**	−6, +12	15.8%
GACTGGCTCTTAA—-TGCAGG	NHEJ	−4	3.1%
GACTGGC—————-	NHEJ	−23	2.8%
GACTGGCTCTTA—–TGCAGG	NHEJ	−5	1.9%
GACTGGCTCTTAAAA—–AGG	NHEJ	−5	1.7%
GACTGGCTCTTAAAAA–GCAGG	NHEJ	-2	1.7%
GACTGGCTCTTAAAA–TGCAGG	NHEJ	−2	1.6%
GACTGGCTCTTAAAAA-TGCAGG	NHEJ	−1	0.8%
GACTGGCTCTTAAAAA——G	NHEJ	−6	0.8%
GACTGGCTCTTAAAAAGtTGCAGG	NHEJ	+1	0.7%
GACTGGCTC————–	NHEJ	−23	0.7%
HDR and NHEJ at *AAVS1* with ssODN donor			
Total reads = 33,631	Type	Indel (bp)	Read %
GGGGCCACTAGGGACAG|GATTGG	WT	0	74.9%
GGGGCCACTAGGGACAGacgcgtGATTGG	**HDR**	+6	1.0%
GGGGCCACTAGGGACA-GATTGG	NHEJ	−1	6.4%
GGGGCCACTAGGG—–ATTGG	NHEJ	−5	2.8%
GGGGCCACTAGGGACAG——	NHEJ	−12	2.1%
GGGGCCACTAGGGAC-GGATTGG	NHEJ	−1	1.4%
GGGGCCACTAGGGAC–GATTGG	NHEJ	−2	0.9%
GGGGCCACTAGGGACAGgGATTGG	NHEJ	+1	0.8%
GGGGCCACTA———–GG	NHEJ	−11	0.5%
GGGGCCACTAG———-GG	NHEJ	−10	0.4%
GGGGCCACTAGGGACAGcagGATTGG	NHEJ	+3	0.4%
GGGGCCACTAGGGA–GGATTGG	NHEJ	−2	0.3%
HDR and NHEJ at *CD326* with ssODN donor			
Total reads = 57,018	Type	Indel (bp)	Read %
GTTCGGGCTTCTGCTTG|CCGCGG	WT	0	46.7%
GTTCGGGCTTCTGCTTGacgcgtCCGCGG	**HDR**	+6	7.2%
GTTCGGGCTTCTGCTT-CCGCGG	NHEJ	−1	11.0%
GTTCGGGCTTCTGCTTgGCCGCGG	NHEJ	+1	3.1%
GTTCGGGCTTCTGCTT——-	NHEJ	−19	1.3%
GTTCGGGCTTCTGCTTG-CGCGG	NHEJ	−1	1.2%
GTTCGGGCTTCTG—-CCGCGG	NHEJ	−4	1.1%
GTTCGGGCTT——-CCGCGG	NHEJ	−7	1.1%
GTTCGGGCTTCTGCT–CCGCGG	NHEJ	−2	0.9%
GTTCG—————-GG	NHEJ	−16	0.8%
GTTCGGGCTTCTGC———	NHEJ	−25	0.8%
GTTCGGGCTTCTGCTT——G	NHEJ	−6	0.8%

### Off-target analysis and digital karyotyping demonstrate safety of BCL-XL based editing strategy

One concern of CRISPR–Cas9 mediated gene editing is off-target effects, and high levels of the Cas9–sgRNA complex may increase chances of non-specific cleavage (20). In our strategy, BCL-XL selects for iPSCs with high copy numbers of editing plasmids, in particular with the addition of the BCL inhibitor ABT-263. This raised a concern of heightened off-target cleavage. To address this possibility, we amplified target DNA sequences in *PRDM14*- or *CTNNB11*-edited iPSCs by PCR, followed by Illumina paired-end sequencing (150 bp × 2). Putative off-target sites were predicted by the COSMID tool (54). We also examined on-target cleavage, which serves as a positive control for data analysis. Deep sequencing showed ∼20% indel efficiency at the *PRDM14* on-target locus and ∼30% indel efficiency at *CTNNB1* (Supplementary Table S4A and B). Meanwhile, in the ten edited iPSC lines, no indels were detected in the top 10–12 high-scoring off-target loci for *PRDM14* and *CTNNB1* (Supplementary Table S5A and B). Even when ABT-263 was used to further enrich edited iPSCs, off-target cleavage was undetectable at both sites (Supplementary Table S5A and B).

**Figure 5.**
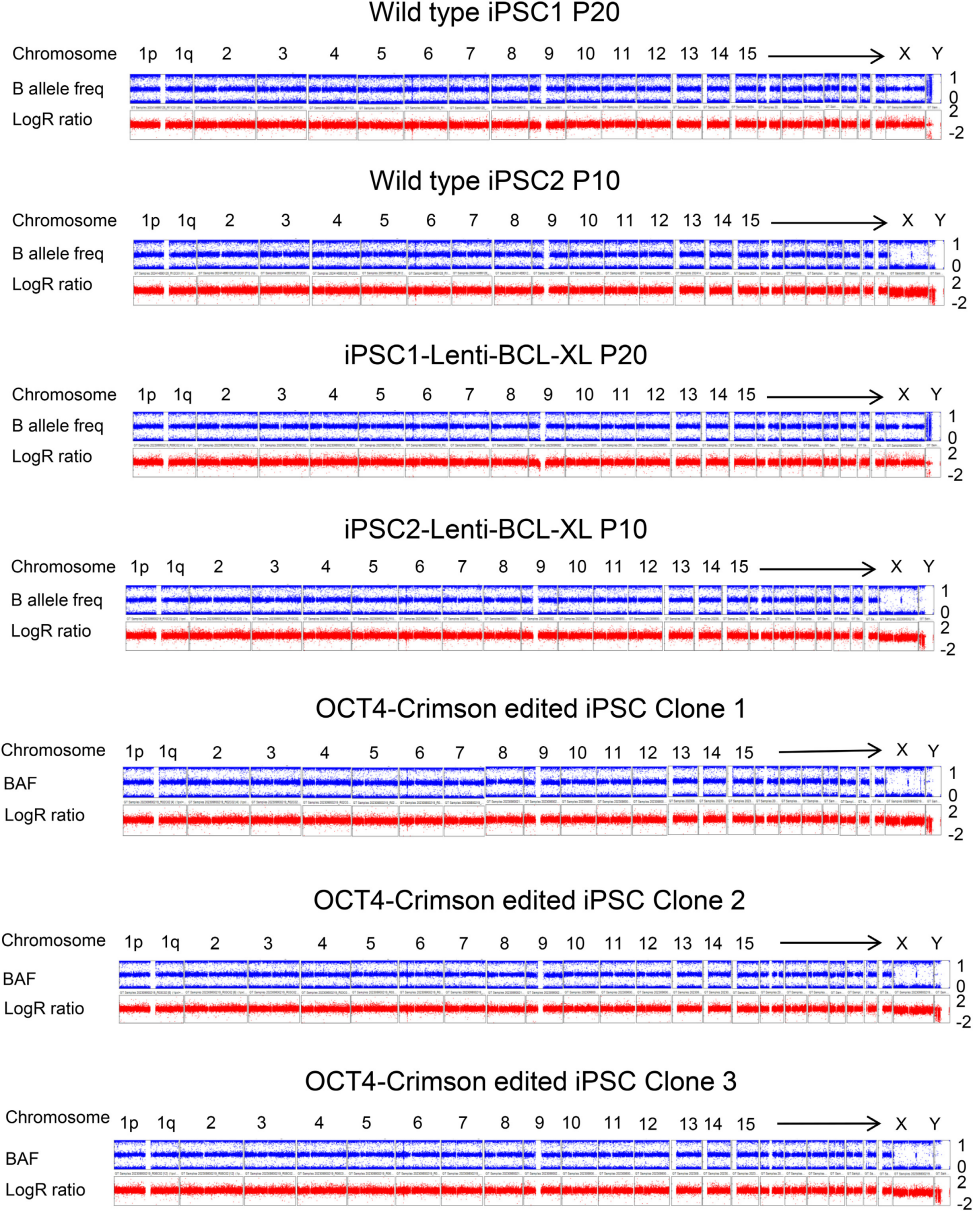
Digital karyotyping analysis of iPSCs. iPSC lines transduced with or without lenti BCL-XL were cultured for 10 or 20 passages before analysis. Three edited clones were also analyzed to identify potential chromosome abnormalities. All the clones we analyzed show normal karyotypes.

Multiple reports show that BCL-XL is associated with chromosome 20q11.21 amplification (76–78). Furthermore, 20q11.21 gain is also observed in human cancers (79). These reports raise safety concerns on the use of BCL-XL for genome editing. To explore whether BCL-XL transfection affects chromosome stability, we conducted digital karyotyping using an Illumina HumanCoreExome BeadChip, which provides greater resolution (100 kb) than G-banded karyotyping (∼5 Mb) (39,58,80). Analysis of two iPSC lines and three edited clones (with BCL-XL transfection) did not identify any abnormalities (Figure 5). To further assess the effects of BCL-XL on genome stability, we transduced iPSCs for long-term, stable BCL-XL expression. After 10–20 culture passages, these lines still did not show any appreciable changes in karyotype (Figure 5). These data demonstrate that BCL-XL overexpression *per se*, especially transiently, does not induce chromosome abnormality, which is consistent with an earlier report (36). In comparison, we also examined Jurkat, K562, and HEK293T cancer cell lines, and observed massive abnormalities. In particular, there were multiple monosomies and trisomies in K562 and HEK293T cells (Supplementary Figure S10). The results also validate the reliability of digital karyotyping.

### Rapid high-level knockin or knockout by dual or biallelic editing

The BCL-XL based approach has been routinely used in our lab to achieve efficient KI or KO in 20–50% of the bulk population of human iPSCs without any selection. However, single cell cloning is still necessary for downstream applications of edited iPSCs, such as directed differentiation. We reasoned that an editing efficiency >90% would largely eliminate the need for single cell selection and expansion, which is laborious and can take an additional 1–2 months to accomplish. To this end, we tested several selection strategies to enrich edited cells.

**Figure 6.**
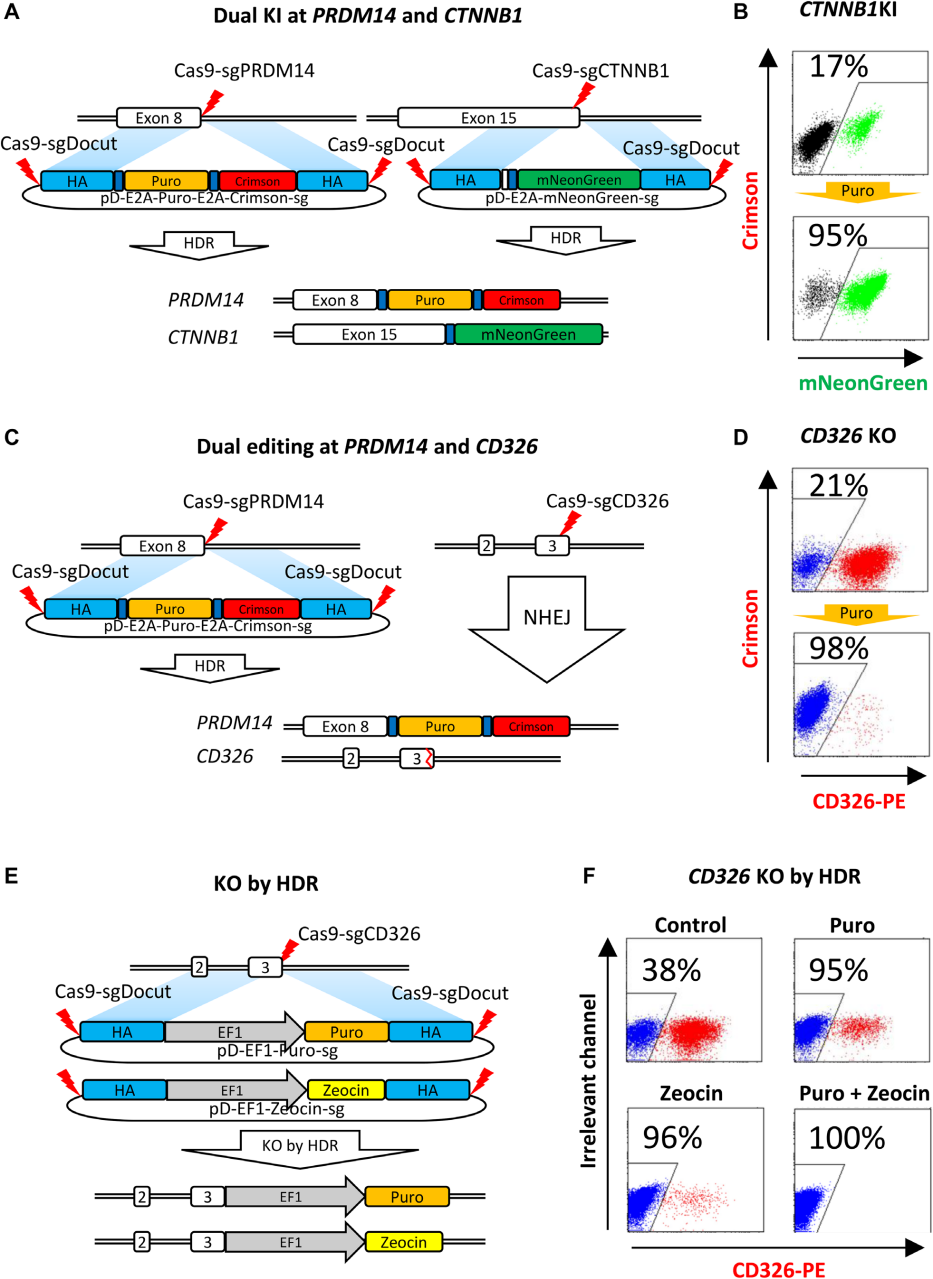
Rapid high-level KI or KO by dual or biallelic editing. (**A**) HDR-mediated dual KI at *PRDM14* and *CTNNB1* in iPSCs. CRISPR plasmids together with BCL-XL plasmid were electroporated into iPSCs. HDR KI of the E2A-Puro-E2A-Crimson cassette at *PRDM14* allows for puromycin selection to enrich iPSCs with HDR editing at *CTNNB1*. (**B**) Co-enrichment of *PRDM14* and *CTNNB1* HDR-edited iPSCs by single selection. Puromycin (1 μg/ml) was added 2 days after electroporation for selection. KI efficiency (mNeonGreen-positive) at *CTNNB1* was determined by FACS. (**C**) Dual editing at *PRDM14* by HDR and *CD326* by NHEJ. Similar procedure with (A) was carried out. (**D**) Co-enrichment of *PRDM14-* and *CD326-*edited iPSCs by single selection. Puromycin (1 μg/ml) was added 2 days after electroporation for selection. KO efficiency at *CD326* (CD326-PE-negative) was determined by FACS. (**E**) Schematic for gene KO by biallelic HDR insertion of section cassettes. Two double cut HDR donors (pD-HDR-CD326-EF1-Puro-sg and pD-HDR-CD326-EF1-Zeo-sg) were designed to insert Puro or Zeocin resistance genes at *CD326*, leading to biallelic disruption of the open reading frame. All CRISPR plasmids together with BCL-XL plasmid were electroporated into iPSCs, followed with single or double selection. (**F**) Complete KO in 100% iPSCs in a single step by double selection. Single selection by puromycin (1 μg/ml) or zeocin (100 μg/ml), or double selection by both were carried out 2 days after electroporation. KO efficiency at *CD326* (CD326-PE-negative) was determined by FACS 10 days later.

In the above studies, we edited a single locus each time. This approach can be further used to edit multiple loci simultaneously by using one locus inserted with a selective cassette to enrich iPSCs with successful editing at another. To test this concept, we designed a double cut donor with the Puro resistance gene, targeting the *PRDM14* locus. We carried out the experiments to target *PRDM14* and *CTNNB1* simultaneously, followed by puromycin selection (Figure 6A). As expected, HDR KI efficiency at *CTNNB1* increased from 17 to 95% after puromycin selection (Figure 6B). Similarly, we edited *PRDM14* and *CD326* simultaneously (Figure 6C). After selection, *CD326* KO cells were enriched from 21 to 98% (Figure 6D). These data demonstrate that a dual editing strategy can be readily used to increase HDR or NHEJ editing efficiency to over 95% in bulk iPSCs.

In demanding applications such as knocking out a gene with a growth advantage, 95% KO in bulk cells is insufficient. In this scenario, we reasoned that an insertion at two alleles with two different selection markers would disrupt the open reading frame and allow selection against all of the unedited cells. To test this concept, we designed two donors pD-CD326-EF1-Puro-sg and pD-CD326-EF1-Zeo-sg, with 600 bp HAs to guide the HDR insertion at *CD326* (Figure 6E). One week after electroporation, antibody staining and FACS analysis showed a KO efficiency of 38%. Selection with either puromycin or zeocin increased KO efficiency to 95–96%, while double selection with both puromycin and zeocin led to 100% KO (Figure 6F). These data demonstrate that knockout in virtually 100% cells can be achieved by biallelic knockin of selective cassettes.

### Differential effects of BCL2 family on editing of multiple cell lines

BCL-XL strikingly enhances the editing efficiency in human iPSCs, largely by increasing the survival of plasmid-electroporated cells by 10- to 20-fold. To determine whether BCL family can also facilitate gene editing in other cell lines, we tested the effects of BCL-XL, BCL2, and MCL1 in mouse ESCs, HEK293T (human embryonic kidney cells), K562 (human erythroleukemia cells), and Jurkat (T-cell leukemia) cells. In all of these lines, electroporation with BCL did not increase cell survival. To facilitate the detection of HDR editing, we designed a promoterless pDonor-mNeonGreen to target the intron before the stop codon-located exon of universally expressed genes *EEF1A1* and *GAPDH*. In mouse ESCs, two sgRNAs were designed to target the *EEF1A1* intron. BCL-XL increased HDR editing in all the mouse ESC experiments by 10–40% (*P* < 0.05), whereas BCL2 and MCL1 were less effective (Supplementary Figure S11A). In K562 cells, BCL-XL and MCL1 had no obvious benefits, while surprisingly, BCL2 significantly decreased HDR efficiency at both *EEF1A1* and *GAPDH* (Supplementary Figure S11B). In HEK293T cells, BCL-XL and BCL2 significantly decreased HDR efficiency at both *EEF1A1* and *GAPDH*, while MCL1 had no obvious effect (Supplementary Figure S11C). In Jurkat cells, BCL-XL and MCL1 showed a trend for enhancing editing efficiency, but the differences were statistically insignificant (Supplementary Figure S11D). Taken together, these data indicate that BCL-XL increases HDR efficiency in mouse ESCs and BCL2 members have differential effects on gene editing in different cell lines.

## DISCUSSION

Here, we report a simple approach to greatly improve cell survival and editing efficiency in human iPSCs by transiently overexpressing BCL-XL. We show that massive cell death (>95%) is a major barrier that limits editing efficiency in iPSCs after plasmid electroporation, and that BCL-XL improves cell survival by 10- to 20-fold when examined 1–2 days after electroporation), leading to a 20- to 100-fold and 5-fold increase in HDR-mediated KI and NHEJ-mediated KO efficiency, respectively. Using simple selection strategies, 95% HDR and100% NHEJ efficiency can be achieved. This efficiency renders it seemingly unnecessary to conduct single cell cloning, which has been routinely used in iPSC studies that do not require isogenic clones. These methods should have important applications in both iPSC studies and embryo manipulations.

Mouse ESCs predominantly use the HDR pathway to repair dsDNA damage, relative to somatic cells (81). Many studies found, however, that HDR efficiency in human PSCs is considerably lower than in other cell types (21–23). This puzzle has recently been solved, partly due to two reports published during revision of our work (63,64). Human PSCs are primed to undergo rapid death due to the balance of pro-apoptotic and anti-apoptotic BCL2 family proteins in the mitochondria (71,82). In particular, human PSCs undergo more rapid death after DNA damage-mediated activation of TP53 signaling than differentiated cells (82). Similarly, human PSCs are extremely sensitive to DSBs induced by Cas9–sgRNA, which activates TP53 signaling, leading to cell cycle arrest and apoptosis (64). In contrast to using lentiviral vectors for editing, we used plasmids, which have additional confounding factors such as physical damage of plasma membranes and plasmid electroporation. In our editing system, electroporation-induced damage of the plasma membrane leads to the death of about 50% of cells, while plasmid transfection induces 80% of cell death. Cytoplasmic DNA often triggers an innate immune response in most types of cells (83,84). However, of interest, we did not observe functional pathways that sense and respond to transfected plasmids (Supplementary Figure S7C). As such, other unknown mechanisms might have contributed to plasmid-mediated PSC death. Taken together, the cell death in our system is largely induced by plasmid electroporation and TP53 activation.

We demonstrate here that iPSC survival and gene editing efficiency can be greatly improved by overexpressing BCL-XL. Interestingly, transient BCL-XL overexpression showed lower cell survival but higher gene editing efficiency than stable BCL-XL overexpression in iPSCs. This can be explained by the survival advantage of cells transfected with more copies of BCL-XL and editing components, as evident by ∼5-fold higher expression of Cas9–sgRNA in BCL-XL treated cells. The BCL inhibitor ABT-263 confers an additional selective pressure for cells with high copy numbers of BCL-XL and editing plasmids, leading to 40–70% increase in KO and KI efficiency.

We can reproducibly achieve up to 50% editing efficiency in human iPSCs without any selection, but this may vary from one locus to another. Using a dual editing approach followed by selection, over 95% HDR KI or NHEJ KO efficiency can be achieved. Moreover, virtually 100% of HDR-mediated KO efficiency was achieved by biallelic KI of both puromycin and zeocin resistance gene expression cassettes driven by a strong promoter, followed by double selection. These simple strategies abrogate the need for single cell cloning, which is tedious and takes 1–2 months to procure sufficient amounts of edited cells for functional studies. In contrast, our new approach entails minimal hands-on time and large quantities of edited iPSCs can be obtained in 1–2 weeks. The widespread use of these simple methods will undoubtedly expedite basic or translational research that may benefit from genome editing of human iPSCs, such as functional genomics, disease modeling, etc.

This is not the first report showing that expression of BCL2 family proteins protects PSC death. Overexpression of BCL2 or BCL-XL protects dissociation-induced cell death (35,36). However, we extended these findings and found that BCL-XL can partly protect electroporation and CRISPR–Cas9 engineering-induced DNA damage stress. We also found that BCL-XL is the best factor to achieve these goals. These findings are reminiscent of our previous studies on reprogramming. It has puzzled us that BCL-XL increases reprogramming efficiency by ∼3-fold in a lentiviral transduction system, while increasing efficiency ∼10-fold in the electroporation system (37). This discrepancy can be explained by the fact that (i) lentiviral vector transduction-mediated reprogramming, similar to editing (but less so), also activates the TP53 pathway (85) and (ii) cells reprogrammed by electroporation also suffer physical damage. These data support the conclusion that BCL-XL protects cell death induced by both p53 activation and other stressors like physical damage.

A recent report indicates that TP53 inhibition increases HDR efficiency by approximately 10-fold in human PSCs (64). However, strategies targeting TP53 may increase genetic instability and tumorigenic potential. Long-term TP53 deficiency induces chromosome abnormality and cancer, most likely because TP53 inhibition induces premature cell cycle progression, preventing the appropriate repair of damaged DNA. In our system, the TP53 pathway is intact, as evidenced by upregulation of dozens of TP53 target genes (Supplementary Figure S7). BCL-XL only inhibits apoptosis and does not affect cell cycle arrest, as evidened by ∼5-fold increase in CDKN1A, also known as p21, 8 hours after nucleofection of BCL-XL and editing plasmids (Supplementary Figure S7E). BCL-XL overexpression maintains survival during cell cycle arrest, offering more time for cells to recover. As such, it is tempting to speculate that our approach is both safer and more powerful than blocking TP53.

Our BCL-XL based editing approach is safe as evidenced by the absence of off-target cleavage and chromosome abnormality. BCL-XL confers a survival advantage on iPSCs transfected with high copies of editing plasmids, but this did not lead to high levels of unspecific cleavage. This may be explained by considerably lower amounts of Cas9–sgRNA mRNA in iPSCs relative to the levels in cancer cells like HEK293T (86). Although BCL-XL amplification is found in many PSC lines (76–78), this can be explained by the fact that increased BCL-XL expression offers a proliferative advantage when culture conditions are suboptimal. In support of this argument, our digital karyotyping data presented here, together with early reports (36), demonstrate that long-term overexpression of BCL-XL does not lead to any obvious abnormalities. In addition, BCL-XL overexpression is transient in our system, and high-level expression is observed only 8–48 hours after nucleofection. After one week, BCL-XL is almost undetectable in edited cells. This should further mitigate the safety concerns of BCL-XL.

The BCL2 family is the central regulator that arbitrates the life-and-death decision. This family is classified into three subgroups: (1) anti-apoptotic factors such as BCL-XL, BCL2, and MCL1, which inhibit effectors from forming mitochondrial outer membrane permeabilization (MOMP), (2) pro-apoptotic BH3-only proteins, and (3) effectors such as BAK1 and BAX that mediate MOMP. The interactions between the anti-apoptotic repertoire, the pro-apoptotic BH3-only proteins, and the effector proteins decide the fate of the cell: survival or commitment to apoptosis (41). As such, overexpression of anti-apoptotic factors has been commonly used to increase the survival of cells in diverse settings. However, each factor may have differential protective effects in different scenarios, and we find that BCL-XL is the most potent in the iPSC editing.

The impressive effects of BCL-XL can only be partly replaced by the overexpression of other anti-apoptotic genes such as *BCL2*, or the KO of pro-apoptotic genes such as *BAX* and *BBC3*, but none of these are as effective as BCL-XL. In addition, inhibiting caspase or TP53 with small molecules showed no obvious effects on the improvement of cell survival and editing efficiency. The superior effects of BCL-XL over BCL2 and MCL1 are reminiscent of our previous finding that BCL-XL is more potent than BCL2 or MCL1 in enhancing reprogramming of iPSCs from adult human peripheral blood cells (38,60).

We found that BCL-XL also significantly increases HDR editing in mouse ESCs, although it did not increase cell survival after electroporation. As such, it is tempting to speculate that BCL-XL plasmids or RNA may enhance HDR editing in PSCs or early embryos from mice, rats, pigs, non-human primates, etc. If confirmed we envision that, together with the double cut donor design (17), the success rates of creating compound animal models can be considerably increased (15,87).

We also observed differential effects of BCL-XL, BCL2, and MCL1 on HDR editing in K562, HEK293T, and Jurkat cells. In particular, BCL2 overexpression decreases the editing efficiency in K562 and HEK293T cells. Manipulating factors related to cell death may have unexpected effects on affecting genome editing efficiency in different types of cells.

In summary, BCL-XL has an impressive effect on improving cell survival and gene editing efficiency in human iPSCs, which strikingly simplifies the procedure for generating a relatively pure population of HDR KI or NHEJ KO edited cells. These simple and robust methods will find broad applications in disease modeling, generation of reporter lines, gene modification, and functional genomics.

## DATA AVAILABILITY

The RNA-seq data on human iPSCs after nucleofection with or without BCL-XL have been deposited in the SRA database under the accession number SRP151274. All the other data and results are available upon request.

## Supplementary Material

Supplementary Data

## References

[1] Thomson J.A., Itskovitz-Eldor J., Shapiro S.S., Waknitz M.A., Swiergiel J.J., Marshall V.S., Jones J.M. Embryonic stem cell lines derived from human blastocysts. Science. 1998; 282:1145–1147. 9804556 10.1126/science.282.5391.1145

[2] Yu J., Vodyanik M.A., Smuga-Otto K., Antosiewicz-Bourget J., Frane J.L., Tian S., Nie J., Jonsdottir G.A., Ruotti V., Stewart R. Induced pluripotent stem cell lines derived from human somatic cells. Science. 2007; 318:1917–1920. 18029452 10.1126/science.1151526

[3] Takahashi K., Tanabe K., Ohnuki M., Narita M., Ichisaka T., Tomoda K., Yamanaka S. Induction of pluripotent stem cells from adult human fibroblasts by defined factors. Cell. 2007; 131:861–872. 18035408 10.1016/j.cell.2007.11.019

[4] Zhang X.B. Cellular reprogramming of human peripheral blood cells. Genomics Proteomics Bioinformatics. 2013; 11:264–274. 24060839 10.1016/j.gpb.2013.09.001 PMC4357833

[5] Thomas K.R., Capecchi M.R. Site-directed mutagenesis by gene targeting in mouse embryo-derived stem cells. Cell. 1987; 51:503–512. 2822260 10.1016/0092-8674(87)90646-5

[6] San Filippo J., Sung P., Klein H. Mechanism of eukaryotic homologous recombination. Annu. Rev. Biochem. 2008; 77:229–257. 18275380 10.1146/annurev.biochem.77.061306.125255

[7] Capecchi M.R. Altering the genome by homologous recombination. Science. 1989; 244:1288–1292. 2660260 10.1126/science.2660260

[8] Urnov F.D., Rebar E.J., Holmes M.C., Zhang H.S., Gregory P.D. Genome editing with engineered zinc finger nucleases. Nat. Rev. Genet. 2010; 11:636–646. 20717154 10.1038/nrg2842

[9] Joung J.K., Sander J.D. TALENs: a widely applicable technology for targeted genome editing. Nat. Rev. Mol. Cell Biol. 2013; 14:49–55. 23169466 10.1038/nrm3486 PMC3547402

[10] Jinek M., Chylinski K., Fonfara I., Hauer M., Doudna J.A., Charpentier E. A programmable dual-RNA-guided DNA endonuclease in adaptive bacterial immunity. Science. 2012; 337:816–821. 22745249 10.1126/science.1225829 PMC6286148

[11] Cong L., Ran F.A., Cox D., Lin S., Barretto R., Habib N., Hsu P.D., Wu X., Jiang W., Marraffini L.A. Multiplex genome engineering using CRISPR/Cas systems. Science. 2013; 339:819–823. 23287718 10.1126/science.1231143 PMC3795411

[12] Mali P., Yang L., Esvelt K.M., Aach J., Guell M., DiCarlo J.E., Norville J.E., Church G.M. RNA-guided human genome engineering via Cas9. Science. 2013; 339:823–826. 23287722 10.1126/science.1232033 PMC3712628

[13] Makarova K.S., Wolf Y.I., Alkhnbashi O.S., Costa F., Shah S.A., Saunders S.J., Barrangou R., Brouns S.J., Charpentier E., Haft D.H. An updated evolutionary classification of CRISPR-Cas systems. Nat. Rev. Microbiol. 2015; 13:722–736. 26411297 10.1038/nrmicro3569 PMC5426118

[14] Lieber M.R. The mechanism of double-strand DNA break repair by the nonhomologous DNA end-joining pathway. Annu. Rev. Biochem. 2010; 79:181–211. 20192759 10.1146/annurev.biochem.052308.093131 PMC3079308

[15] Wang H., Yang H., Shivalila C.S., Dawlaty M.M., Cheng A.W., Zhang F., Jaenisch R. One-step generation of mice carrying mutations in multiple genes by CRISPR/Cas-mediated genome engineering. Cell. 2013; 153:910–918. 23643243 10.1016/j.cell.2013.04.025 PMC3969854

[16] Hsu P.D., Lander E.S., Zhang F. Development and applications of CRISPR-Cas9 for genome engineering. Cell. 2014; 157:1262–1278. 24906146 10.1016/j.cell.2014.05.010 PMC4343198

[17] Zhang J.P., Li X.L., Li G.H., Chen W., Arakaki C., Botimer G.D., Baylink D., Zhang L., Wen W., Fu Y.W. Efficient precise knockin with a double cut HDR donor after CRISPR/Cas9-mediated double-stranded DNA cleavage. Genome Biol. 2017; 18:35. 28219395 10.1186/s13059-017-1164-8 PMC5319046

[18] Yao X., Wang X., Hu X., Liu Z., Liu J., Zhou H., Shen X., Wei Y., Huang Z., Ying W. Homology-mediated end joining-based targeted integration using CRISPR/Cas9. Cell Res. 2017; 27:801–814. 28524166 10.1038/cr.2017.76 PMC5518881

[19] Chen X., Janssen J.M., Liu J., Maggio I., t Jong A.E.J., Mikkers H.M.M., Goncalves M. In trans paired nicking triggers seamless genome editing without double-stranded DNA cutting. Nat. Commun. 2017; 8:657. 28939824 10.1038/s41467-017-00687-1 PMC5610252

[20] Hsu P.D., Scott D.A., Weinstein J.A., Ran F.A., Konermann S., Agarwala V., Li Y., Fine E.J., Wu X., Shalem O. DNA targeting specificity of RNA-guided Cas9 nucleases. Nat. Biotechnol. 2013; 31:827–832. 23873081 10.1038/nbt.2647 PMC3969858

[21] Zou J., Maeder M.L., Mali P., Pruett-Miller S.M., Thibodeau-Beganny S., Chou B.K., Chen G., Ye Z., Park I.H., Daley G.Q. Gene targeting of a disease-related gene in human induced pluripotent stem and embryonic stem cells. Cell Stem Cell. 2009; 5:97–110. 19540188 10.1016/j.stem.2009.05.023 PMC2720132

[22] Soldner F., Laganiere J., Cheng A.W., Hockemeyer D., Gao Q., Alagappan R., Khurana V., Golbe L.I., Myers R.H., Lindquist S. Generation of isogenic pluripotent stem cells differing exclusively at two early onset Parkinson point mutations. Cell. 2011; 146:318–331. 21757228 10.1016/j.cell.2011.06.019 PMC3155290

[23] He X., Tan C., Wang F., Wang Y., Zhou R., Cui D., You W., Zhao H., Ren J., Feng B. Knock-in of large reporter genes in human cells via CRISPR/Cas9-induced homology-dependent and independent DNA repair. Nucleic Acids Res. 2016; 44:e85. 26850641 10.1093/nar/gkw064 PMC4872082

[24] Byrne S.M., Ortiz L., Mali P., Aach J., Church G.M. Multi-kilobase homozygous targeted gene replacement in human induced pluripotent stem cells. Nucleic Acids Res. 2015; 43:e21. 25414332 10.1093/nar/gku1246 PMC4330342

[25] Watanabe K., Ueno M., Kamiya D., Nishiyama A., Matsumura M., Wataya T., Takahashi J.B., Nishikawa S., Nishikawa S., Muguruma K. A ROCK inhibitor permits survival of dissociated human embryonic stem cells. Nat. Biotechnol. 2007; 25:681–686. 17529971 10.1038/nbt1310

[26] Ohgushi M., Matsumura M., Eiraku M., Murakami K., Aramaki T., Nishiyama A., Muguruma K., Nakano T., Suga H., Ueno M. Molecular pathway and cell state responsible for dissociation-induced apoptosis in human pluripotent stem cells. Cell Stem Cell. 2010; 7:225–239. 20682448 10.1016/j.stem.2010.06.018

[27] Chen G., Hou Z., Gulbranson D.R., Thomson J.A. Actin-myosin contractility is responsible for the reduced viability of dissociated human embryonic stem cells. Cell Stem Cell. 2010; 7:240–248. 20682449 10.1016/j.stem.2010.06.017 PMC2916864

[28] Zwaka T.P., Thomson J.A. Homologous recombination in human embryonic stem cells. Nat. Biotechnol. 2003; 21:319–321. 12577066 10.1038/nbt788

[29] Giudice A., Trounson A. Genetic modification of human embryonic stem cells for derivation of target cells. Cell Stem Cell. 2008; 2:422–433. 18462693 10.1016/j.stem.2008.04.003

[30] Hofmann F., Ohnimus H., Scheller C., Strupp W., Zimmermann U., Jassoy C. Electric field pulses can induce apoptosis. J. Membr. Biol. 1999; 169:103–109. 10341032 10.1007/s002329900522

[31] Pinero J., Lopez-Baena M., Ortiz T., Cortes F. Apoptotic and necrotic cell death are both induced by electroporation in HL60 human promyeloid leukaemia cells. Apoptosis. 1997; 2:330–336. 14646546 10.1023/a:1026497306006

[32] Stacey K.J., Ross I.L., Hume D.A. Electroporation and DNA-dependent cell death in murine macrophages. Immunol. Cell Biol. 1993; 71:75–85. 8486399 10.1038/icb.1993.8

[33] Shimokawa T., Okumura K., Ra C. DNA induces apoptosis in electroporated human promonocytic cell line U937. Biochem. Biophys. Res. Commun. 2000; 270:94–99. 10733910 10.1006/bbrc.2000.2388

[34] Van De Parre T.J., Martinet W., Schrijvers D.M., Herman A.G., De Meyer G.R. mRNA but not plasmid DNA is efficiently transfected in murine J774A.1 macrophages. Biochem. Biophys. Res. Commun. 2005; 327:356–360. 15629470 10.1016/j.bbrc.2004.12.027

[35] Bai H., Chen K., Gao Y.X., Arzigian M., Xie Y.L., Malcosky C., Yang Y.G., Wu W.S., Wang Z.Z. Bcl-xL enhances single-cell survival and expansion of human embryonic stem cells without affecting self-renewal. Stem Cell Res. 2012; 8:26–37. 22099018 10.1016/j.scr.2011.08.002 PMC3222876

[36] Ardehali R., Inlay M.A., Ali S.R., Tang C., Drukker M., Weissman I.L. Overexpression of BCL2 enhances survival of human embryonic stem cells during stress and obviates the requirement for serum factors. Proc. Natl. Acad. Sci. U.S.A. 2011; 108:3282–3287. 21300885 10.1073/pnas.1019047108 PMC3044421

[37] Su R.-J., Baylink D.J., Neises A., Kiroyan J.B., Meng X., Payne K.J., Tschudy-Seney B., Duan Y., Appleby N., Kearns-Jonker M. Efficient generation of Integration-Free iPS cells from human adult peripheral blood using BCL-XL together with yamanaka factors. PLoS One. 2013; 8:e64496. 23704989 10.1371/journal.pone.0064496 PMC3660366

[38] Wen W., Zhang J.P., Xu J., Su R.J., Neises A., Ji G.Z., Yuan W., Cheng T., Zhang X.B. Enhanced generation of Integration-free iPSCs from human adult peripheral blood mononuclear cells with an optimal combination of episomal vectors. Stem Cell Rep. 2016; 6:873–884. 10.1016/j.stemcr.2016.04.005 PMC4911493 27161365

[39] Wen W., Cheng X., Fu Y., Meng F., Zhang J.P., Zhang L., Li X.L., Yang Z., Xu J., Zhang F. High-Level precise knockin of iPSCs by simultaneous reprogramming and genome editing of human peripheral blood mononuclear cells. Stem Cell Rep. 2018; 10:1821–1834. 10.1016/j.stemcr.2018.04.013 PMC5989814 29754960

[40] Vander Heiden M.G., Chandel N.S., Williamson E.K., Schumacker P.T., Thompson C.B. Bcl-xL regulates the membrane potential and volume homeostasis of mitochondria. Cell. 1997; 91:627–637. 9393856 10.1016/s0092-8674(00)80450-x

[41] Chipuk J.E., Moldoveanu T., Llambi F., Parsons M.J., Green D.R. The BCL-2 family reunion. Mol. Cell. 2010; 37:299–310. 20159550 10.1016/j.molcel.2010.01.025 PMC3222298

[42] Tse C., Shoemaker A.R., Adickes J., Anderson M.G., Chen J., Jin S., Johnson E.F., Marsh K.C., Mitten M.J., Nimmer P. ABT-263: a potent and orally bioavailable Bcl-2 family inhibitor. Cancer Res. 2008; 68:3421–3428. 18451170 10.1158/0008-5472.CAN-07-5836

[43] Zhang J.-P., Neises A., Cheng T., Zhang X.-B. Hematopoietic Differentiation of Human Pluripotent Stem Cells. 2015; Dordrecht Springer 103–116.

[44] Labun K., Montague T.G., Gagnon J.A., Thyme S.B., Valen E. CHOPCHOP v2: a web tool for the next generation of CRISPR genome engineering. Nucleic Acids Res. 2016; 44:W272–W276. 27185894 10.1093/nar/gkw398 PMC4987937

[45] Meng X., Neises A., Su R.-J., Payne K.J., Ritter L., Gridley D.S., Wang J., Sheng M., William Lau K.H., Baylink D.J. Efficient reprogramming of human cord blood CD34+ cells into induced pluripotent stem cells with OCT4 and SOX2 alone. Mol. Ther. 2012; 20:408–416. 22108860 10.1038/mt.2011.258 PMC3277237

[46] Su R.J., Neises A., Zhang X.B. Generation of iPS cells from human peripheral blood mononuclear cells using episomal vectors. Methods Mol. Biol. 2016; 1357:57–69. 25403468 10.1007/7651_2014_139

[47] Wen W., Zhang J.P., Chen W., Arakaki C., Li X., Baylink D., Botimer G.D., Xu J., Yuan W., Cheng T. Generation of Integration-free induced pluripotent stem cells from human peripheral blood mononuclear cells using episomal vectors. J. Vis. Exp. 2017; doi:10.3791/55091. 10.3791/55091 PMC5408725 28117800

[48] Rols M.P., Delteil C., Serin G., Teissie J. Temperature effects on electrotransfection of mammalian cells. Nucleic Acids Res. 1994; 22:540. 8127697 10.1093/nar/22.3.540 PMC523619

[49] Patro R., Duggal G., Love M.I., Irizarry R.A., Kingsford C. Salmon provides fast and bias-aware quantification of transcript expression. Nat. Methods. 2017; 14:417–419. 28263959 10.1038/nmeth.4197 PMC5600148

[50] Love M.I., Huber W., Anders S. Moderated estimation of fold change and dispersion for RNA-seq data with DESeq2. Genome Biol. 2014; 15:550. 25516281 10.1186/s13059-014-0550-8 PMC4302049

[51] Huang da W., Sherman B.T., Lempicki R.A. Systematic and integrative analysis of large gene lists using DAVID bioinformatics resources. Nat. Protoc. 2009; 4:44–57. 19131956 10.1038/nprot.2008.211

[52] Yang L., Guell M., Byrne S., Yang J.L., De Los Angeles A., Mali P., Aach J., Kim-Kiselak C., Briggs A.W., Rios X. Optimization of scarless human stem cell genome editing. Nucleic Acids Res. 2013; 41:9049–9061. 23907390 10.1093/nar/gkt555 PMC3799423

[53] Renaud J.B., Boix C., Charpentier M., De Cian A., Cochennec J., Duvernois-Berthet E., Perrouault L., Tesson L., Edouard J., Thinard R. Improved genome editing efficiency and flexibility using modified oligonucleotides with TALEN and CRISPR-Cas9 nucleases. Cell Rep. 2016; 14:2263–2272. 26923600 10.1016/j.celrep.2016.02.018

[54] Cradick T.J., Qiu P., Lee C.M., Fine E.J., Bao G. COSMID: a Web-based tool for identifying and validating CRISPR/Cas Off-target sites. Mol. Ther. Nucleic Acids. 2014; 3:e214. 25462530 10.1038/mtna.2014.64 PMC4272406

[55] Goecks J., Nekrutenko A., Taylor J., Galaxy T. Galaxy: a comprehensive approach for supporting accessible, reproducible, and transparent computational research in the life sciences. Genome Biol. 2010; 11:R86. 20738864 10.1186/gb-2010-11-8-r86 PMC2945788

[56] Park J., Lim K., Kim J.S., Bae S. Cas-analyzer: an online tool for assessing genome editing results using NGS data. Bioinformatics. 2017; 33:286–288. 27559154 10.1093/bioinformatics/btw561 PMC5254075

[57] Magoc T., Salzberg S.L. FLASH: fast length adjustment of short reads to improve genome assemblies. Bioinformatics. 2011; 27:2957–2963. 21903629 10.1093/bioinformatics/btr507 PMC3198573

[58] D’Antonio M., Woodruff G., Nathanson J.L., D’Antonio-Chronowska A., Arias A., Matsui H., Williams R., Herrera C., Reyna S.M., Yeo G.W. High-Throughput and Cost-Effective characterization of induced pluripotent stem cells. Stem Cell Rep. 2017; 8:1101–1111. 10.1016/j.stemcr.2017.03.011 PMC5390243 28410643

[59] Chen C., Smye S.W., Robinson M.P., Evans J.A. Membrane electroporation theories: a review. Med. Biol. Eng. Comput. 2006; 44:5–14. 16929916 10.1007/s11517-005-0020-2

[60] Su R.J., Baylink D.J., Neises A., Kiroyan J.B., Meng X., Payne K.J., Tschudy-Seney B., Duan Y., Appleby N., Kearns-Jonker M. Efficient generation of integration-free ips cells from human adult peripheral blood using BCL-XL together with Yamanaka factors. PLoS One. 2013; 8:e64496. 23704989 10.1371/journal.pone.0064496 PMC3660366

[61] Su R.J., Yang Y., Neises A., Payne K.J., Wang J., Viswanathan K., Wakeland E.K., Fang X., Zhang X.B. Few single nucleotide variations in exomes of human cord blood induced pluripotent stem cells. PLoS One. 2013; 8:e59908. 23573220 10.1371/journal.pone.0059908 PMC3613421

[62] Rothkamm K., Kruger I., Thompson L.H., Lobrich M. Pathways of DNA double-strand break repair during the mammalian cell cycle. Mol. Cell Biol. 2003; 23:5706–5715. 12897142 10.1128/MCB.23.16.5706-5715.2003 PMC166351

[63] Haapaniemi E., Botla S., Persson J., Schmierer B., Taipale J. CRISPR-Cas9 genome editing induces a p53-mediated DNA damage response. Nat. Med. 2018; 24:927–930. 29892067 10.1038/s41591-018-0049-z

[64] Ihry R.J., Worringer K.A., Salick M.R., Frias E., Ho D., Theriault K., Kommineni S., Chen J., Sondey M., Ye C. p53 inhibits CRISPR-Cas9 engineering in human pluripotent stem cells. Nat. Med. 2018; 24:939–946. 29892062 10.1038/s41591-018-0050-6

[65] Fischer M. Census and evaluation of p53 target genes. Oncogene. 2017; 36:3943–3956. 28288132 10.1038/onc.2016.502 PMC5511239

[66] Kastenhuber E.R., Lowe S.W. Putting p53 in context. Cell. 2017; 170:1062–1078. 28886379 10.1016/j.cell.2017.08.028 PMC5743327

[67] Gilbert B., Ahmad K., Roos J., Lehmann C., Chiba T., Ulrich-Ruckert S., Smeenk L., van Heeringen S., Maier T.J., Groner B. 5-Lipoxygenase is a direct p53 target gene in humans. Biochim. Biophys. Acta. 2015; 1849:1003–1016. 26070487 10.1016/j.bbagrm.2015.06.004

[68] Zhan Q. Gadd45a, a p53- and BRCA1-regulated stress protein, in cellular response to DNA damage. Mutat. Res. 2005; 569:133–143. 15603758 10.1016/j.mrfmmm.2004.06.055

[69] Zeron-Medina J., Wang X., Repapi E., Campbell M.R., Su D., Castro-Giner F., Davies B., Peterse E.F., Sacilotto N., Walker G.J. A polymorphic p53 response element in KIT ligand influences cancer risk and has undergone natural selection. Cell. 2013; 155:410–422. 24120139 10.1016/j.cell.2013.09.017 PMC4171736

[70] Michels J., Kepp O., Senovilla L., Lissa D., Castedo M., Kroemer G., Galluzzi L. Functions of BCL-X L at the interface between cell death and metabolism. Int. J. Cell Biol. 2013; 2013:705294. 23533418 10.1155/2013/705294 PMC3603586

[71] Dumitru R., Gama V., Fagan B.M., Bower J.J., Swahari V., Pevny L.H., Deshmukh M. Human embryonic stem cells have constitutively active Bax at the Golgi and are primed to undergo rapid apoptosis. Mol. Cell. 2012; 46:573–583. 22560721 10.1016/j.molcel.2012.04.002 PMC3372694

[72] Yu J., Zhang L. PUMA, a potent killer with or without p53. Oncogene. 2008; 27:S71–S83. 19641508 10.1038/onc.2009.45 PMC2860432

[73] Van Noorden C.J. The history of Z-VAD-FMK, a tool for understanding the significance of caspase inhibition. Acta Histochem. 2001; 103:241–251. 11482370 10.1078/0065-1281-00601

[74] Gudkov A.V., Komarova E.A. Prospective therapeutic applications of p53 inhibitors. Biochem. Biophys. Res. Commun. 2005; 331:726–736. 15865929 10.1016/j.bbrc.2005.03.153

[75] Qin H., Yu T., Qing T., Liu Y., Zhao Y., Cai J., Li J., Song Z., Qu X., Zhou P. Regulation of apoptosis and differentiation by p53 in human embryonic stem cells. J. Biol. Chem. 2007; 282:5842–5852. 17179143 10.1074/jbc.M610464200

[76] International Stem Cell, I. Amps K., Andrews P.W., Anyfantis G., Armstrong L., Avery S., Baharvand H., Baker J., Baker D., Munoz M.B. Screening ethnically diverse human embryonic stem cells identifies a chromosome 20 minimal amplicon conferring growth advantage. Nat. Biotechnol. 2011; 29:1132–1144. 22119741 10.1038/nbt.2051 PMC3454460

[77] Nguyen H.T., Geens M., Mertzanidou A., Jacobs K., Heirman C., Breckpot K., Spits C. Gain of 20q11.21 in human embryonic stem cells improves cell survival by increased expression of Bcl-xL. Mol. Hum. Reprod. 2014; 20:168–177. 24217388 10.1093/molehr/gat077

[78] Avery S., Hirst A.J., Baker D., Lim C.Y., Alagaratnam S., Skotheim R.I., Lothe R.A., Pera M.F., Colman A., Robson P. BCL-XL mediates the strong selective advantage of a 20q11.21 amplification commonly found in human embryonic stem cell cultures. Stem Cell Rep. 2013; 1:379–386. 10.1016/j.stemcr.2013.10.005 PMC3841249 24286026

[79] Beroukhim R., Mermel C.H., Porter D., Wei G., Raychaudhuri S., Donovan J., Barretina J., Boehm J.S., Dobson J., Urashima M. The landscape of somatic copy-number alteration across human cancers. Nature. 2010; 463:899–905. 20164920 10.1038/nature08822 PMC2826709

[80] Martins-Taylor K., Nisler B.S., Taapken S.M., Compton T., Crandall L., Montgomery K.D., Lalande M., Xu R.H. Recurrent copy number variations in human induced pluripotent stem cells. Nat. Biotechnol. 2011; 29:488–491. 21654665 10.1038/nbt.1890

[81] Tichy E.D., Pillai R., Deng L., Liang L., Tischfield J., Schwemberger S.J., Babcock G.F., Stambrook P.J. Mouse embryonic stem cells, but not somatic cells, predominantly use homologous recombination to repair double-strand DNA breaks. Stem Cells Dev. 2010; 19:1699–1711. 20446816 10.1089/scd.2010.0058 PMC3128311

[82] Liu J.C., Guan X., Ryan J.A., Rivera A.G., Mock C., Agrawal V., Letai A., Lerou P.H., Lahav G. High mitochondrial priming sensitizes hESCs to DNA-damage-induced apoptosis. Cell Stem Cell. 2013; 13:483–491. 23954752 10.1016/j.stem.2013.07.018 PMC4109647

[83] Roers A., Hiller B., Hornung V. Recognition of endogenous nucleic acids by the innate immune system. Immunity. 2016; 44:739–754. 27096317 10.1016/j.immuni.2016.04.002

[84] Muerdter F., Boryn L.M., Woodfin A.R., Neumayr C., Rath M., Zabidi M.A., Pagani M., Haberle V., Kazmar T., Catarino R.R. Resolving systematic errors in widely used enhancer activity assays in human cells. Nat. Methods. 2018; 15:141–149. 29256496 10.1038/nmeth.4534 PMC5793997

[85] Hong H., Takahashi K., Ichisaka T., Aoi T., Kanagawa O., Nakagawa M., Okita K., Yamanaka S. Suppression of induced pluripotent stem cell generation by the p53-p21 pathway. Nature. 2009; 460:1132–1135. 19668191 10.1038/nature08235 PMC2917235

[86] Zhang J.P., Li X.L., Neises A., Chen W., Hu L.P., Ji G.Z., Yu J.Y., Xu J., Yuan W.P., Cheng T. Different effects of sgRNA length on CRISPR-mediated gene knockout efficiency. Sci. Rep. 2016; 6:28566. 27338021 10.1038/srep28566 PMC4919781

[87] Yao X., Liu Z., Wang X., Wang Y., Nie Y.H., Lai L., Sun R., Shi L., Sun Q., Yang H. Generation of knock-in cynomolgus monkey via CRISPR/Cas9 editing. Cell Res. 2018; 28:379–382. 29327726 10.1038/cr.2018.9 PMC5835779

