# Supplementary material for: Highly efficient genome editing via CRISPR–Cas9 in human pluripotent stem cells is achieved by transient BCL-XL overexpression
Source: Nucleic Acids Res. 2018 Sep 20;46(19):10195–215. doi: 10.1093/nar/gky804 (PMC6212847; doi:10.1093/nar/gky804)
Supplement: Supplementary Data [file gky804_supplemental_files.zip › Genome Editing in iPSC NAR Supplementary Info 7-24-2018.pptx]

## Slide 1
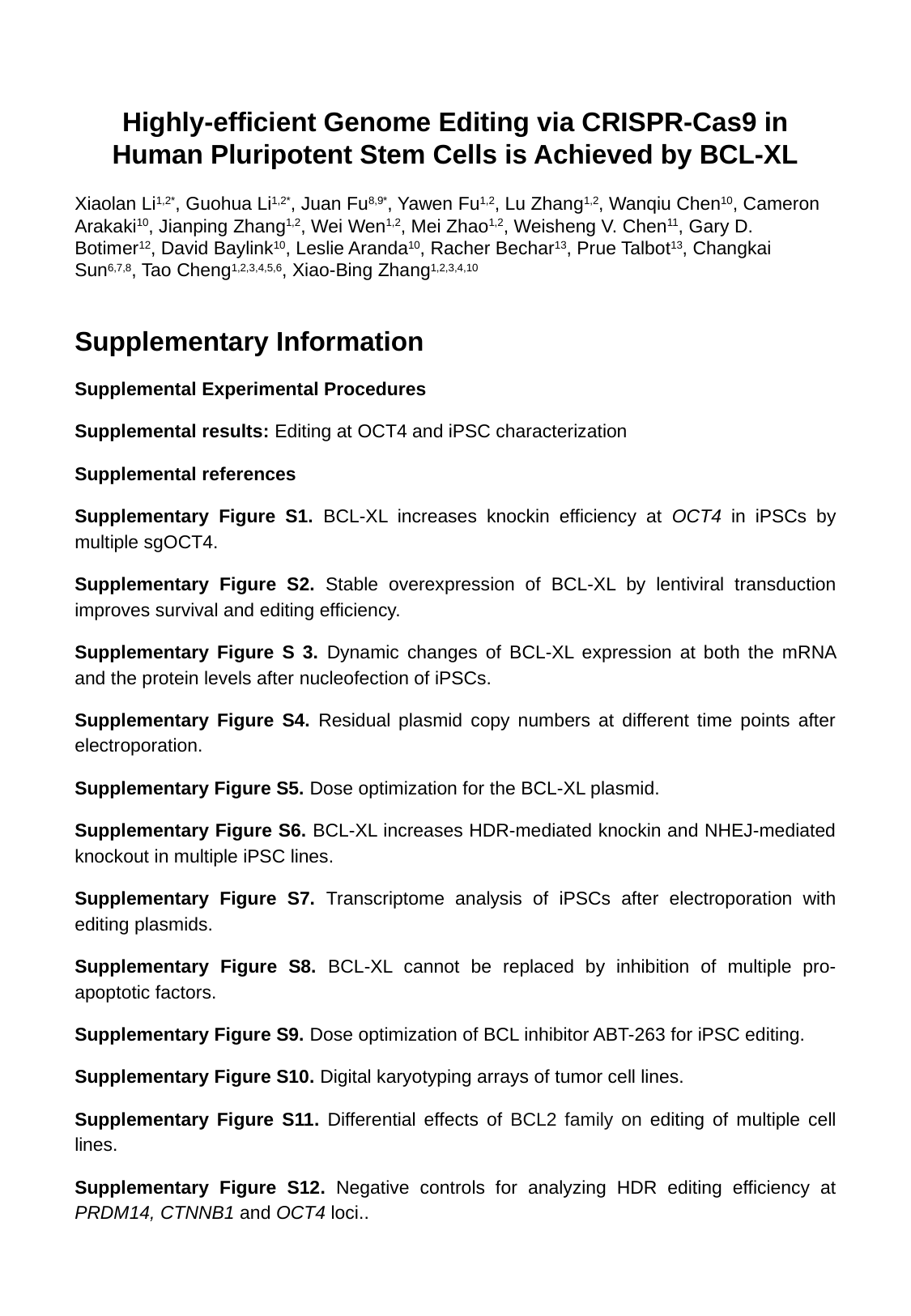

Highly-efficient Genome Editing via CRISPR-Cas9 in Human Pluripotent Stem Cells is Achieved by BCL-XL
Xiaolan Li1,2*, Guohua Li1,2*, Juan Fu8,9*, Yawen Fu1,2, Lu Zhang1,2, Wanqiu Chen10, Cameron Arakaki10, Jianping Zhang1,2, Wei Wen1,2, Mei Zhao1,2, Weisheng V. Chen11, Gary D. Botimer12, David Baylink10, Leslie Aranda10, Racher Bechar13, Prue Talbot13, Changkai Sun6,7,8, Tao Cheng1,2,3,4,5,6, Xiao-Bing Zhang1,2,3,4,10
Supplementary Information
Supplemental Experimental Procedures
Supplemental results: Editing at OCT4 and iPSC characterization
Supplemental references
Supplementary Figure S1. BCL-XL increases knockin efficiency at OCT4 in iPSCs by multiple sgOCT4.
Supplementary Figure S2. Stable overexpression of BCL-XL by lentiviral transduction improves survival and editing efficiency.
Supplementary Figure S 3. Dynamic changes of BCL-XL expression at both the mRNA and the protein levels after nucleofection of iPSCs.
Supplementary Figure S4. Residual plasmid copy numbers at different time points after electroporation.
Supplementary Figure S5. Dose optimization for the BCL-XL plasmid.
Supplementary Figure S6. BCL-XL increases HDR-mediated knockin and NHEJ-mediated knockout in multiple iPSC lines.
Supplementary Figure S7. Transcriptome analysis of iPSCs after electroporation with editing plasmids.
Supplementary Figure S8. BCL-XL cannot be replaced by inhibition of multiple pro-apoptotic factors.
Supplementary Figure S9. Dose optimization of BCL inhibitor ABT-263 for iPSC editing.
Supplementary Figure S10. Digital karyotyping arrays of tumor cell lines.
Supplementary Figure S11. Differential effects of BCL2 family on editing of multiple cell lines.
Supplementary Figure S12. Negative controls for analyzing HDR editing efficiency at PRDM14, CTNNB1 and OCT4 loci..

## Slide 2
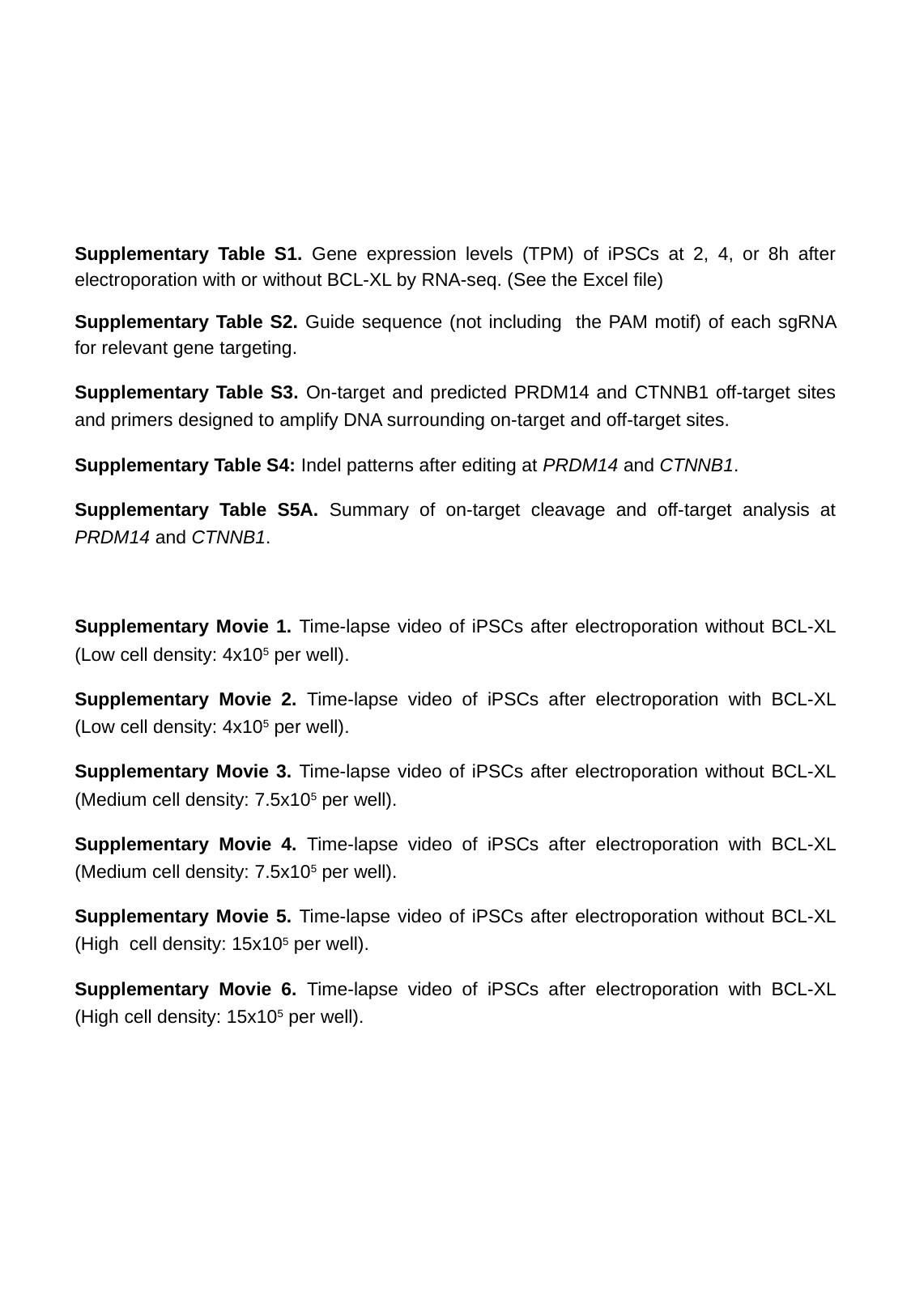

Supplementary Table S1. Gene expression levels (TPM) of iPSCs at 2, 4, or 8h after electroporation with or without BCL-XL by RNA-seq. (See the Excel file)
Supplementary Table S2. Guide sequence (not including the PAM motif) of each sgRNA for relevant gene targeting.
Supplementary Table S3. On-target and predicted PRDM14 and CTNNB1 off-target sites and primers designed to amplify DNA surrounding on-target and off-target sites.
Supplementary Table S4: Indel patterns after editing at PRDM14 and CTNNB1.
Supplementary Table S5A. Summary of on-target cleavage and off-target analysis at PRDM14 and CTNNB1.
Supplementary Movie 1. Time-lapse video of iPSCs after electroporation without BCL-XL (Low cell density: 4x10^5^ per well).
Supplementary Movie 2. Time-lapse video of iPSCs after electroporation with BCL-XL (Low cell density: 4x10^5^ per well).
Supplementary Movie 3. Time-lapse video of iPSCs after electroporation without BCL-XL (Medium cell density: 7.5x10^5^ per well).
Supplementary Movie 4. Time-lapse video of iPSCs after electroporation with BCL-XL (Medium cell density: 7.5x10^5^ per well).
Supplementary Movie 5. Time-lapse video of iPSCs after electroporation without BCL-XL (High cell density: 15x105 per well).
Supplementary Movie 6. Time-lapse video of iPSCs after electroporation with BCL-XL (High cell density: 15x105 per well).

## Slide 3
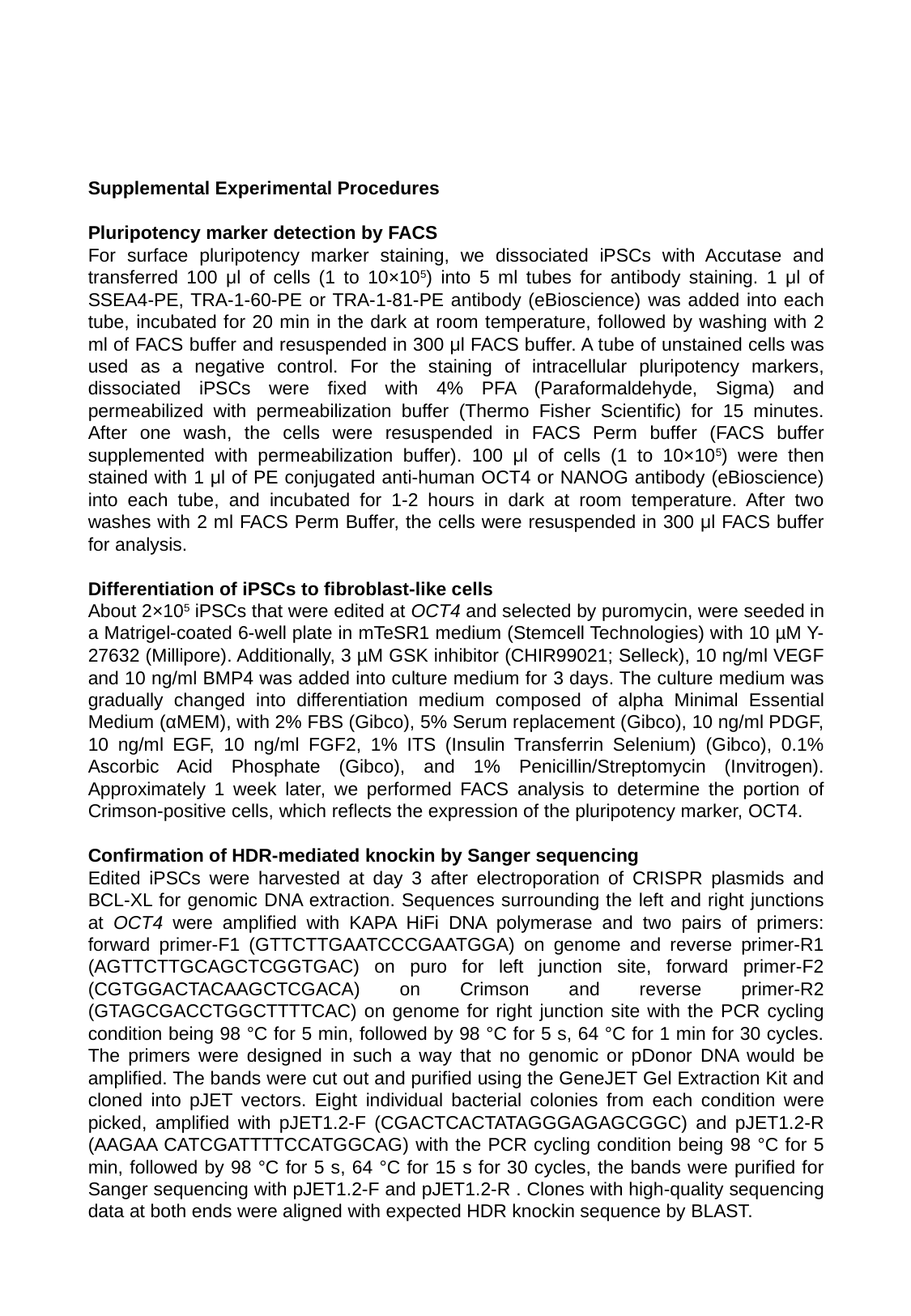

Supplemental Experimental Procedures
Pluripotency marker detection by FACS
For surface pluripotency marker staining, we dissociated iPSCs with Accutase and transferred 100 μl of cells (1 to 10×105) into 5 ml tubes for antibody staining. 1 μl of SSEA4-PE, TRA-1-60-PE or TRA-1-81-PE antibody (eBioscience) was added into each tube, incubated for 20 min in the dark at room temperature, followed by washing with 2 ml of FACS buffer and resuspended in 300 μl FACS buffer. A tube of unstained cells was used as a negative control. For the staining of intracellular pluripotency markers, dissociated iPSCs were fixed with 4% PFA (Paraformaldehyde, Sigma) and permeabilized with permeabilization buffer (Thermo Fisher Scientific) for 15 minutes. After one wash, the cells were resuspended in FACS Perm buffer (FACS buffer supplemented with permeabilization buffer). 100 μl of cells (1 to 10×105) were then stained with 1 μl of PE conjugated anti-human OCT4 or NANOG antibody (eBioscience) into each tube, and incubated for 1-2 hours in dark at room temperature. After two washes with 2 ml FACS Perm Buffer, the cells were resuspended in 300 μl FACS buffer for analysis.
Differentiation of iPSCs to fibroblast-like cells
About 2×10^5^ iPSCs that were edited at OCT4 and selected by puromycin, were seeded in a Matrigel-coated 6-well plate in mTeSR1 medium (Stemcell Technologies) with 10 µM Y-27632 (Millipore). Additionally, 3 µM GSK inhibitor (CHIR99021; Selleck), 10 ng/ml VEGF and 10 ng/ml BMP4 was added into culture medium for 3 days. The culture medium was gradually changed into differentiation medium composed of alpha Minimal Essential Medium (αMEM), with 2% FBS (Gibco), 5% Serum replacement (Gibco), 10 ng/ml PDGF, 10 ng/ml EGF, 10 ng/ml FGF2, 1% ITS (Insulin Transferrin Selenium) (Gibco), 0.1% Ascorbic Acid Phosphate (Gibco), and 1% Penicillin/Streptomycin (Invitrogen). Approximately 1 week later, we performed FACS analysis to determine the portion of Crimson-positive cells, which reflects the expression of the pluripotency marker, OCT4.
Confirmation of HDR-mediated knockin by Sanger sequencing
Edited iPSCs were harvested at day 3 after electroporation of CRISPR plasmids and BCL-XL for genomic DNA extraction. Sequences surrounding the left and right junctions at OCT4 were amplified with KAPA HiFi DNA polymerase and two pairs of primers: forward primer-F1 (GTTCTTGAATCCCGAATGGA) on genome and reverse primer-R1 (AGTTCTTGCAGCTCGGTGAC) on puro for left junction site, forward primer-F2 (CGTGGACTACAAGCTCGACA) on Crimson and reverse primer-R2 (GTAGCGACCTGGCTTTTCAC) on genome for right junction site with the PCR cycling condition being 98 °C for 5 min, followed by 98 °C for 5 s, 64 °C for 1 min for 30 cycles. The primers were designed in such a way that no genomic or pDonor DNA would be amplified. The bands were cut out and purified using the GeneJET Gel Extraction Kit and cloned into pJET vectors. Eight individual bacterial colonies from each condition were picked, amplified with pJET1.2-F (CGACTCACTATAGGGAGAGCGGC) and pJET1.2-R (AAGAA CATCGATTTTCCATGGCAG) with the PCR cycling condition being 98 °C for 5 min, followed by 98 °C for 5 s, 64 °C for 15 s for 30 cycles, the bands were purified for Sanger sequencing with pJET1.2-F and pJET1.2-R . Clones with high-quality sequencing data at both ends were aligned with expected HDR knockin sequence by BLAST.

## Slide 4
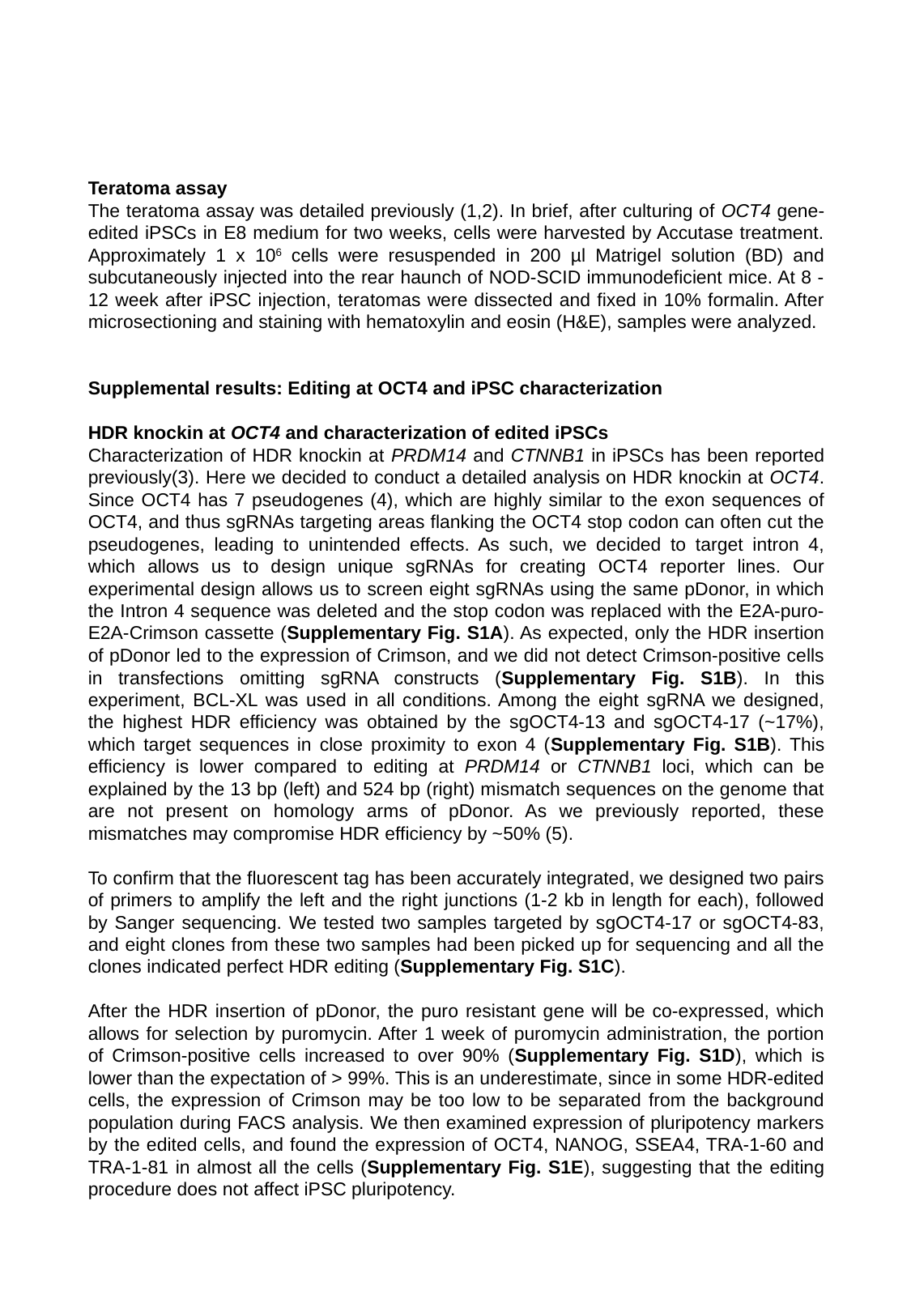

Teratoma assay
The teratoma assay was detailed previously (1,2). In brief, after culturing of OCT4 gene-edited iPSCs in E8 medium for two weeks, cells were harvested by Accutase treatment. Approximately 1 x 10^6^ cells were resuspended in 200 µl Matrigel solution (BD) and subcutaneously injected into the rear haunch of NOD-SCID immunodeficient mice. At 8 - 12 week after iPSC injection, teratomas were dissected and fixed in 10% formalin. After microsectioning and staining with hematoxylin and eosin (H&E), samples were analyzed.
Supplemental results: Editing at OCT4 and iPSC characterization
HDR knockin at OCT4 and characterization of edited iPSCs
Characterization of HDR knockin at PRDM14 and CTNNB1 in iPSCs has been reported previously(3). Here we decided to conduct a detailed analysis on HDR knockin at OCT4. Since OCT4 has 7 pseudogenes (4), which are highly similar to the exon sequences of OCT4, and thus sgRNAs targeting areas flanking the OCT4 stop codon can often cut the pseudogenes, leading to unintended effects. As such, we decided to target intron 4, which allows us to design unique sgRNAs for creating OCT4 reporter lines. Our experimental design allows us to screen eight sgRNAs using the same pDonor, in which the Intron 4 sequence was deleted and the stop codon was replaced with the E2A-puro-E2A-Crimson cassette (Supplementary Fig. S1A). As expected, only the HDR insertion of pDonor led to the expression of Crimson, and we did not detect Crimson-positive cells in transfections omitting sgRNA constructs (Supplementary Fig. S1B). In this experiment, BCL-XL was used in all conditions. Among the eight sgRNA we designed, the highest HDR efficiency was obtained by the sgOCT4-13 and sgOCT4-17 (~17%), which target sequences in close proximity to exon 4 (Supplementary Fig. S1B). This efficiency is lower compared to editing at PRDM14 or CTNNB1 loci, which can be explained by the 13 bp (left) and 524 bp (right) mismatch sequences on the genome that are not present on homology arms of pDonor. As we previously reported, these mismatches may compromise HDR efficiency by ~50% (5).
To confirm that the fluorescent tag has been accurately integrated, we designed two pairs of primers to amplify the left and the right junctions (1-2 kb in length for each), followed by Sanger sequencing. We tested two samples targeted by sgOCT4-17 or sgOCT4-83, and eight clones from these two samples had been picked up for sequencing and all the clones indicated perfect HDR editing (Supplementary Fig. S1C).
After the HDR insertion of pDonor, the puro resistant gene will be co-expressed, which allows for selection by puromycin. After 1 week of puromycin administration, the portion of Crimson-positive cells increased to over 90% (Supplementary Fig. S1D), which is lower than the expectation of > 99%. This is an underestimate, since in some HDR-edited cells, the expression of Crimson may be too low to be separated from the background population during FACS analysis. We then examined expression of pluripotency markers by the edited cells, and found the expression of OCT4, NANOG, SSEA4, TRA-1-60 and TRA-1-81 in almost all the cells (Supplementary Fig. S1E), suggesting that the editing procedure does not affect iPSC pluripotency.

## Slide 5
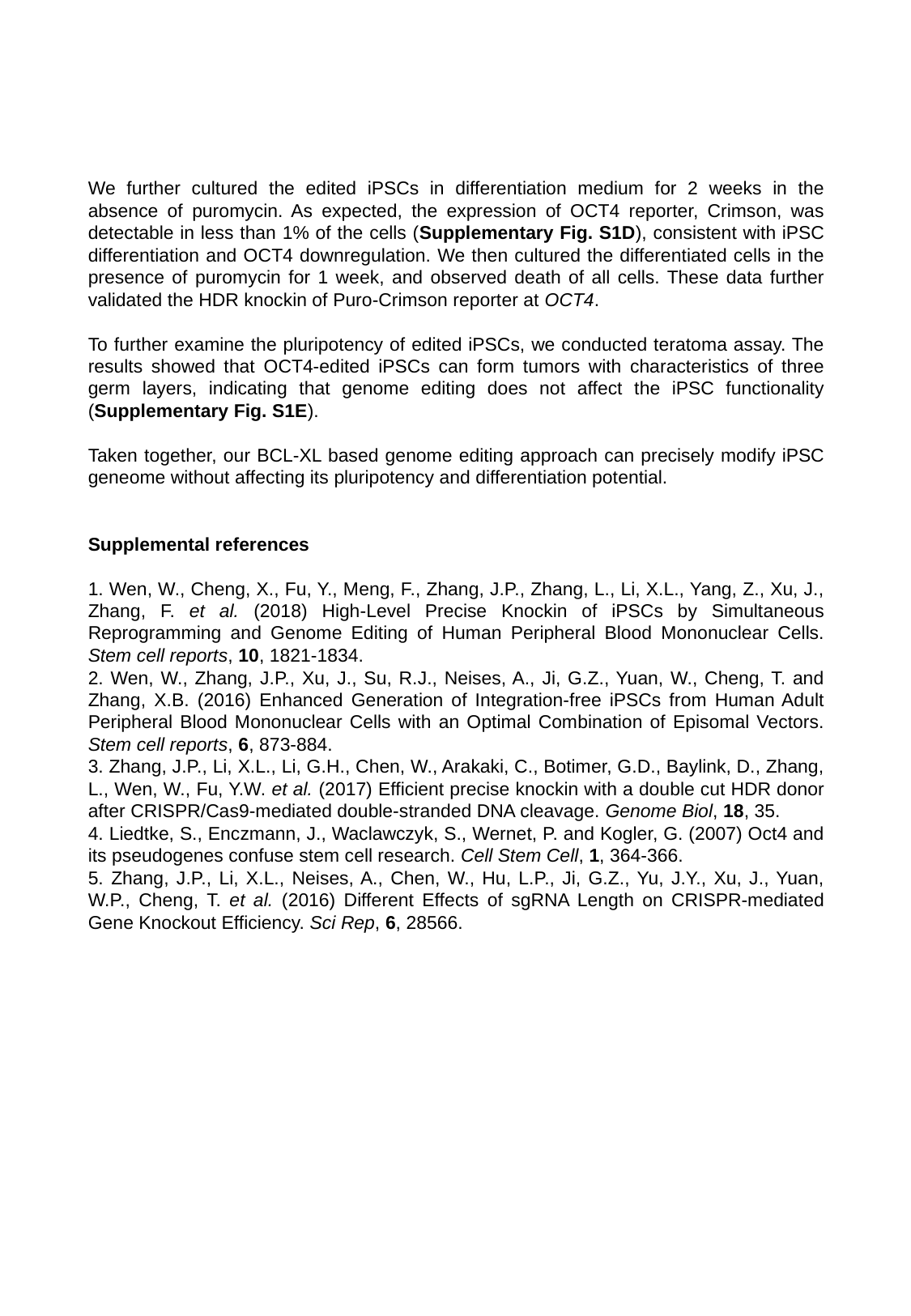

We further cultured the edited iPSCs in differentiation medium for 2 weeks in the absence of puromycin. As expected, the expression of OCT4 reporter, Crimson, was detectable in less than 1% of the cells (Supplementary Fig. S1D), consistent with iPSC differentiation and OCT4 downregulation. We then cultured the differentiated cells in the presence of puromycin for 1 week, and observed death of all cells. These data further validated the HDR knockin of Puro-Crimson reporter at OCT4.
To further examine the pluripotency of edited iPSCs, we conducted teratoma assay. The results showed that OCT4-edited iPSCs can form tumors with characteristics of three germ layers, indicating that genome editing does not affect the iPSC functionality (Supplementary Fig. S1E).
Taken together, our BCL-XL based genome editing approach can precisely modify iPSC geneome without affecting its pluripotency and differentiation potential.
Supplemental references
1. Wen, W., Cheng, X., Fu, Y., Meng, F., Zhang, J.P., Zhang, L., Li, X.L., Yang, Z., Xu, J., Zhang, F. et al. (2018) High-Level Precise Knockin of iPSCs by Simultaneous Reprogramming and Genome Editing of Human Peripheral Blood Mononuclear Cells. Stem cell reports, 10, 1821-1834.
2. Wen, W., Zhang, J.P., Xu, J., Su, R.J., Neises, A., Ji, G.Z., Yuan, W., Cheng, T. and Zhang, X.B. (2016) Enhanced Generation of Integration-free iPSCs from Human Adult Peripheral Blood Mononuclear Cells with an Optimal Combination of Episomal Vectors. Stem cell reports, 6, 873-884.
3. Zhang, J.P., Li, X.L., Li, G.H., Chen, W., Arakaki, C., Botimer, G.D., Baylink, D., Zhang, L., Wen, W., Fu, Y.W. et al. (2017) Efficient precise knockin with a double cut HDR donor after CRISPR/Cas9-mediated double-stranded DNA cleavage. Genome Biol, 18, 35.
4. Liedtke, S., Enczmann, J., Waclawczyk, S., Wernet, P. and Kogler, G. (2007) Oct4 and its pseudogenes confuse stem cell research. Cell Stem Cell, 1, 364-366.
5. Zhang, J.P., Li, X.L., Neises, A., Chen, W., Hu, L.P., Ji, G.Z., Yu, J.Y., Xu, J., Yuan, W.P., Cheng, T. et al. (2016) Different Effects of sgRNA Length on CRISPR-mediated Gene Knockout Efficiency. Sci Rep, 6, 28566.

## Slide 6
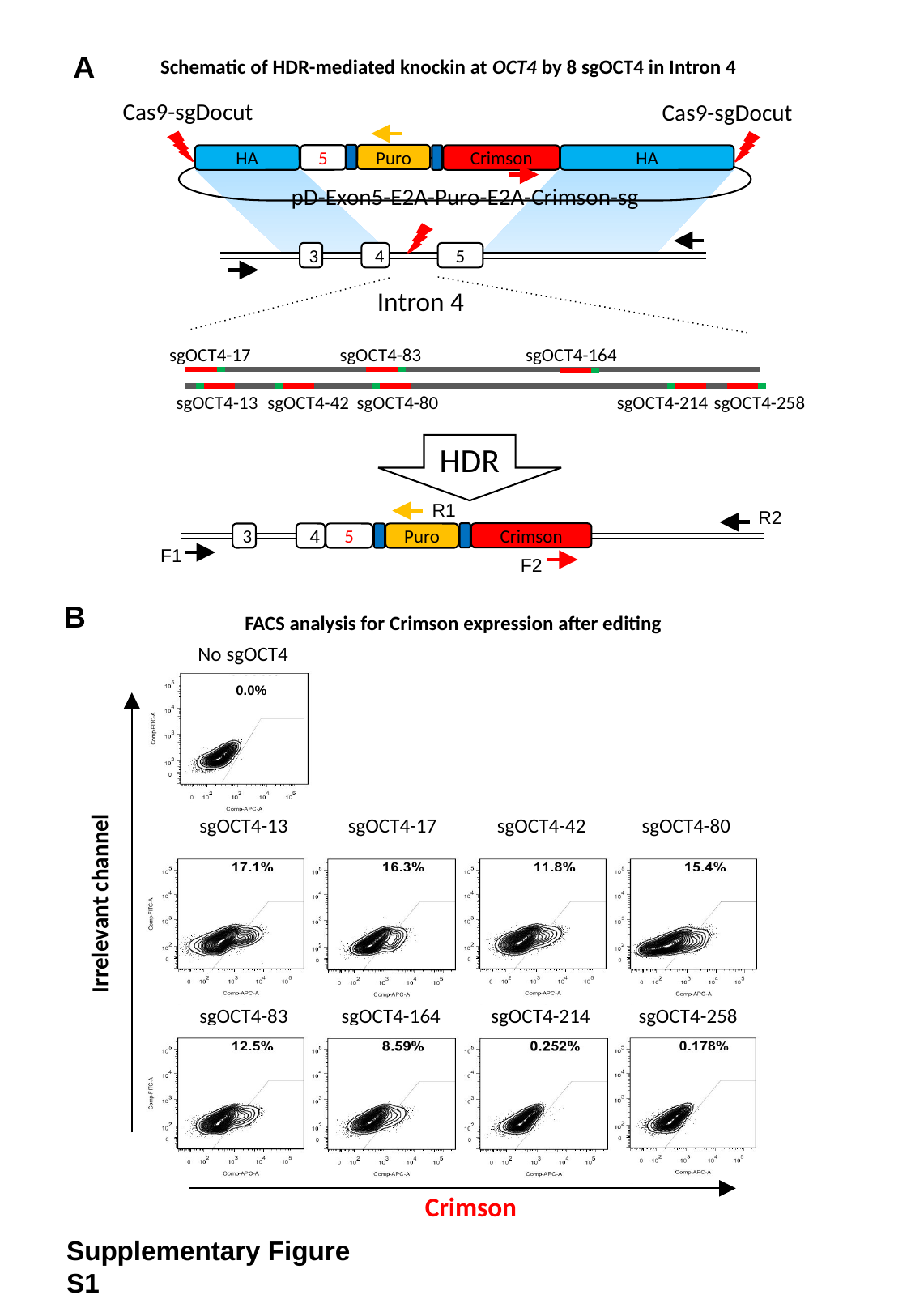

A
Schematic of HDR-mediated knockin at OCT4 by 8 sgOCT4 in Intron 4
Cas9-sgDocut
Cas9-sgDocut
HA
Crimson
HA
pD-Exon5-E2A-Puro-E2A-Crimson-sg
5
3
4
5
Puro
Intron 4
sgOCT4-17
sgOCT4-83
sgOCT4-164
sgOCT4-13
sgOCT4-42
sgOCT4-80
sgOCT4-214
sgOCT4-258
HDR
R1
R2
Crimson
3
4
5
Puro
F1
F2
B
FACS analysis for Crimson expression after editing
No sgOCT4
0.0%
sgOCT4-13
sgOCT4-17
sgOCT4-42
sgOCT4-80
Irrelevant channel
sgOCT4-214
sgOCT4-258
sgOCT4-83
sgOCT4-164
Crimson
Supplementary Figure S1

## Slide 7
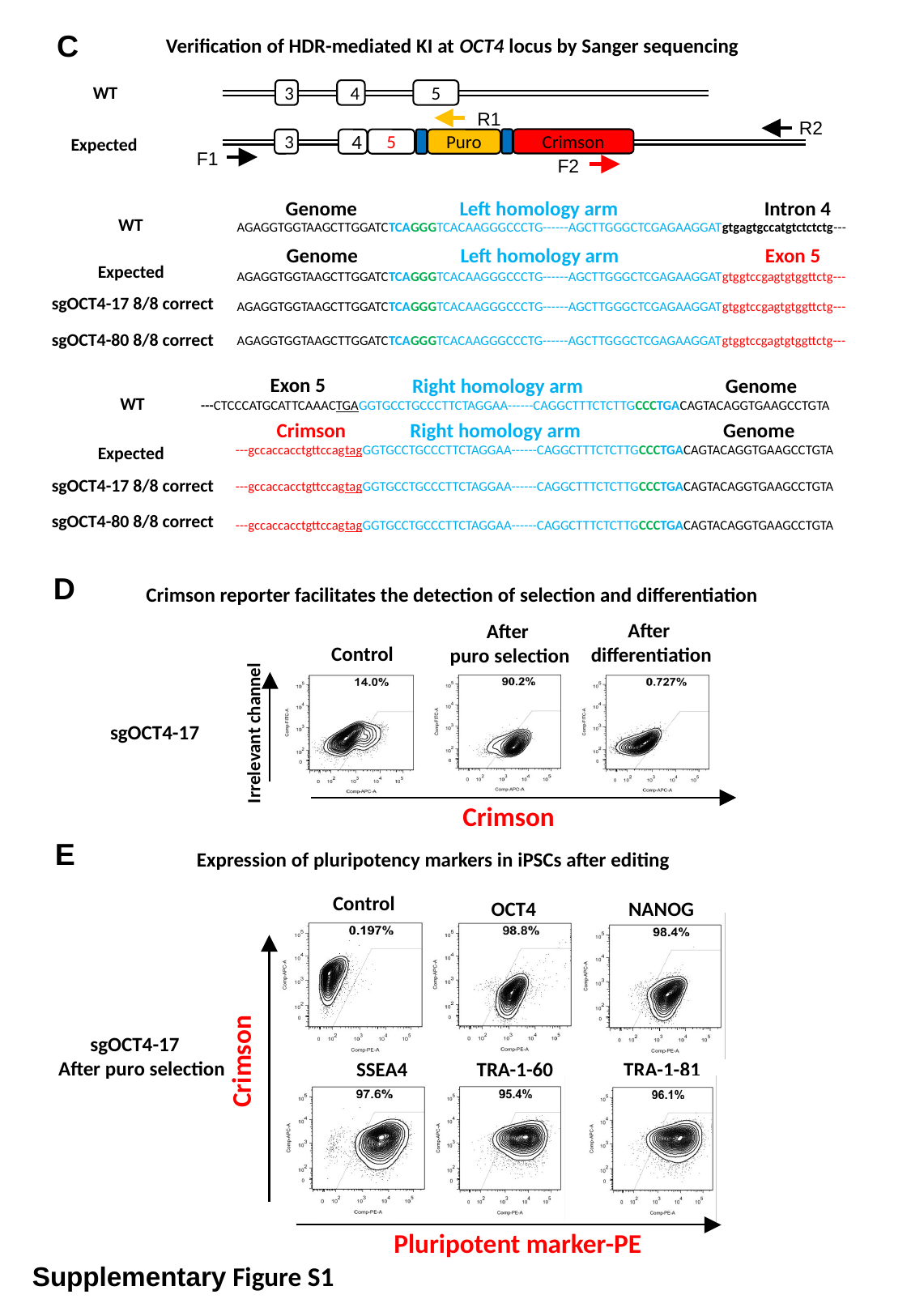

C
Verification of HDR-mediated KI at OCT4 locus by Sanger sequencing
WT
3
4
5
R1
R2
Expected
Crimson
3
4
5
Puro
F1
F2
Genome
Left homology arm
Intron 4
WT
AGAGGTGGTAAGCTTGGATCTCAGGGTCACAAGGGCCCTG------AGCTTGGGCTCGAGAAGGATgtgagtgccatgtctctctg---
Genome
Left homology arm
Exon 5
Expected
AGAGGTGGTAAGCTTGGATCTCAGGGTCACAAGGGCCCTG------AGCTTGGGCTCGAGAAGGATgtggtccgagtgtggttctg---
sgOCT4-17 8/8 correct
AGAGGTGGTAAGCTTGGATCTCAGGGTCACAAGGGCCCTG------AGCTTGGGCTCGAGAAGGATgtggtccgagtgtggttctg---
sgOCT4-80 8/8 correct
AGAGGTGGTAAGCTTGGATCTCAGGGTCACAAGGGCCCTG------AGCTTGGGCTCGAGAAGGATgtggtccgagtgtggttctg---
Exon 5
Right homology arm
Genome
WT
---CTCCCATGCATTCAAACTGAGGTGCCTGCCCTTCTAGGAA------CAGGCTTTCTCTTGCCCTGACAGTACAGGTGAAGCCTGTA
Crimson
Right homology arm
Genome
---gccaccacctgttccagtagGGTGCCTGCCCTTCTAGGAA------CAGGCTTTCTCTTGCCCTGACAGTACAGGTGAAGCCTGTA
Expected
sgOCT4-17 8/8 correct
---gccaccacctgttccagtagGGTGCCTGCCCTTCTAGGAA------CAGGCTTTCTCTTGCCCTGACAGTACAGGTGAAGCCTGTA
sgOCT4-80 8/8 correct
---gccaccacctgttccagtagGGTGCCTGCCCTTCTAGGAA------CAGGCTTTCTCTTGCCCTGACAGTACAGGTGAAGCCTGTA
D
Crimson reporter facilitates the detection of selection and differentiation
After
differentiation
After
puro selection
Control
sgOCT4-17
Irrelevant channel
Crimson
E
Expression of pluripotency markers in iPSCs after editing
Control
OCT4
NANOG
sgOCT4-17
After puro selection
Crimson
SSEA4
TRA-1-60
TRA-1-81
Pluripotent marker-PE
Supplementary Figure S1

## Slide 8
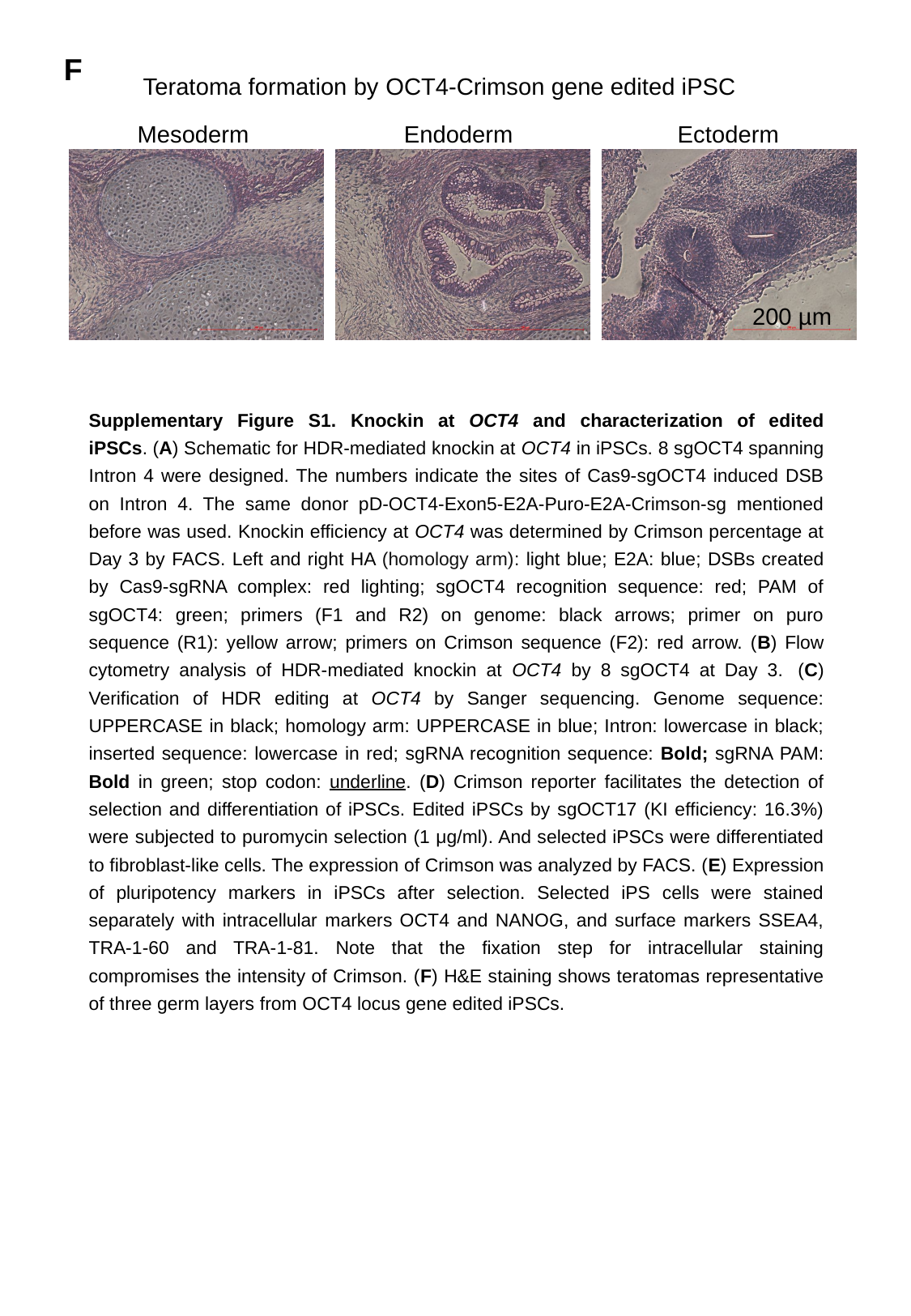

F
Teratoma formation by OCT4-Crimson gene edited iPSC
Mesoderm
Endoderm
Ectoderm
200 µm
Supplementary Figure S1. Knockin at OCT4 and characterization of edited iPSCs. (A) Schematic for HDR-mediated knockin at OCT4 in iPSCs. 8 sgOCT4 spanning Intron 4 were designed. The numbers indicate the sites of Cas9-sgOCT4 induced DSB on Intron 4. The same donor pD-OCT4-Exon5-E2A-Puro-E2A-Crimson-sg mentioned before was used. Knockin efficiency at OCT4 was determined by Crimson percentage at Day 3 by FACS. Left and right HA (homology arm): light blue; E2A: blue; DSBs created by Cas9-sgRNA complex: red lighting; sgOCT4 recognition sequence: red; PAM of sgOCT4: green; primers (F1 and R2) on genome: black arrows; primer on puro sequence (R1): yellow arrow; primers on Crimson sequence (F2): red arrow. (B) Flow cytometry analysis of HDR-mediated knockin at OCT4 by 8 sgOCT4 at Day 3.  (C) Verification of HDR editing at OCT4 by Sanger sequencing. Genome sequence: UPPERCASE in black; homology arm: UPPERCASE in blue; Intron: lowercase in black; inserted sequence: lowercase in red; sgRNA recognition sequence: Bold; sgRNA PAM: Bold in green; stop codon: underline. (D) Crimson reporter facilitates the detection of selection and differentiation of iPSCs. Edited iPSCs by sgOCT17 (KI efficiency: 16.3%) were subjected to puromycin selection (1 μg/ml). And selected iPSCs were differentiated to fibroblast-like cells. The expression of Crimson was analyzed by FACS. (E) Expression of pluripotency markers in iPSCs after selection. Selected iPS cells were stained separately with intracellular markers OCT4 and NANOG, and surface markers SSEA4, TRA-1-60 and TRA-1-81. Note that the fixation step for intracellular staining compromises the intensity of Crimson. (F) H&E staining shows teratomas representative of three germ layers from OCT4 locus gene edited iPSCs.

## Slide 9
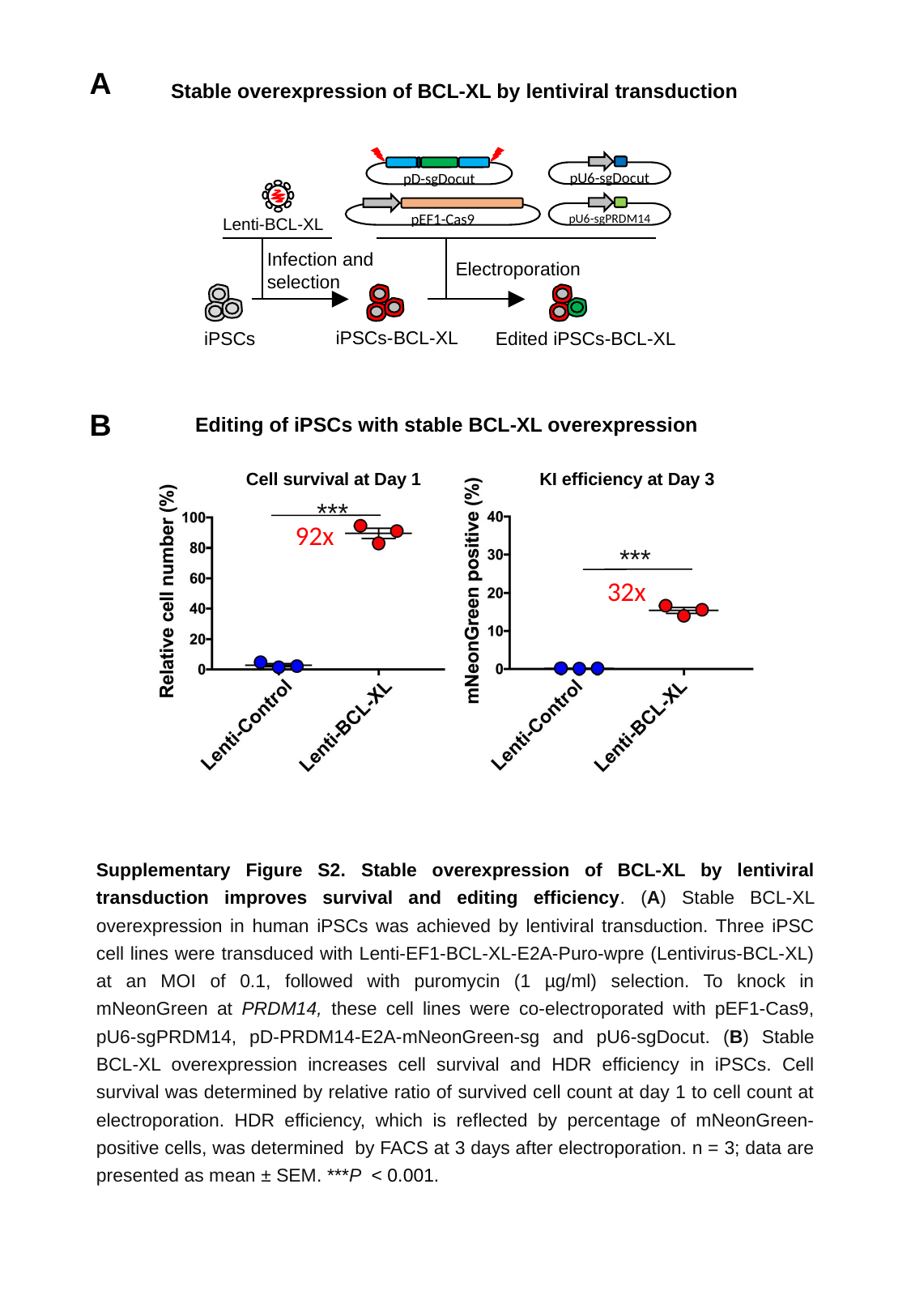

A
Stable overexpression of BCL-XL by lentiviral transduction
pD-sgDocut
pU6-sgDocut
pU6-sgPRDM14
pEF1-Cas9
Lenti-BCL-XL
Infection and selection
Electroporation
iPSCs-BCL-XL
Edited iPSCs-BCL-XL
iPSCs
B
Editing of iPSCs with stable BCL-XL overexpression
Cell survival at Day 1
KI efficiency at Day 3
***
92x
***
32x
Supplementary Figure S2. Stable overexpression of BCL-XL by lentiviral transduction improves survival and editing efficiency. (A) Stable BCL-XL overexpression in human iPSCs was achieved by lentiviral transduction. Three iPSC cell lines were transduced with Lenti-EF1-BCL-XL-E2A-Puro-wpre (Lentivirus-BCL-XL) at an MOI of 0.1, followed with puromycin (1 µg/ml) selection. To knock in mNeonGreen at PRDM14, these cell lines were co-electroporated with pEF1-Cas9, pU6-sgPRDM14, pD-PRDM14-E2A-mNeonGreen-sg and pU6-sgDocut. (B) Stable BCL-XL overexpression increases cell survival and HDR efficiency in iPSCs. Cell survival was determined by relative ratio of survived cell count at day 1 to cell count at electroporation. HDR efficiency, which is reflected by percentage of mNeonGreen-positive cells, was determined by FACS at 3 days after electroporation. n = 3; data are presented as mean ± SEM. ***P < 0.001.

## Slide 10
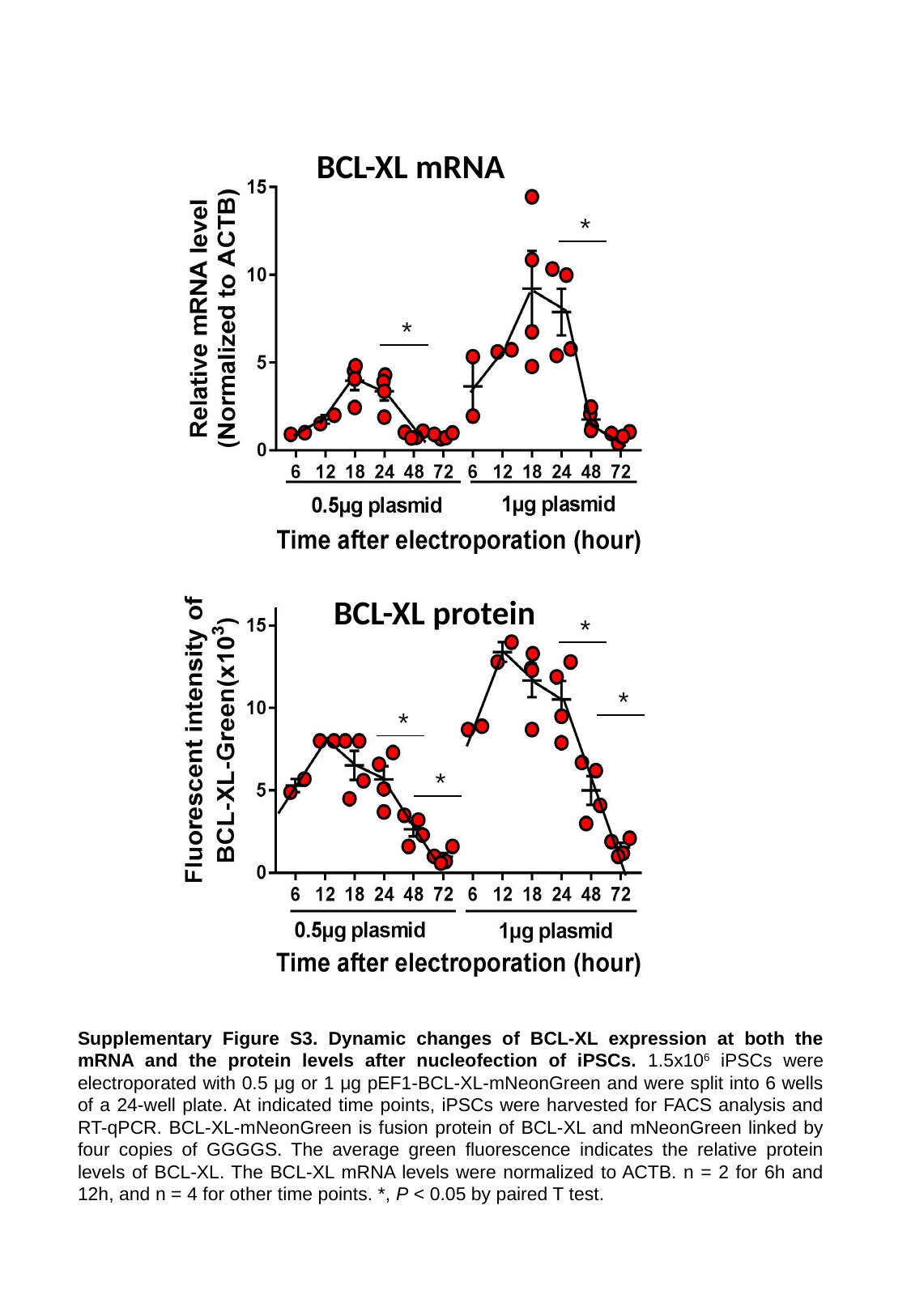

# BCL-XL mRNA
*
*
BCL-XL protein
*
*
*
*
Supplementary Figure S3. Dynamic changes of BCL-XL expression at both the mRNA and the protein levels after nucleofection of iPSCs. 1.5x10^6^ iPSCs were electroporated with 0.5 μg or 1 μg pEF1-BCL-XL-mNeonGreen and were split into 6 wells of a 24-well plate. At indicated time points, iPSCs were harvested for FACS analysis and RT-qPCR. BCL-XL-mNeonGreen is fusion protein of BCL-XL and mNeonGreen linked by four copies of GGGGS. The average green fluorescence indicates the relative protein levels of BCL-XL. The BCL-XL mRNA levels were normalized to ACTB. n = 2 for 6h and 12h, and n = 4 for other time points. *, P < 0.05 by paired T test.

## Slide 11
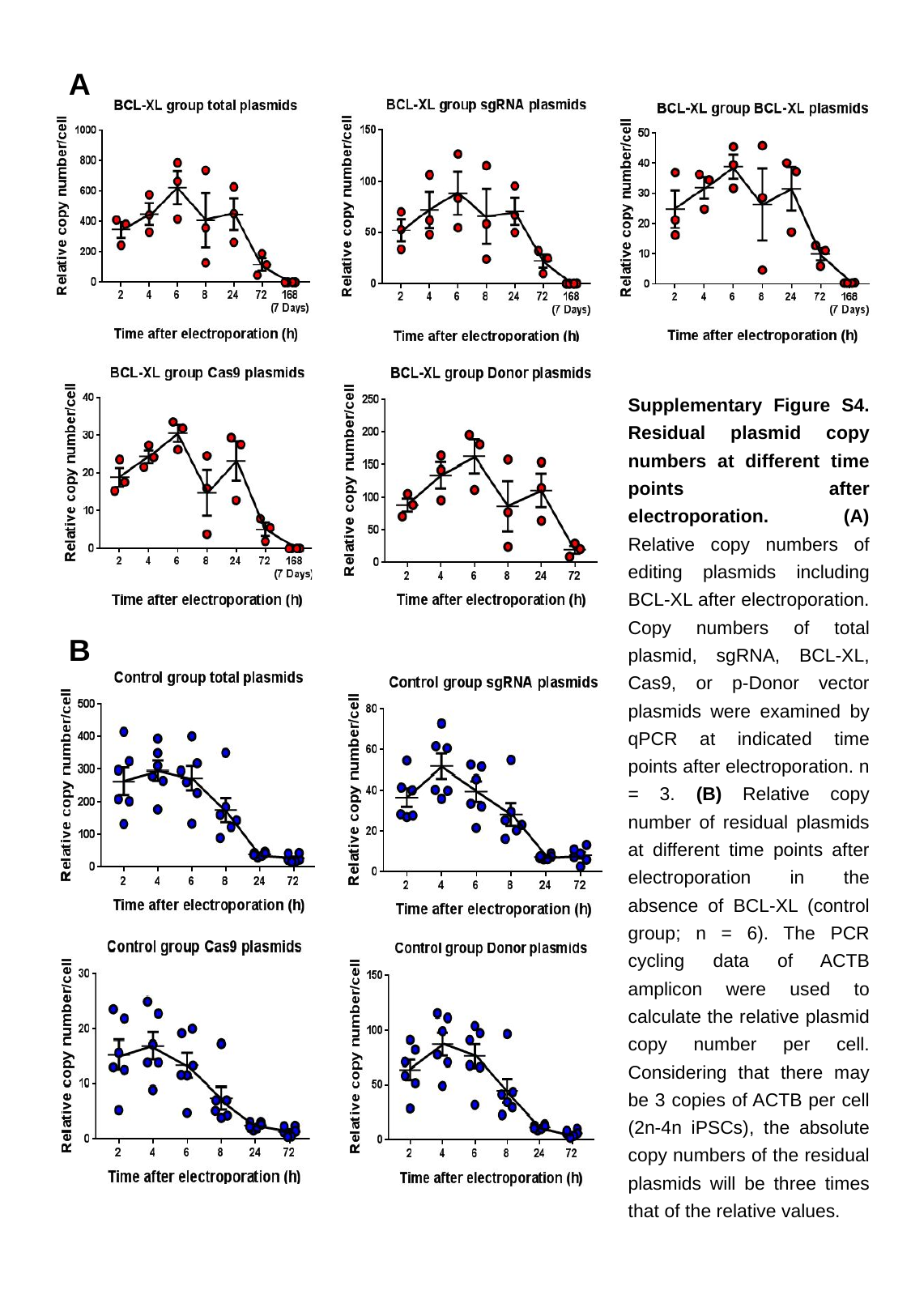

A
Supplementary Figure S4. Residual plasmid copy numbers at different time points after electroporation. (A) Relative copy numbers of editing plasmids including BCL-XL after electroporation. Copy numbers of total plasmid, sgRNA, BCL-XL, Cas9, or p-Donor vector plasmids were examined by qPCR at indicated time points after electroporation. n = 3. (B) Relative copy number of residual plasmids at different time points after electroporation in the absence of BCL-XL (control group; n = 6). The PCR cycling data of ACTB amplicon were used to calculate the relative plasmid copy number per cell. Considering that there may be 3 copies of ACTB per cell (2n-4n iPSCs), the absolute copy numbers of the residual plasmids will be three times that of the relative values.
B

## Slide 12
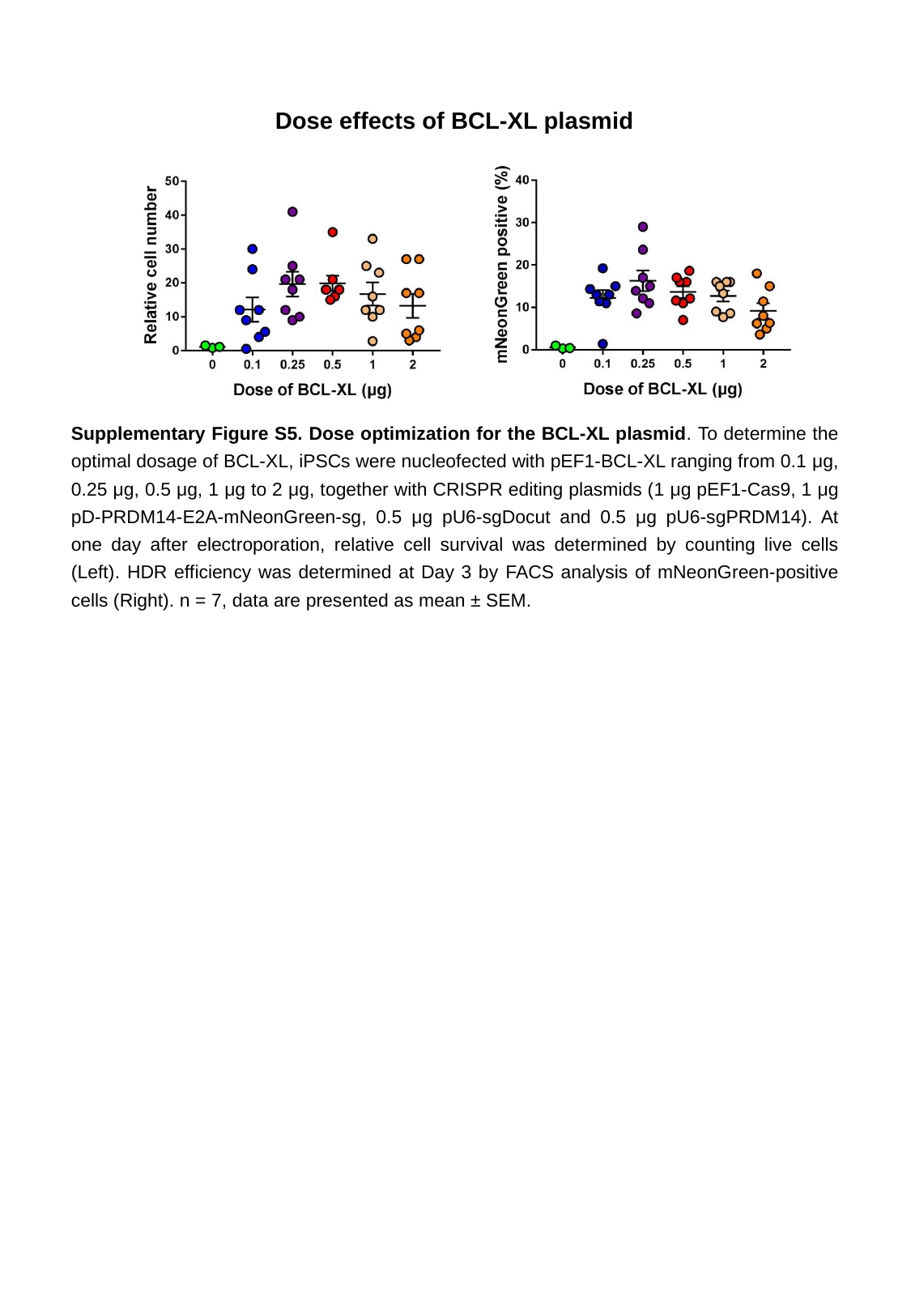

Dose effects of BCL-XL plasmid
Supplementary Figure S5. Dose optimization for the BCL-XL plasmid. To determine the optimal dosage of BCL-XL, iPSCs were nucleofected with pEF1-BCL-XL ranging from 0.1 μg, 0.25 μg, 0.5 μg, 1 μg to 2 μg, together with CRISPR editing plasmids (1 μg pEF1-Cas9, 1 μg pD-PRDM14-E2A-mNeonGreen-sg, 0.5 μg pU6-sgDocut and 0.5 μg pU6-sgPRDM14). At one day after electroporation, relative cell survival was determined by counting live cells (Left). HDR efficiency was determined at Day 3 by FACS analysis of mNeonGreen-positive cells (Right). n = 7, data are presented as mean ± SEM.

## Slide 13
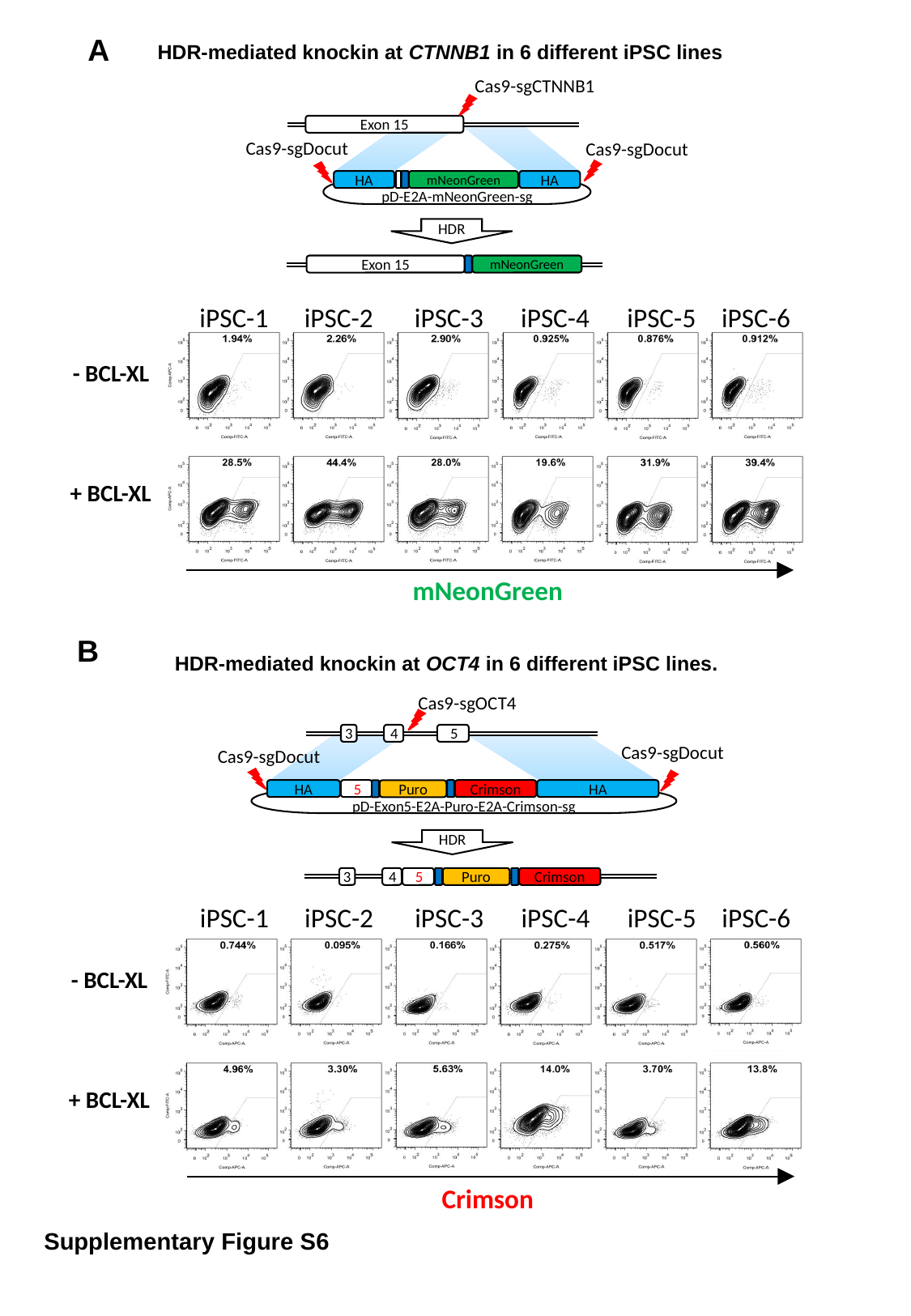

A
HDR-mediated knockin at CTNNB1 in 6 different iPSC lines
Cas9-sgCTNNB1
Exon 15
Cas9-sgDocut
Cas9-sgDocut
HA
mNeonGreen
HA
pD-E2A-mNeonGreen-sg
HDR
Exon 15
mNeonGreen
iPSC-1
iPSC-2
iPSC-3
iPSC-4
iPSC-5
iPSC-6
- BCL-XL
+ BCL-XL
mNeonGreen
B
HDR-mediated knockin at OCT4 in 6 different iPSC lines.
Cas9-sgOCT4
3
4
5
Cas9-sgDocut
Cas9-sgDocut
HA
Crimson
HA
pD-Exon5-E2A-Puro-E2A-Crimson-sg
5
Puro
HDR
3
4
5
Puro
Crimson
iPSC-1
iPSC-2
iPSC-3
iPSC-4
iPSC-5
iPSC-6
- BCL-XL
+ BCL-XL
Crimson
Supplementary Figure S6

## Slide 14
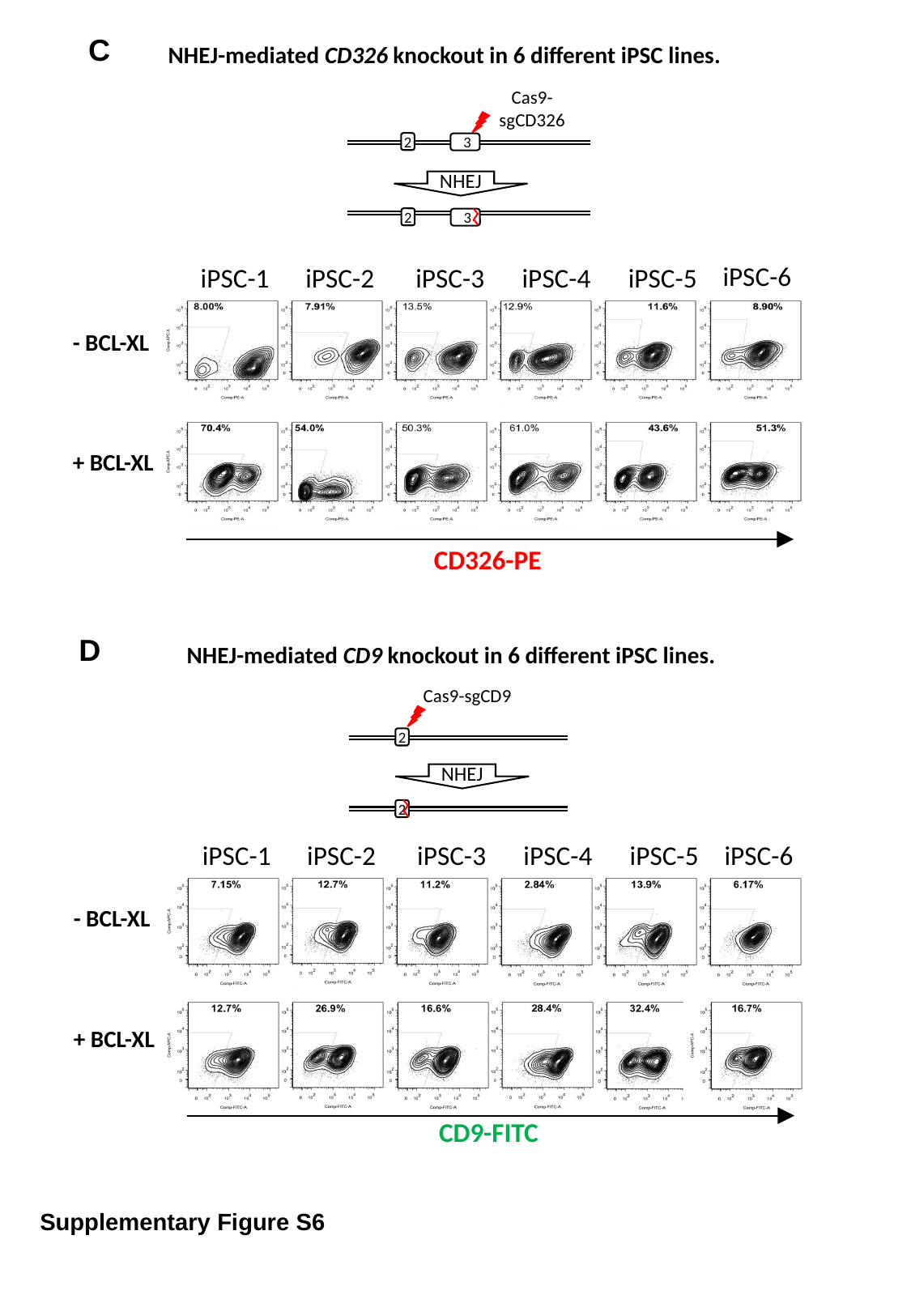

C
NHEJ-mediated CD326 knockout in 6 different iPSC lines.
Cas9-sgCD326
2
3
NHEJ
2
3
iPSC-6
iPSC-1
iPSC-2
iPSC-3
iPSC-4
iPSC-5
- BCL-XL
+ BCL-XL
CD326-PE
D
NHEJ-mediated CD9 knockout in 6 different iPSC lines.
Cas9-sgCD9
2
NHEJ
2
iPSC-1
iPSC-2
iPSC-3
iPSC-4
iPSC-5
iPSC-6
- BCL-XL
+ BCL-XL
CD9-FITC
Supplementary Figure S6

## Slide 15
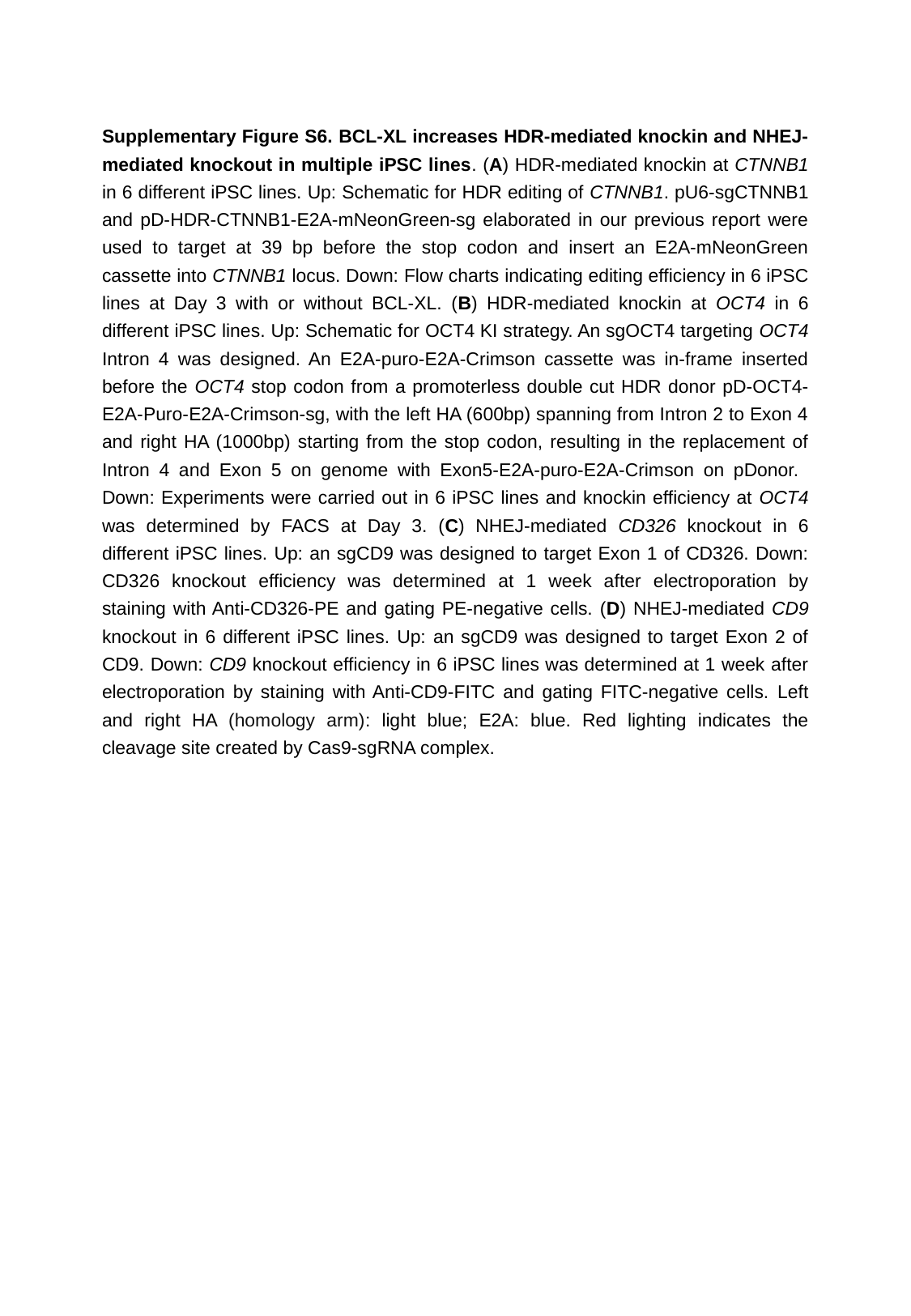

Supplementary Figure S6. BCL-XL increases HDR-mediated knockin and NHEJ-mediated knockout in multiple iPSC lines. (A) HDR-mediated knockin at CTNNB1 in 6 different iPSC lines. Up: Schematic for HDR editing of CTNNB1. pU6-sgCTNNB1 and pD-HDR-CTNNB1-E2A-mNeonGreen-sg elaborated in our previous report were used to target at 39 bp before the stop codon and insert an E2A-mNeonGreen cassette into CTNNB1 locus. Down: Flow charts indicating editing efficiency in 6 iPSC lines at Day 3 with or without BCL-XL. (B) HDR-mediated knockin at OCT4 in 6 different iPSC lines. Up: Schematic for OCT4 KI strategy. An sgOCT4 targeting OCT4 Intron 4 was designed. An E2A-puro-E2A-Crimson cassette was in-frame inserted before the OCT4 stop codon from a promoterless double cut HDR donor pD-OCT4-E2A-Puro-E2A-Crimson-sg, with the left HA (600bp) spanning from Intron 2 to Exon 4 and right HA (1000bp) starting from the stop codon, resulting in the replacement of Intron 4 and Exon 5 on genome with Exon5-E2A-puro-E2A-Crimson on pDonor. Down: Experiments were carried out in 6 iPSC lines and knockin efficiency at OCT4 was determined by FACS at Day 3. (C) NHEJ-mediated CD326 knockout in 6 different iPSC lines. Up: an sgCD9 was designed to target Exon 1 of CD326. Down: CD326 knockout efficiency was determined at 1 week after electroporation by staining with Anti-CD326-PE and gating PE-negative cells. (D) NHEJ-mediated CD9 knockout in 6 different iPSC lines. Up: an sgCD9 was designed to target Exon 2 of CD9. Down: CD9 knockout efficiency in 6 iPSC lines was determined at 1 week after electroporation by staining with Anti-CD9-FITC and gating FITC-negative cells. Left and right HA (homology arm): light blue; E2A: blue. Red lighting indicates the cleavage site created by Cas9-sgRNA complex.

## Slide 16
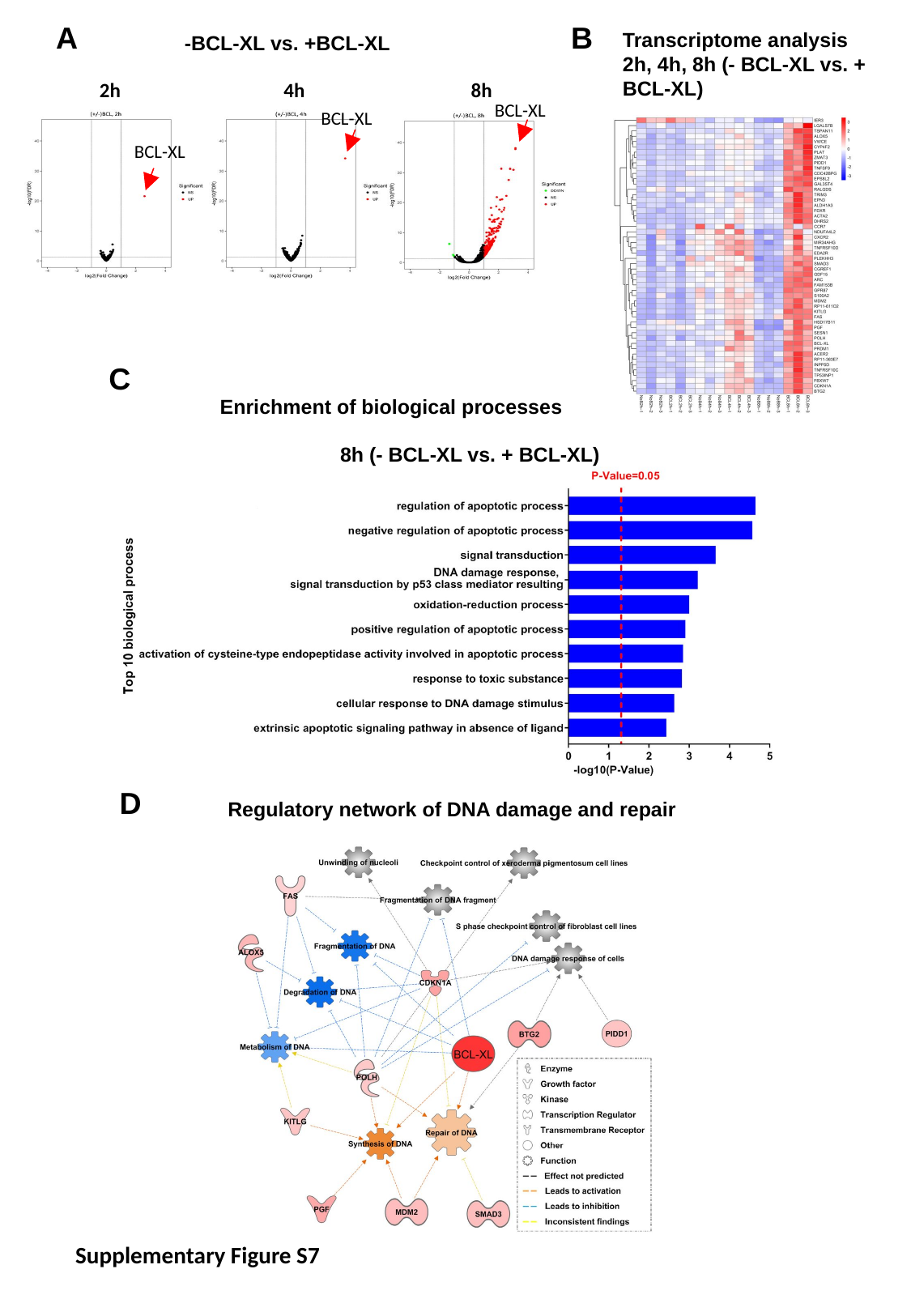

B
A
Transcriptome analysis
2h, 4h, 8h (- BCL-XL vs. + BCL-XL)
-BCL-XL vs. +BCL-XL
2h
4h
8h
BCL-XL
BCL-XL
BCL-XL
C
Enrichment of biological processes
8h (- BCL-XL vs. + BCL-XL)
D
Regulatory network of DNA damage and repair
Supplementary Figure S7

## Slide 17
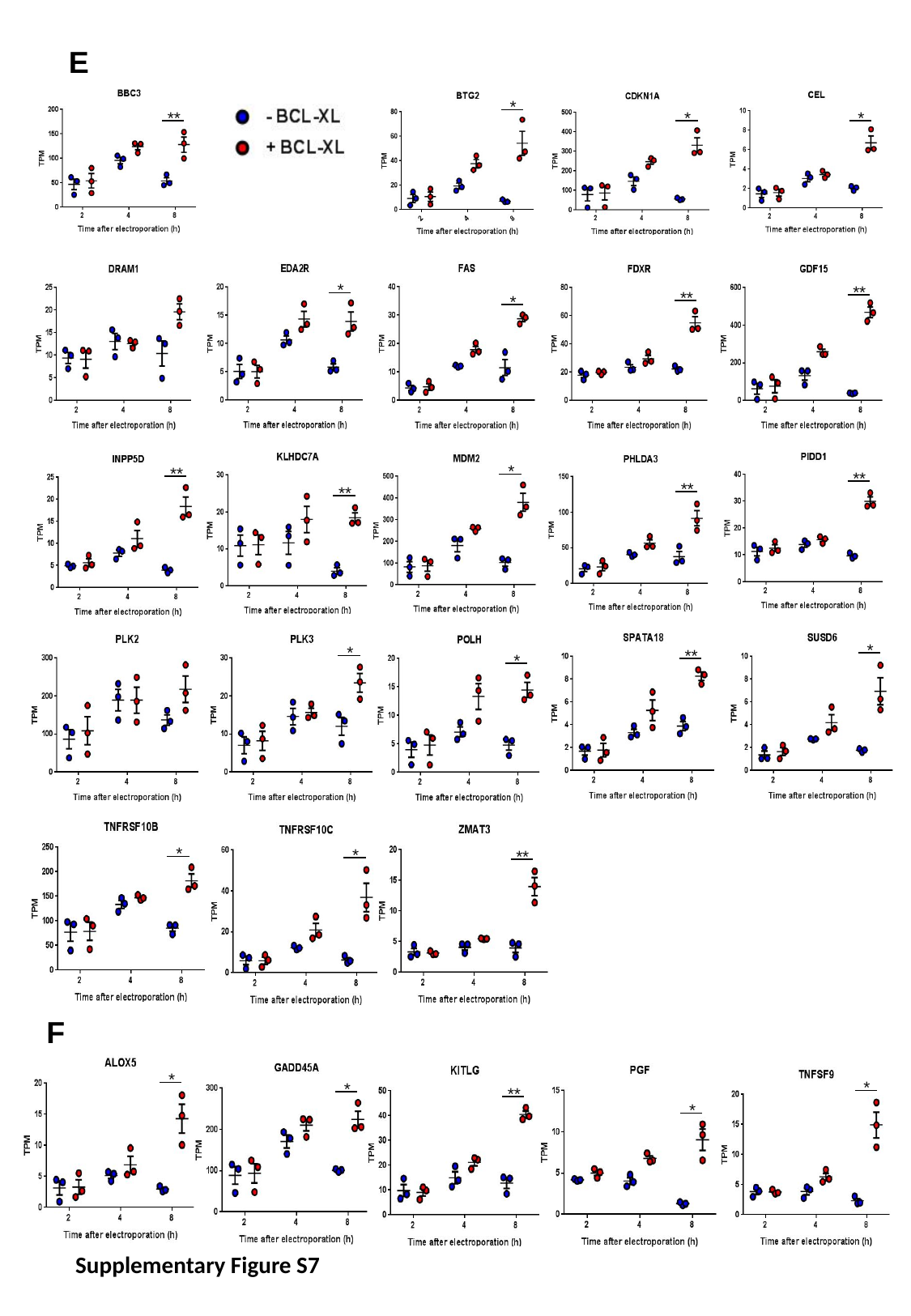

E
*
*
**
*
*
**
**
*
*
**
**
**
**
*
*
**
*
*
*
**
F
*
*
*
**
*
Supplementary Figure S7

## Slide 18
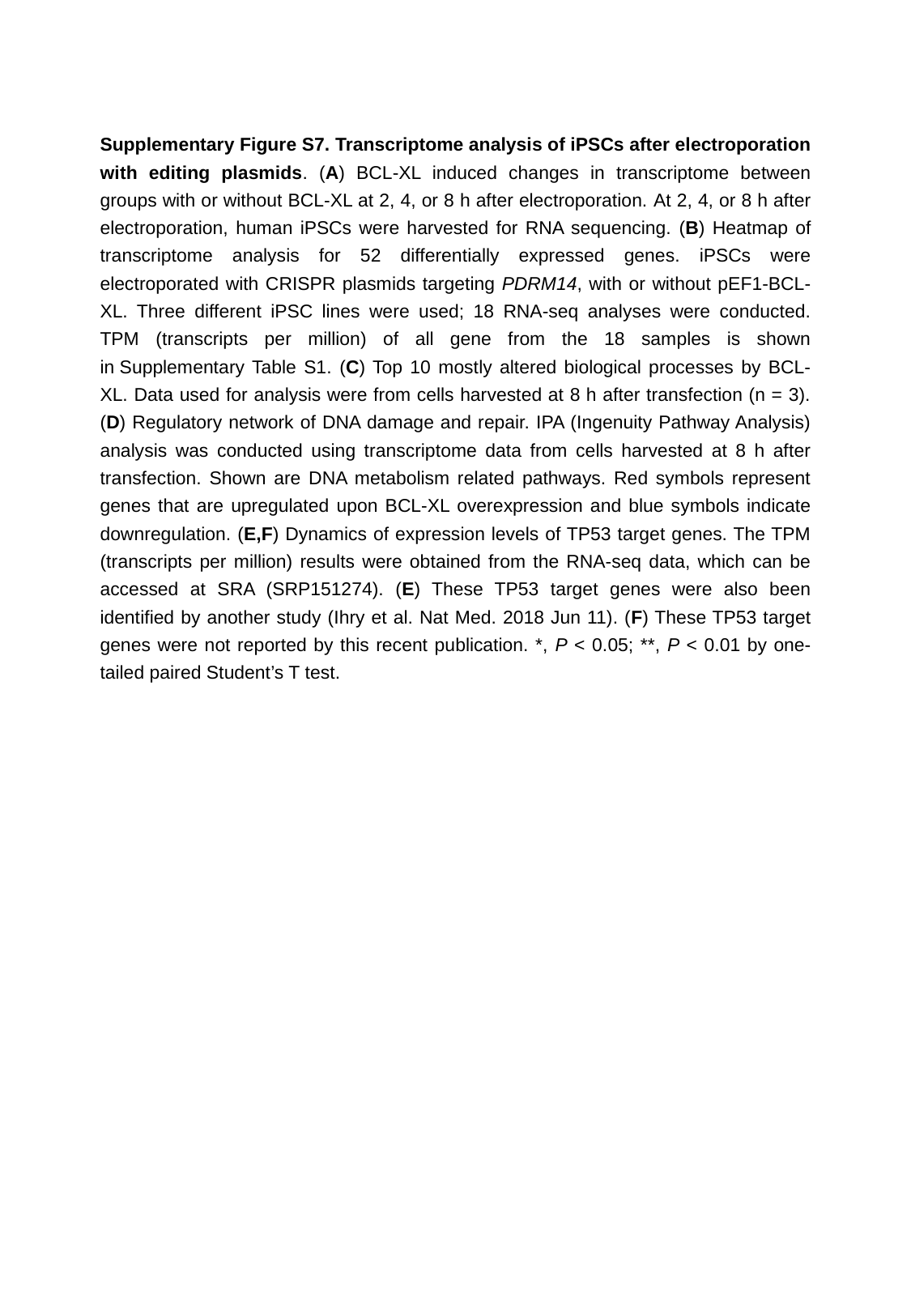

Supplementary Figure S7. Transcriptome analysis of iPSCs after electroporation with editing plasmids. (A) BCL-XL induced changes in transcriptome between groups with or without BCL-XL at 2, 4, or 8 h after electroporation. At 2, 4, or 8 h after electroporation, human iPSCs were harvested for RNA sequencing. (B) Heatmap of transcriptome analysis for 52 differentially expressed genes. iPSCs were electroporated with CRISPR plasmids targeting PDRM14, with or without pEF1-BCL-XL. Three different iPSC lines were used; 18 RNA-seq analyses were conducted. TPM (transcripts per million) of all gene from the 18 samples is shown in Supplementary Table S1. (C) Top 10 mostly altered biological processes by BCL-XL. Data used for analysis were from cells harvested at 8 h after transfection (n = 3). (D) Regulatory network of DNA damage and repair. IPA (Ingenuity Pathway Analysis) analysis was conducted using transcriptome data from cells harvested at 8 h after transfection. Shown are DNA metabolism related pathways. Red symbols represent genes that are upregulated upon BCL-XL overexpression and blue symbols indicate downregulation. (E,F) Dynamics of expression levels of TP53 target genes. The TPM (transcripts per million) results were obtained from the RNA-seq data, which can be accessed at SRA (SRP151274). (E) These TP53 target genes were also been identified by another study (Ihry et al. Nat Med. 2018 Jun 11). (F) These TP53 target genes were not reported by this recent publication. *, P < 0.05; **, P < 0.01 by one-tailed paired Student’s T test.

## Slide 19
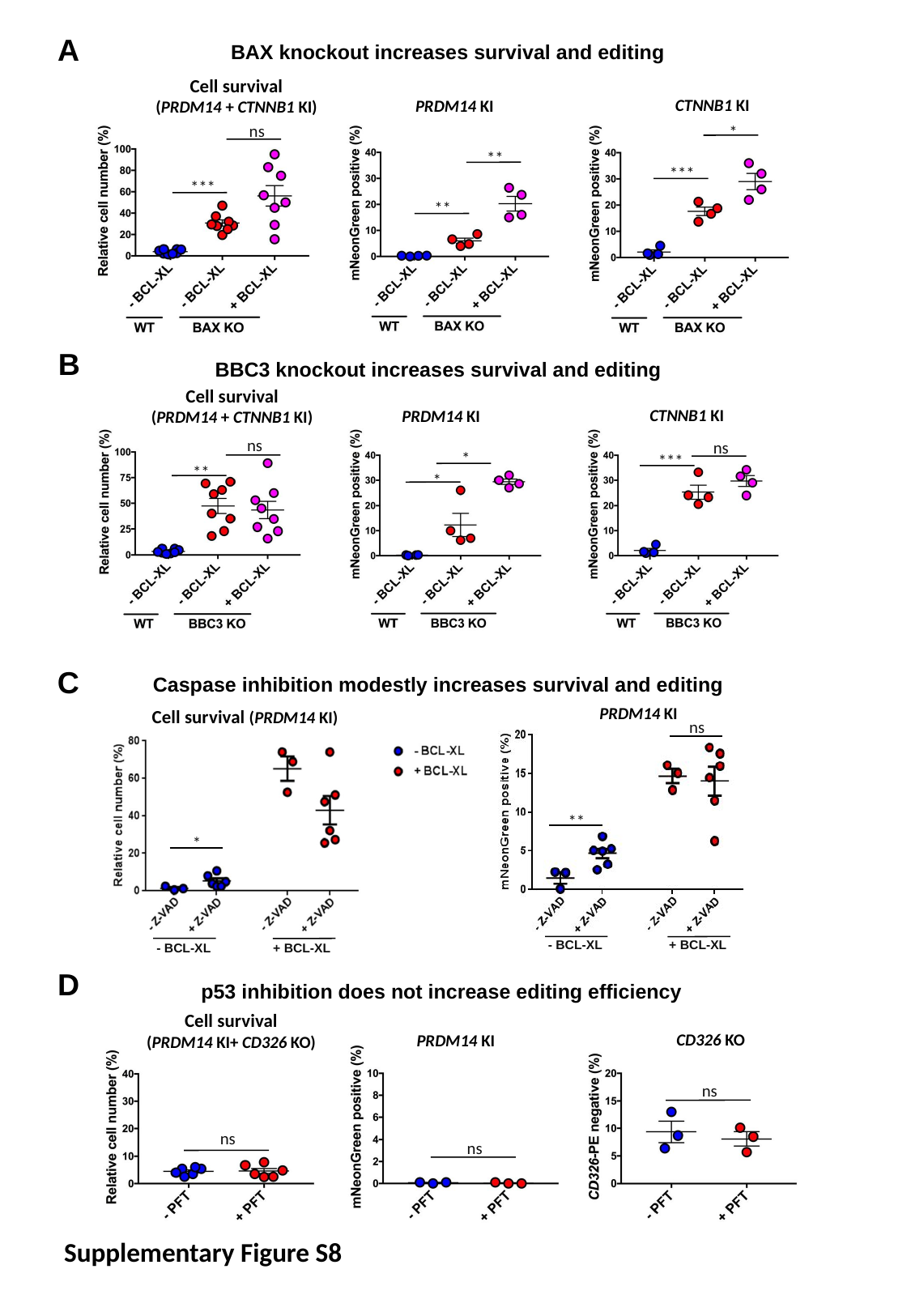

A
BAX knockout increases survival and editing
Cell survival
(PRDM14 + CTNNB1 KI)
CTNNB1 KI
PRDM14 KI
ns
*
**
***
***
**
B
BBC3 knockout increases survival and editing
Cell survival
(PRDM14 + CTNNB1 KI)
CTNNB1 KI
PRDM14 KI
ns
ns
*
***
**
*
C
Caspase inhibition modestly increases survival and editing
PRDM14 KI
Cell survival (PRDM14 KI)
ns
**
*
+ BCL-XL
- BCL-XL
- BCL-XL
+ BCL-XL
D
p53 inhibition does not increase editing efficiency
Cell survival
(PRDM14 KI+ CD326 KO)
CD326 KO
PRDM14 KI
ns
ns
ns
Supplementary Figure S8

## Slide 20
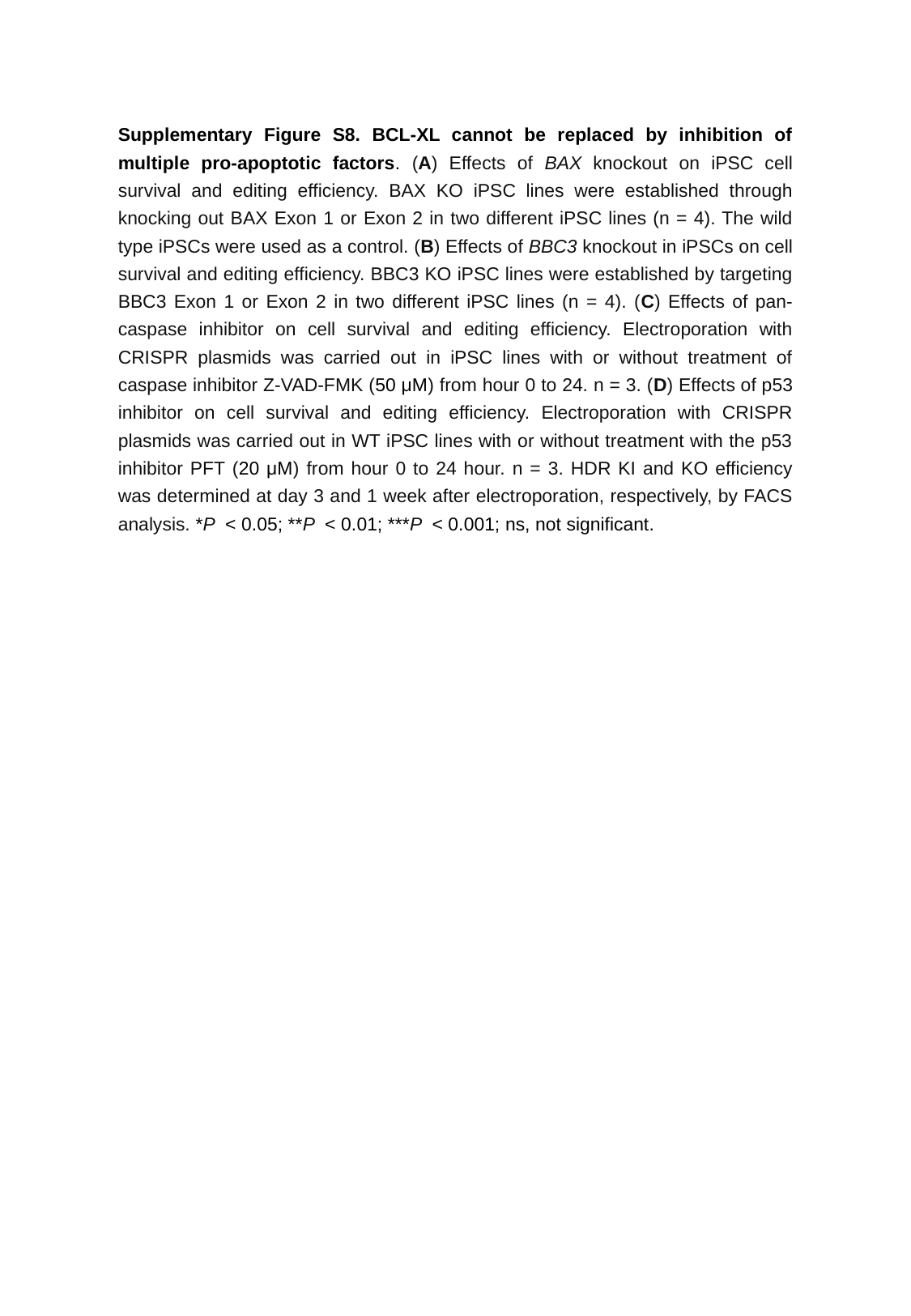

Supplementary Figure S8. BCL-XL cannot be replaced by inhibition of multiple pro-apoptotic factors. (A) Effects of BAX knockout on iPSC cell survival and editing efficiency. BAX KO iPSC lines were established through knocking out BAX Exon 1 or Exon 2 in two different iPSC lines (n = 4). The wild type iPSCs were used as a control. (B) Effects of BBC3 knockout in iPSCs on cell survival and editing efficiency. BBC3 KO iPSC lines were established by targeting BBC3 Exon 1 or Exon 2 in two different iPSC lines (n = 4). (C) Effects of pan-caspase inhibitor on cell survival and editing efficiency. Electroporation with CRISPR plasmids was carried out in iPSC lines with or without treatment of caspase inhibitor Z-VAD-FMK (50 μM) from hour 0 to 24. n = 3. (D) Effects of p53 inhibitor on cell survival and editing efficiency. Electroporation with CRISPR plasmids was carried out in WT iPSC lines with or without treatment with the p53 inhibitor PFT (20 μM) from hour 0 to 24 hour. n = 3. HDR KI and KO efficiency was determined at day 3 and 1 week after electroporation, respectively, by FACS analysis. *P < 0.05; **P < 0.01; ***P < 0.001; ns, not significant.

## Slide 21
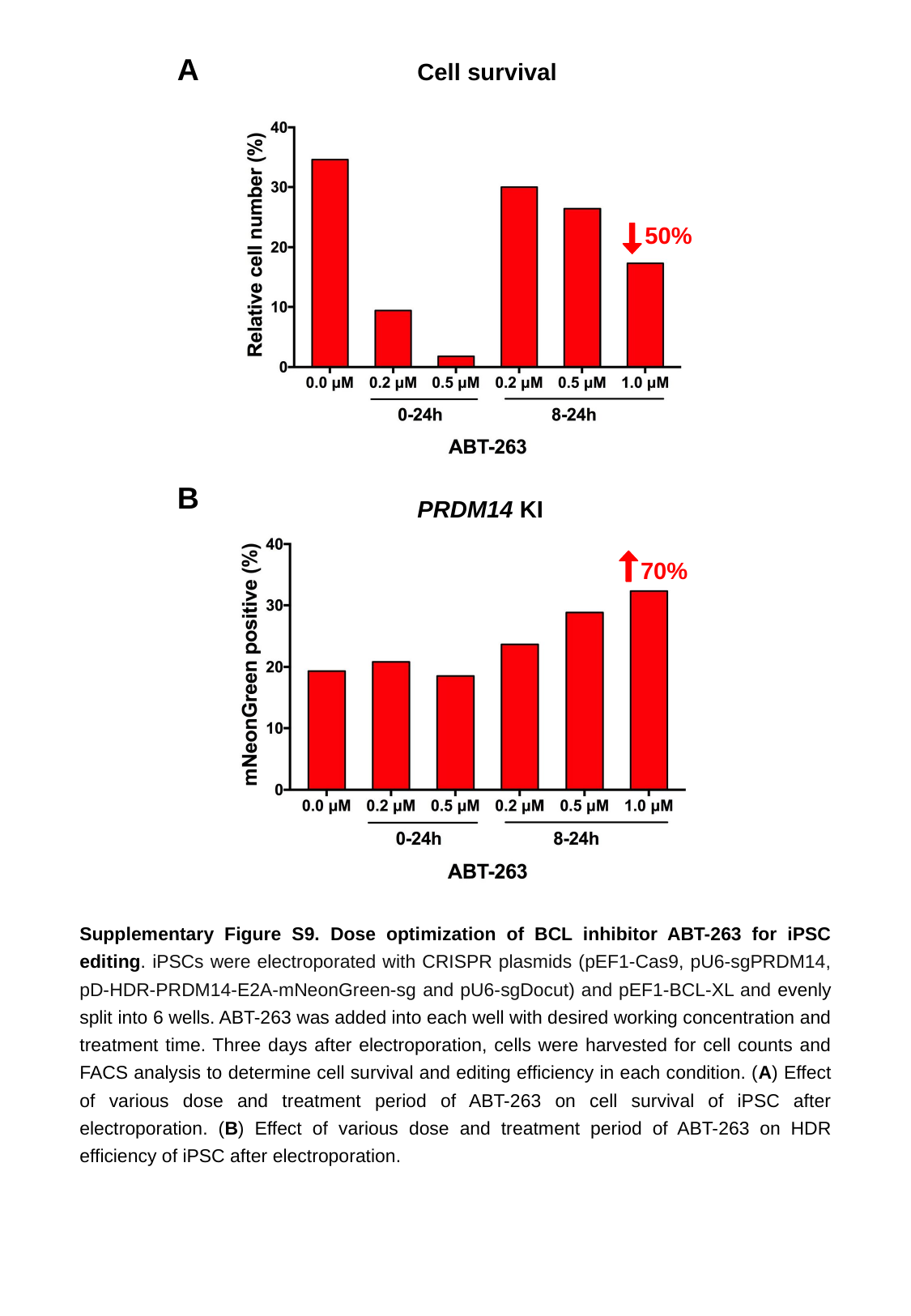

A
Cell survival
50%
B
PRDM14 KI
70%
Supplementary Figure S9. Dose optimization of BCL inhibitor ABT-263 for iPSC editing. iPSCs were electroporated with CRISPR plasmids (pEF1-Cas9, pU6-sgPRDM14, pD-HDR-PRDM14-E2A-mNeonGreen-sg and pU6-sgDocut) and pEF1-BCL-XL and evenly split into 6 wells. ABT-263 was added into each well with desired working concentration and treatment time. Three days after electroporation, cells were harvested for cell counts and FACS analysis to determine cell survival and editing efficiency in each condition. (A) Effect of various dose and treatment period of ABT-263 on cell survival of iPSC after electroporation. (B) Effect of various dose and treatment period of ABT-263 on HDR efficiency of iPSC after electroporation.

## Slide 22
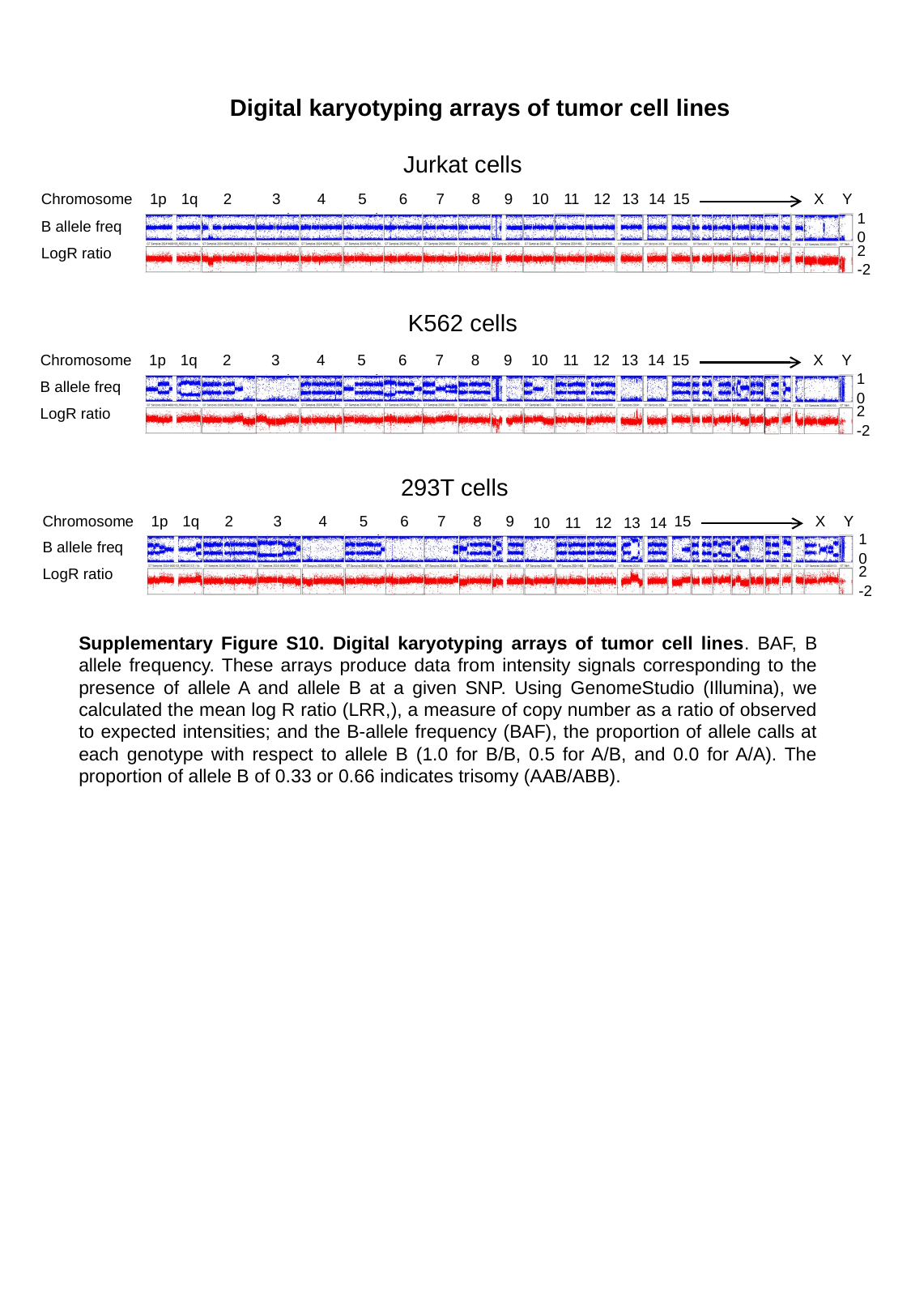

Digital karyotyping arrays of tumor cell lines
Jurkat cells
Chromosome
1p
1q
2
3
4
5
6
7
8
9
10
11
12
13
14
15
X
Y
1
B allele freq
0
2
LogR ratio
-2
K562 cells
Chromosome
1p
1q
2
3
4
5
6
7
8
9
10
11
12
13
14
15
X
Y
1
B allele freq
0
2
LogR ratio
-2
293T cells
Chromosome
1p
1q
2
3
4
5
6
7
8
9
15
X
Y
10
11
12
13
14
1
B allele freq
0
2
LogR ratio
-2
Supplementary Figure S10. Digital karyotyping arrays of tumor cell lines. BAF, B allele frequency. These arrays produce data from intensity signals corresponding to the presence of allele A and allele B at a given SNP. Using GenomeStudio (Illumina), we calculated the mean log R ratio (LRR,), a measure of copy number as a ratio of observed to expected intensities; and the B-allele frequency (BAF), the proportion of allele calls at each genotype with respect to allele B (1.0 for B/B, 0.5 for A/B, and 0.0 for A/A). The proportion of allele B of 0.33 or 0.66 indicates trisomy (AAB/ABB).

## Slide 23
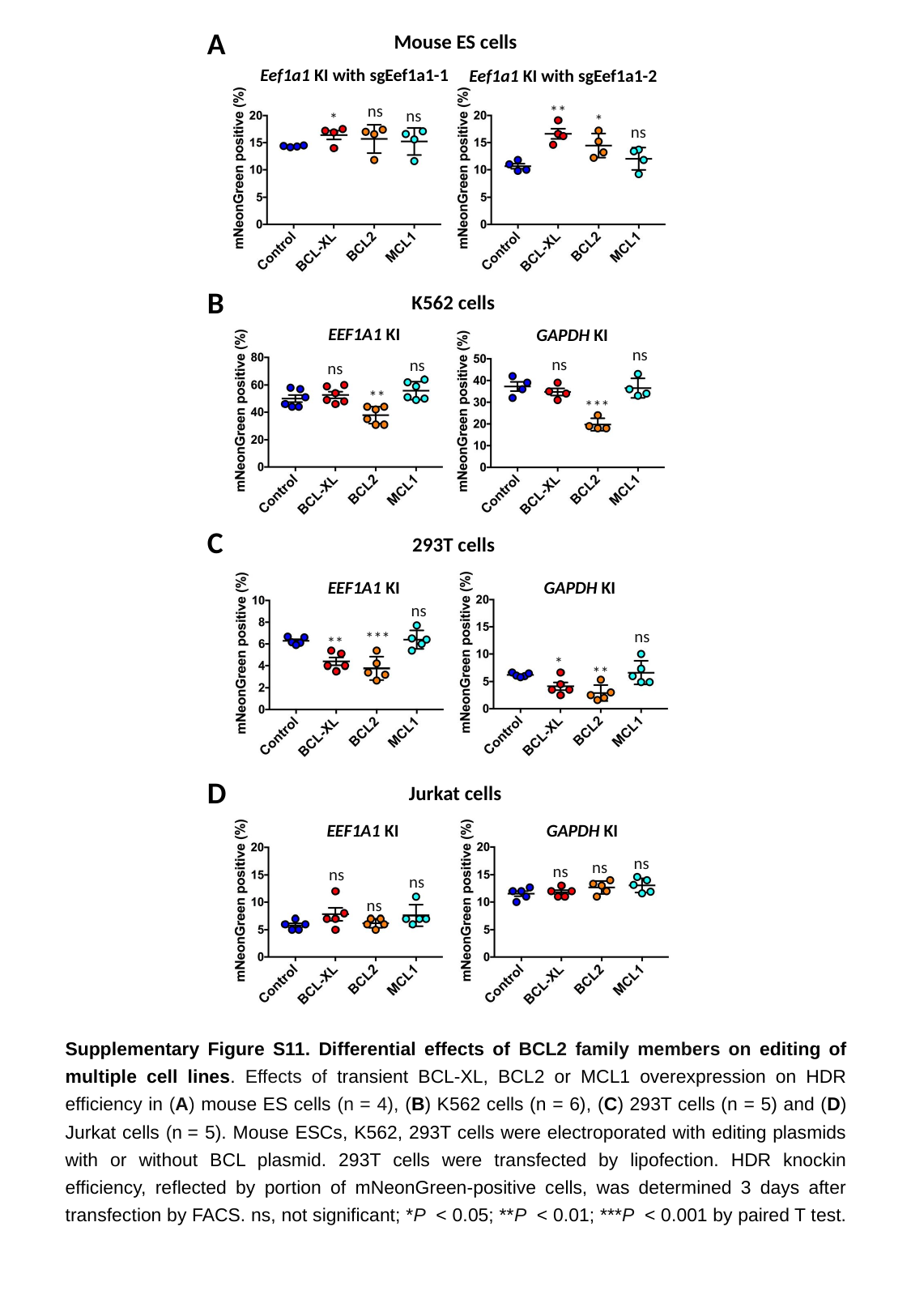

A
Mouse ES cells
Eef1a1 KI with sgEef1a1-1
Eef1a1 KI with sgEef1a1-2
ns
**
ns
*
*
ns
B
K562 cells
EEF1A1 KI
GAPDH KI
ns
ns
ns
ns
**
***
C
293T cells
GAPDH KI
EEF1A1 KI
ns
ns
***
**
*
**
D
Jurkat cells
EEF1A1 KI
GAPDH KI
ns
ns
ns
ns
ns
ns
Supplementary Figure S11. Differential effects of BCL2 family members on editing of multiple cell lines. Effects of transient BCL-XL, BCL2 or MCL1 overexpression on HDR efficiency in (A) mouse ES cells (n = 4), (B) K562 cells (n = 6), (C) 293T cells (n = 5) and (D) Jurkat cells (n = 5). Mouse ESCs, K562, 293T cells were electroporated with editing plasmids with or without BCL plasmid. 293T cells were transfected by lipofection. HDR knockin efficiency, reflected by portion of mNeonGreen-positive cells, was determined 3 days after transfection by FACS. ns, not significant; *P < 0.05; **P < 0.01; ***P < 0.001 by paired T test.

## Slide 24
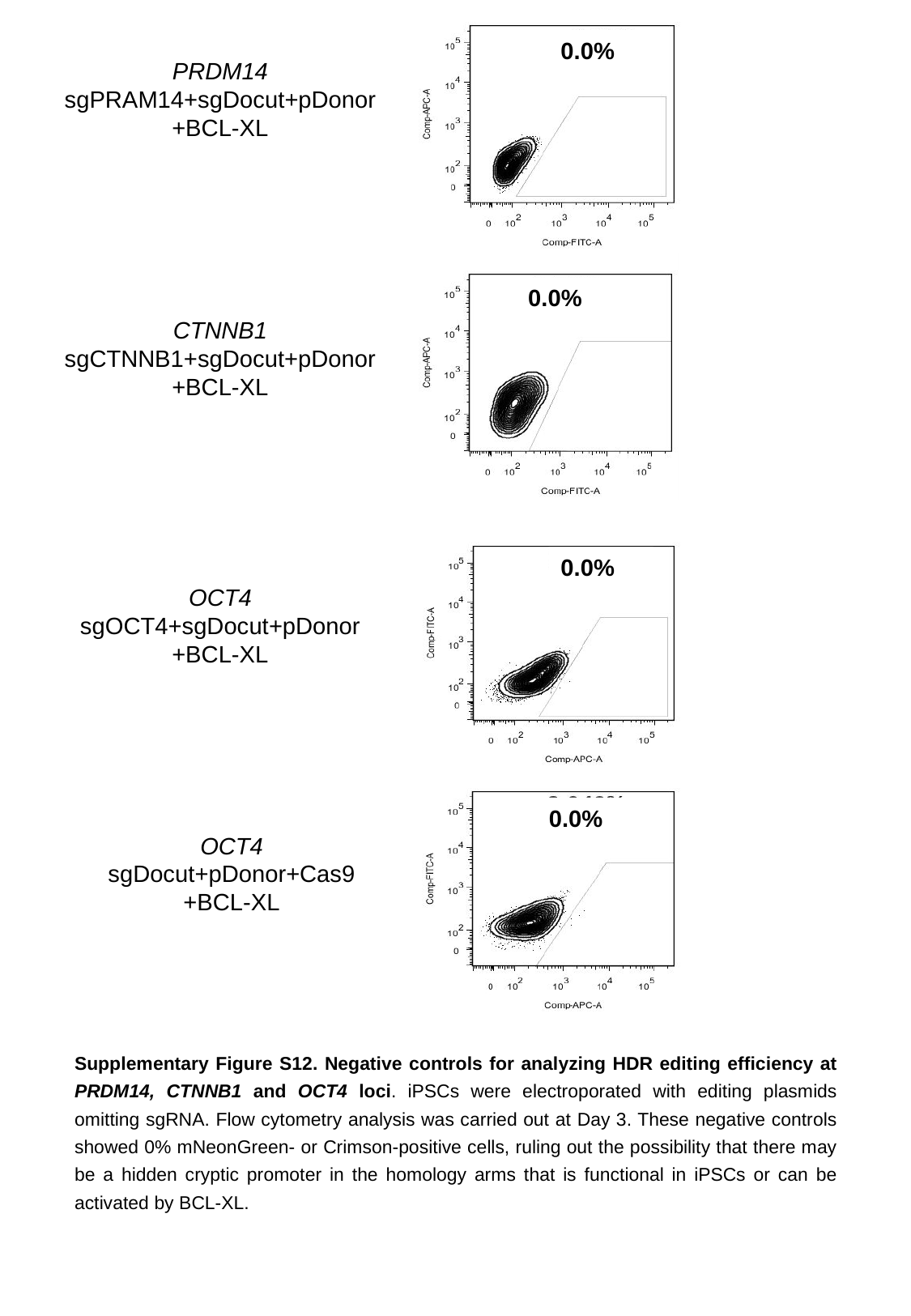

0.0%
PRDM14
sgPRAM14+sgDocut+pDonor
+BCL-XL
0.0%
CTNNB1
sgCTNNB1+sgDocut+pDonor
+BCL-XL
0.0%
OCT4
sgOCT4+sgDocut+pDonor
+BCL-XL
0.0%
OCT4
sgDocut+pDonor+Cas9
+BCL-XL
Supplementary Figure S12. Negative controls for analyzing HDR editing efficiency at PRDM14, CTNNB1 and OCT4 loci. iPSCs were electroporated with editing plasmids omitting sgRNA. Flow cytometry analysis was carried out at Day 3. These negative controls showed 0% mNeonGreen- or Crimson-positive cells, ruling out the possibility that there may be a hidden cryptic promoter in the homology arms that is functional in iPSCs or can be activated by BCL-XL.

## Slide 25
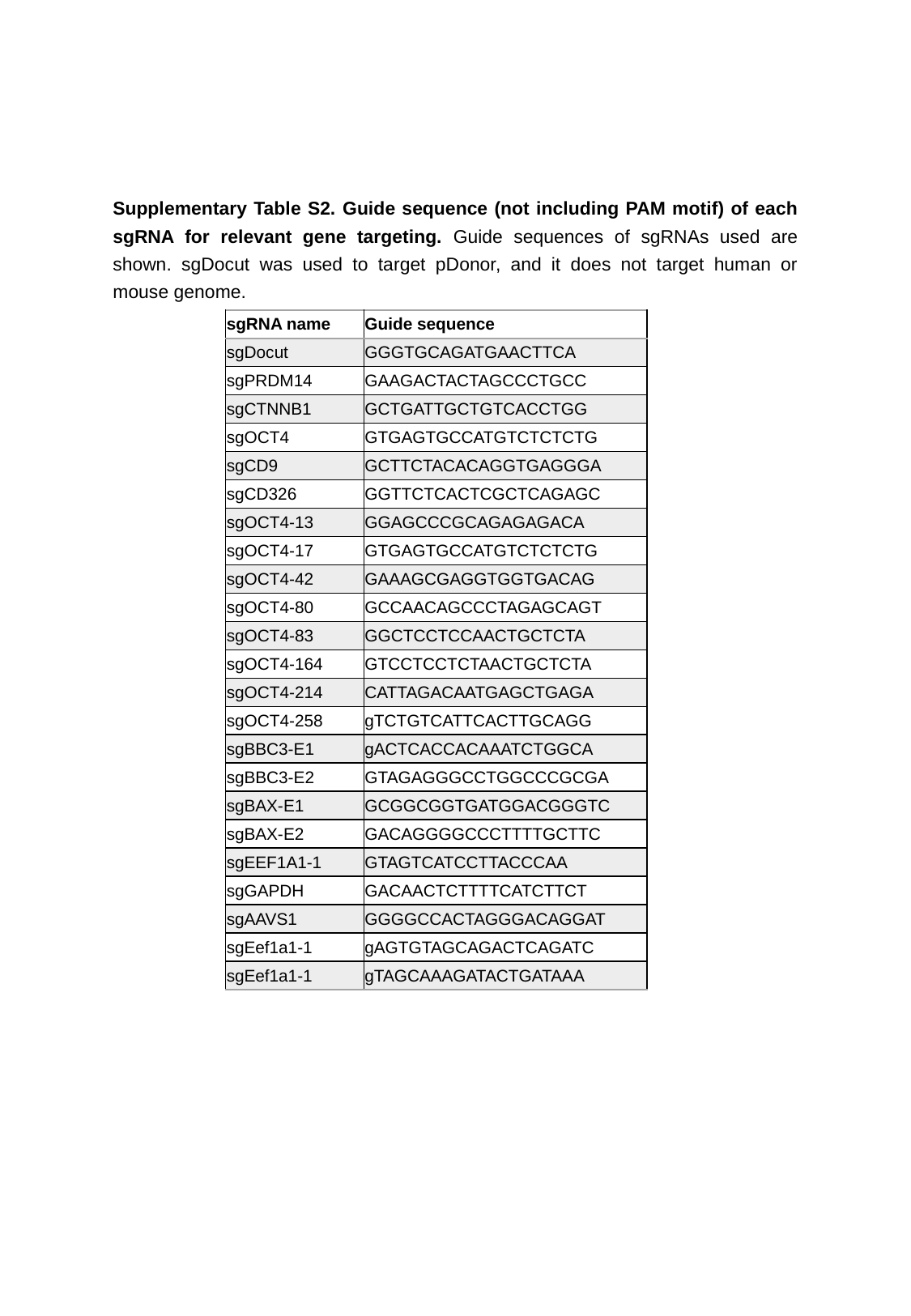

Supplementary Table S2. Guide sequence (not including PAM motif) of each sgRNA for relevant gene targeting. Guide sequences of sgRNAs used are shown. sgDocut was used to target pDonor, and it does not target human or mouse genome.
| sgRNA name | Guide sequence |
| --- | --- |
| sgDocut | GGGTGCAGATGAACTTCA |
| sgPRDM14 | GAAGACTACTAGCCCTGCC |
| sgCTNNB1 | GCTGATTGCTGTCACCTGG |
| sgOCT4 | GTGAGTGCCATGTCTCTCTG |
| sgCD9 | GCTTCTACACAGGTGAGGGA |
| sgCD326 | GGTTCTCACTCGCTCAGAGC |
| sgOCT4-13 | GGAGCCCGCAGAGAGACA |
| sgOCT4-17 | GTGAGTGCCATGTCTCTCTG |
| sgOCT4-42 | GAAAGCGAGGTGGTGACAG |
| sgOCT4-80 | GCCAACAGCCCTAGAGCAGT |
| sgOCT4-83 | GGCTCCTCCAACTGCTCTA |
| sgOCT4-164 | GTCCTCCTCTAACTGCTCTA |
| sgOCT4-214 | CATTAGACAATGAGCTGAGA |
| sgOCT4-258 | gTCTGTCATTCACTTGCAGG |
| sgBBC3-E1 | gACTCACCACAAATCTGGCA |
| sgBBC3-E2 | GTAGAGGGCCTGGCCCGCGA |
| sgBAX-E1 | GCGGCGGTGATGGACGGGTC |
| sgBAX-E2 | GACAGGGGCCCTTTTGCTTC |
| sgEEF1A1-1 | GTAGTCATCCTTACCCAA |
| sgGAPDH | GACAACTCTTTTCATCTTCT |
| sgAAVS1 | GGGGCCACTAGGGACAGGAT |
| sgEef1a1-1 | gAGTGTAGCAGACTCAGATC |
| sgEef1a1-1 | gTAGCAAAGATACTGATAAA |

## Slide 26
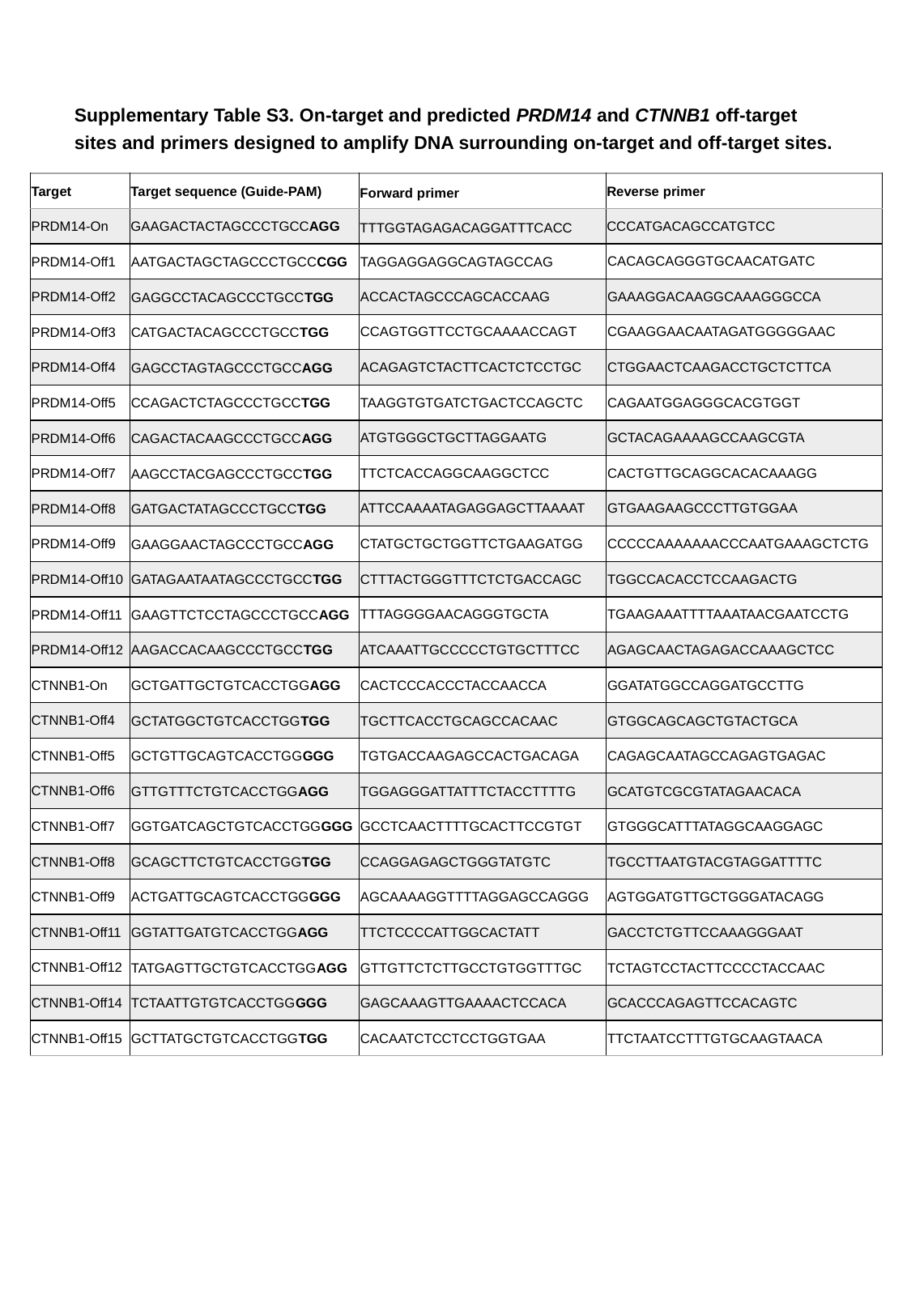

# Supplementary Table S3. On-target and predicted PRDM14 and CTNNB1 off-target sites and primers designed to amplify DNA surrounding on-target and off-target sites.
| Target | Target sequence (Guide-PAM) | Forward primer | Reverse primer |
| --- | --- | --- | --- |
| PRDM14-On | GAAGACTACTAGCCCTGCCAGG | TTTGGTAGAGACAGGATTTCACC | CCCATGACAGCCATGTCC |
| PRDM14-Off1 | AATGACTAGCTAGCCCTGCCCGG | TAGGAGGAGGCAGTAGCCAG | CACAGCAGGGTGCAACATGATC |
| PRDM14-Off2 | GAGGCCTACAGCCCTGCCTGG | ACCACTAGCCCAGCACCAAG | GAAAGGACAAGGCAAAGGGCCA |
| PRDM14-Off3 | CATGACTACAGCCCTGCCTGG | CCAGTGGTTCCTGCAAAACCAGT | CGAAGGAACAATAGATGGGGGAAC |
| PRDM14-Off4 | GAGCCTAGTAGCCCTGCCAGG | ACAGAGTCTACTTCACTCTCCTGC | CTGGAACTCAAGACCTGCTCTTCA |
| PRDM14-Off5 | CCAGACTCTAGCCCTGCCTGG | TAAGGTGTGATCTGACTCCAGCTC | CAGAATGGAGGGCACGTGGT |
| PRDM14-Off6 | CAGACTACAAGCCCTGCCAGG | ATGTGGGCTGCTTAGGAATG | GCTACAGAAAAGCCAAGCGTA |
| PRDM14-Off7 | AAGCCTACGAGCCCTGCCTGG | TTCTCACCAGGCAAGGCTCC | CACTGTTGCAGGCACACAAAGG |
| PRDM14-Off8 | GATGACTATAGCCCTGCCTGG | ATTCCAAAATAGAGGAGCTTAAAAT | GTGAAGAAGCCCTTGTGGAA |
| PRDM14-Off9 | GAAGGAACTAGCCCTGCCAGG | CTATGCTGCTGGTTCTGAAGATGG | CCCCCAAAAAAACCCAATGAAAGCTCTG |
| PRDM14-Off10 | GATAGAATAATAGCCCTGCCTGG | CTTTACTGGGTTTCTCTGACCAGC | TGGCCACACCTCCAAGACTG |
| PRDM14-Off11 | GAAGTTCTCCTAGCCCTGCCAGG | TTTAGGGGAACAGGGTGCTA | TGAAGAAATTTTAAATAACGAATCCTG |
| PRDM14-Off12 | AAGACCACAAGCCCTGCCTGG | ATCAAATTGCCCCCTGTGCTTTCC | AGAGCAACTAGAGACCAAAGCTCC |
| CTNNB1-On | GCTGATTGCTGTCACCTGGAGG | CACTCCCACCCTACCAACCA | GGATATGGCCAGGATGCCTTG |
| CTNNB1-Off4 | GCTATGGCTGTCACCTGGTGG | TGCTTCACCTGCAGCCACAAC | GTGGCAGCAGCTGTACTGCA |
| CTNNB1-Off5 | GCTGTTGCAGTCACCTGGGGG | TGTGACCAAGAGCCACTGACAGA | CAGAGCAATAGCCAGAGTGAGAC |
| CTNNB1-Off6 | GTTGTTTCTGTCACCTGGAGG | TGGAGGGATTATTTCTACCTTTTG | GCATGTCGCGTATAGAACACA |
| CTNNB1-Off7 | GGTGATCAGCTGTCACCTGGGGG | GCCTCAACTTTTGCACTTCCGTGT | GTGGGCATTTATAGGCAAGGAGC |
| CTNNB1-Off8 | GCAGCTTCTGTCACCTGGTGG | CCAGGAGAGCTGGGTATGTC | TGCCTTAATGTACGTAGGATTTTC |
| CTNNB1-Off9 | ACTGATTGCAGTCACCTGGGGG | AGCAAAAGGTTTTAGGAGCCAGGG | AGTGGATGTTGCTGGGATACAGG |
| CTNNB1-Off11 | GGTATTGATGTCACCTGGAGG | TTCTCCCCATTGGCACTATT | GACCTCTGTTCCAAAGGGAAT |
| CTNNB1-Off12 | TATGAGTTGCTGTCACCTGGAGG | GTTGTTCTCTTGCCTGTGGTTTGC | TCTAGTCCTACTTCCCCTACCAAC |
| CTNNB1-Off14 | TCTAATTGTGTCACCTGGGGG | GAGCAAAGTTGAAAACTCCACA | GCACCCAGAGTTCCACAGTC |
| CTNNB1-Off15 | GCTTATGCTGTCACCTGGTGG | CACAATCTCCTCCTGGTGAA | TTCTAATCCTTTGTGCAAGTAACA |

## Slide 27
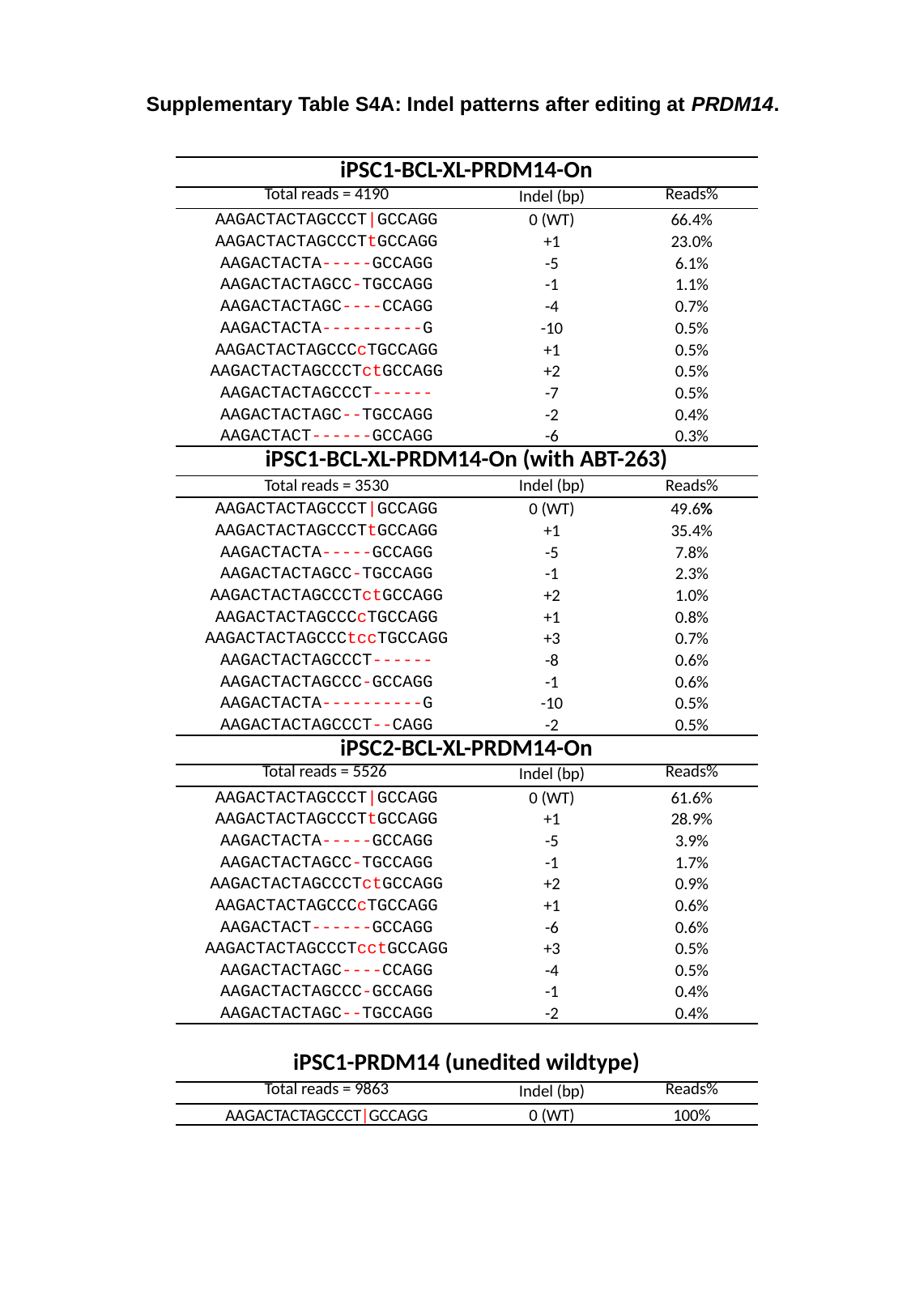

Supplementary Table S4A: Indel patterns after editing at PRDM14.
| iPSC1-BCL-XL-PRDM14-On | | |
| --- | --- | --- |
| Total reads = 4190 | Indel (bp) | Reads% |
| AAGACTACTAGCCCT|GCCAGG | 0 (WT) | 66.4% |
| AAGACTACTAGCCCTtGCCAGG | +1 | 23.0% |
| AAGACTACTA-----GCCAGG | -5 | 6.1% |
| AAGACTACTAGCC-TGCCAGG | -1 | 1.1% |
| AAGACTACTAGC----CCAGG | -4 | 0.7% |
| AAGACTACTA----------G | -10 | 0.5% |
| AAGACTACTAGCCCcTGCCAGG | +1 | 0.5% |
| AAGACTACTAGCCCTctGCCAGG | +2 | 0.5% |
| AAGACTACTAGCCCT------ | -7 | 0.5% |
| AAGACTACTAGC--TGCCAGG | -2 | 0.4% |
| AAGACTACT------GCCAGG | -6 | 0.3% |
| iPSC1-BCL-XL-PRDM14-On (with ABT-263) | | |
| Total reads = 3530 | Indel (bp) | Reads% |
| AAGACTACTAGCCCT|GCCAGG | 0 (WT) | 49.6% |
| AAGACTACTAGCCCTtGCCAGG | +1 | 35.4% |
| AAGACTACTA-----GCCAGG | -5 | 7.8% |
| AAGACTACTAGCC-TGCCAGG | -1 | 2.3% |
| AAGACTACTAGCCCTctGCCAGG | +2 | 1.0% |
| AAGACTACTAGCCCcTGCCAGG | +1 | 0.8% |
| AAGACTACTAGCCCtccTGCCAGG | +3 | 0.7% |
| AAGACTACTAGCCCT------ | -8 | 0.6% |
| AAGACTACTAGCCC-GCCAGG | -1 | 0.6% |
| AAGACTACTA----------G | -10 | 0.5% |
| AAGACTACTAGCCCT--CAGG | -2 | 0.5% |
| iPSC2-BCL-XL-PRDM14-On | | |
| Total reads = 5526 | Indel (bp) | Reads% |
| AAGACTACTAGCCCT|GCCAGG | 0 (WT) | 61.6% |
| AAGACTACTAGCCCTtGCCAGG | +1 | 28.9% |
| AAGACTACTA-----GCCAGG | -5 | 3.9% |
| AAGACTACTAGCC-TGCCAGG | -1 | 1.7% |
| AAGACTACTAGCCCTctGCCAGG | +2 | 0.9% |
| AAGACTACTAGCCCcTGCCAGG | +1 | 0.6% |
| AAGACTACT------GCCAGG | -6 | 0.6% |
| AAGACTACTAGCCCTcctGCCAGG | +3 | 0.5% |
| AAGACTACTAGC----CCAGG | -4 | 0.5% |
| AAGACTACTAGCCC-GCCAGG | -1 | 0.4% |
| AAGACTACTAGC--TGCCAGG | -2 | 0.4% |
| iPSC1-PRDM14 (unedited wildtype) | | |
| Total reads = 9863 | Indel (bp) | Reads% |
| AAGACTACTAGCCCT|GCCAGG | 0 (WT) | 100% |

## Slide 28
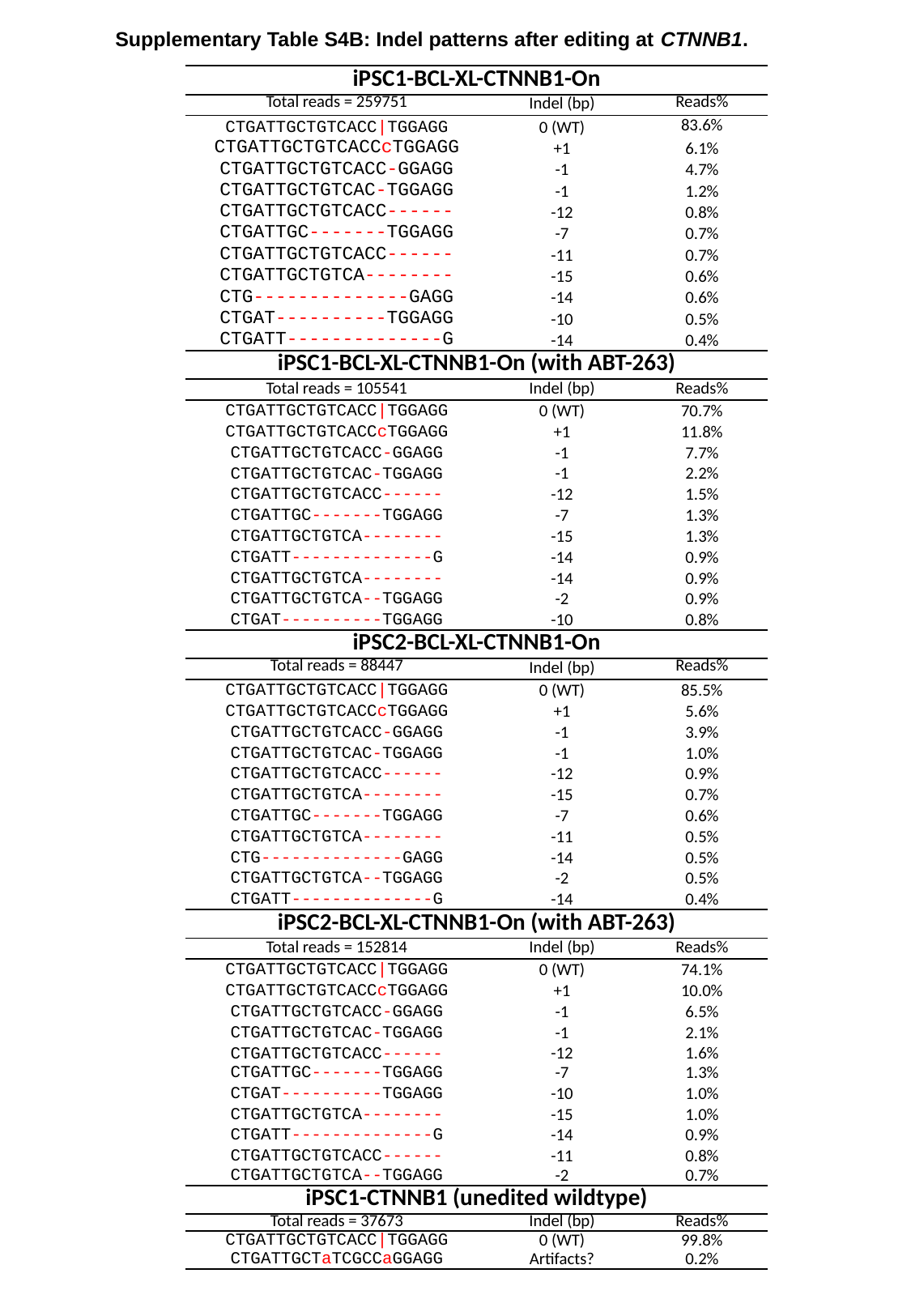

Supplementary Table S4B: Indel patterns after editing at CTNNB1.
| iPSC1-BCL-XL-CTNNB1-On | | |
| --- | --- | --- |
| Total reads = 259751 | Indel (bp) | Reads% |
| CTGATTGCTGTCACC|TGGAGG | 0 (WT) | 83.6% |
| CTGATTGCTGTCACCcTGGAGG | +1 | 6.1% |
| CTGATTGCTGTCACC-GGAGG | -1 | 4.7% |
| CTGATTGCTGTCAC-TGGAGG | -1 | 1.2% |
| CTGATTGCTGTCACC------ | -12 | 0.8% |
| CTGATTGC-------TGGAGG | -7 | 0.7% |
| CTGATTGCTGTCACC------ | -11 | 0.7% |
| CTGATTGCTGTCA-------- | -15 | 0.6% |
| CTG--------------GAGG | -14 | 0.6% |
| CTGAT----------TGGAGG | -10 | 0.5% |
| CTGATT--------------G | -14 | 0.4% |
| iPSC1-BCL-XL-CTNNB1-On (with ABT-263) | | |
| Total reads = 105541 | Indel (bp) | Reads% |
| CTGATTGCTGTCACC|TGGAGG | 0 (WT) | 70.7% |
| CTGATTGCTGTCACCcTGGAGG | +1 | 11.8% |
| CTGATTGCTGTCACC-GGAGG | -1 | 7.7% |
| CTGATTGCTGTCAC-TGGAGG | -1 | 2.2% |
| CTGATTGCTGTCACC------ | -12 | 1.5% |
| CTGATTGC-------TGGAGG | -7 | 1.3% |
| CTGATTGCTGTCA-------- | -15 | 1.3% |
| CTGATT--------------G | -14 | 0.9% |
| CTGATTGCTGTCA-------- | -14 | 0.9% |
| CTGATTGCTGTCA--TGGAGG | -2 | 0.9% |
| CTGAT----------TGGAGG | -10 | 0.8% |
| iPSC2-BCL-XL-CTNNB1-On | | |
| Total reads = 88447 | Indel (bp) | Reads% |
| CTGATTGCTGTCACC|TGGAGG | 0 (WT) | 85.5% |
| CTGATTGCTGTCACCcTGGAGG | +1 | 5.6% |
| CTGATTGCTGTCACC-GGAGG | -1 | 3.9% |
| CTGATTGCTGTCAC-TGGAGG | -1 | 1.0% |
| CTGATTGCTGTCACC------ | -12 | 0.9% |
| CTGATTGCTGTCA-------- | -15 | 0.7% |
| CTGATTGC-------TGGAGG | -7 | 0.6% |
| CTGATTGCTGTCA-------- | -11 | 0.5% |
| CTG--------------GAGG | -14 | 0.5% |
| CTGATTGCTGTCA--TGGAGG | -2 | 0.5% |
| CTGATT--------------G | -14 | 0.4% |
| iPSC2-BCL-XL-CTNNB1-On (with ABT-263) | | |
| Total reads = 152814 | Indel (bp) | Reads% |
| CTGATTGCTGTCACC|TGGAGG | 0 (WT) | 74.1% |
| CTGATTGCTGTCACCcTGGAGG | +1 | 10.0% |
| CTGATTGCTGTCACC-GGAGG | -1 | 6.5% |
| CTGATTGCTGTCAC-TGGAGG | -1 | 2.1% |
| CTGATTGCTGTCACC------ | -12 | 1.6% |
| CTGATTGC-------TGGAGG | -7 | 1.3% |
| CTGAT----------TGGAGG | -10 | 1.0% |
| CTGATTGCTGTCA-------- | -15 | 1.0% |
| CTGATT--------------G | -14 | 0.9% |
| CTGATTGCTGTCACC------ | -11 | 0.8% |
| CTGATTGCTGTCA--TGGAGG | -2 | 0.7% |
| iPSC1-CTNNB1 (unedited wildtype) | | |
| Total reads = 37673 | Indel (bp) | Reads% |
| CTGATTGCTGTCACC|TGGAGG | 0 (WT) | 99.8% |
| CTGATTGCTaTCGCCaGGAGG | Artifacts? | 0.2% |

## Slide 29
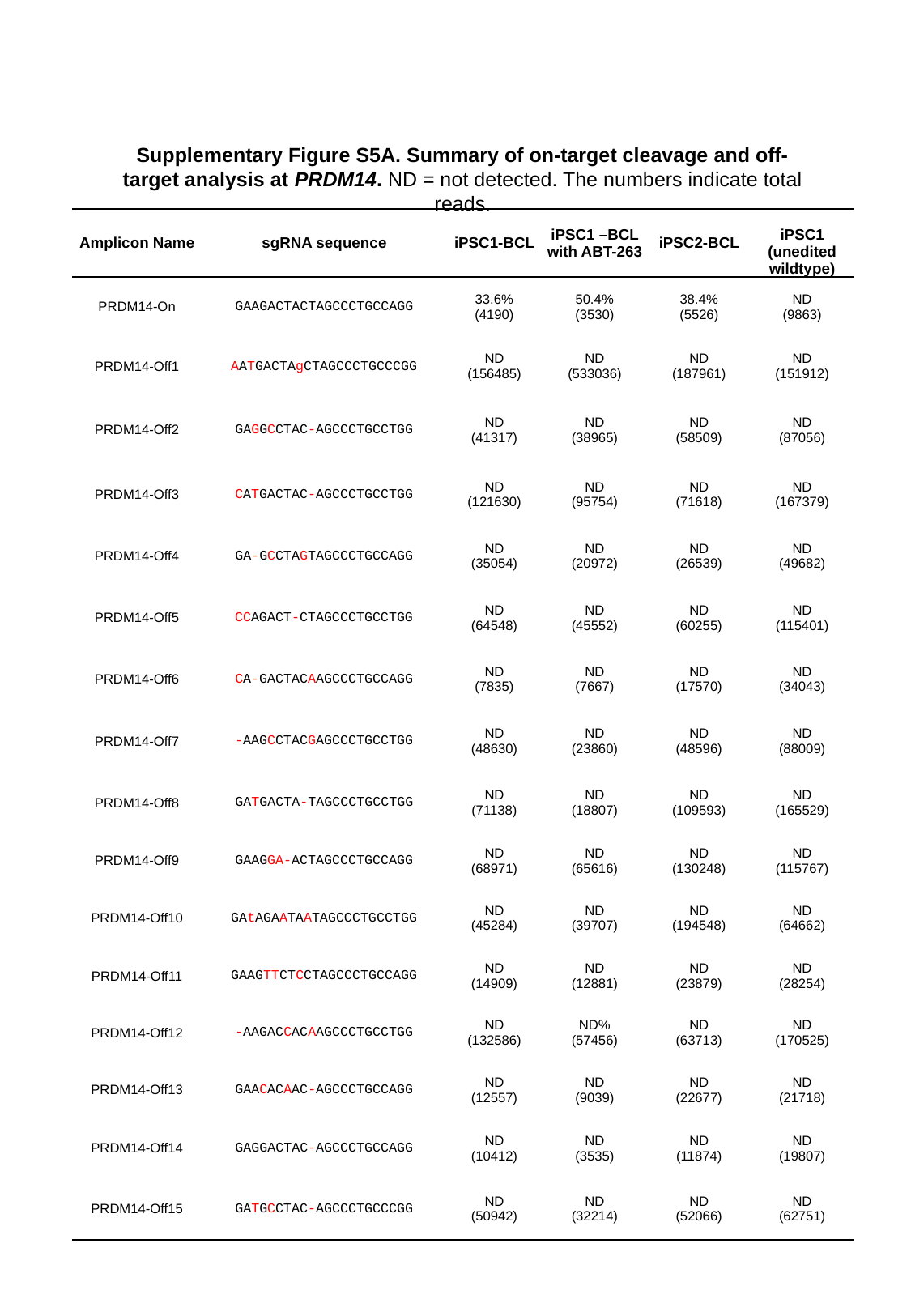

Supplementary Figure S5A. Summary of on-target cleavage and off-target analysis at PRDM14. ND = not detected. The numbers indicate total reads.
| Amplicon Name | sgRNA sequence | iPSC1-BCL | iPSC1 –BCL with ABT-263 | iPSC2-BCL | iPSC1 (unedited wildtype) |
| --- | --- | --- | --- | --- | --- |
| PRDM14-On | GAAGACTACTAGCCCTGCCAGG | 33.6% (4190) | 50.4% (3530) | 38.4% (5526) | ND (9863) |
| PRDM14-Off1 | AATGACTAgCTAGCCCTGCCCGG | ND (156485) | ND (533036) | ND (187961) | ND (151912) |
| PRDM14-Off2 | GAGGCCTAC-AGCCCTGCCTGG | ND (41317) | ND (38965) | ND (58509) | ND (87056) |
| PRDM14-Off3 | CATGACTAC-AGCCCTGCCTGG | ND (121630) | ND (95754) | ND (71618) | ND (167379) |
| PRDM14-Off4 | GA-GCCTAGTAGCCCTGCCAGG | ND (35054) | ND (20972) | ND (26539) | ND (49682) |
| PRDM14-Off5 | CCAGACT-CTAGCCCTGCCTGG | ND (64548) | ND (45552) | ND (60255) | ND (115401) |
| PRDM14-Off6 | CA-GACTACAAGCCCTGCCAGG | ND (7835) | ND (7667) | ND (17570) | ND (34043) |
| PRDM14-Off7 | -AAGCCTACGAGCCCTGCCTGG | ND (48630) | ND (23860) | ND (48596) | ND (88009) |
| PRDM14-Off8 | GATGACTA-TAGCCCTGCCTGG | ND (71138) | ND (18807) | ND (109593) | ND (165529) |
| PRDM14-Off9 | GAAGGA-ACTAGCCCTGCCAGG | ND (68971) | ND (65616) | ND (130248) | ND (115767) |
| PRDM14-Off10 | GAtAGAATAATAGCCCTGCCTGG | ND (45284) | ND (39707) | ND (194548) | ND (64662) |
| PRDM14-Off11 | GAAGTTCTCCTAGCCCTGCCAGG | ND (14909) | ND (12881) | ND (23879) | ND (28254) |
| PRDM14-Off12 | -AAGACCACAAGCCCTGCCTGG | ND (132586) | ND% (57456) | ND (63713) | ND (170525) |
| PRDM14-Off13 | GAACACAAC-AGCCCTGCCAGG | ND (12557) | ND (9039) | ND (22677) | ND (21718) |
| PRDM14-Off14 | GAGGACTAC-AGCCCTGCCAGG | ND (10412) | ND (3535) | ND (11874) | ND (19807) |
| PRDM14-Off15 | GATGCCTAC-AGCCCTGCCCGG | ND (50942) | ND (32214) | ND (52066) | ND (62751) |

## Slide 30
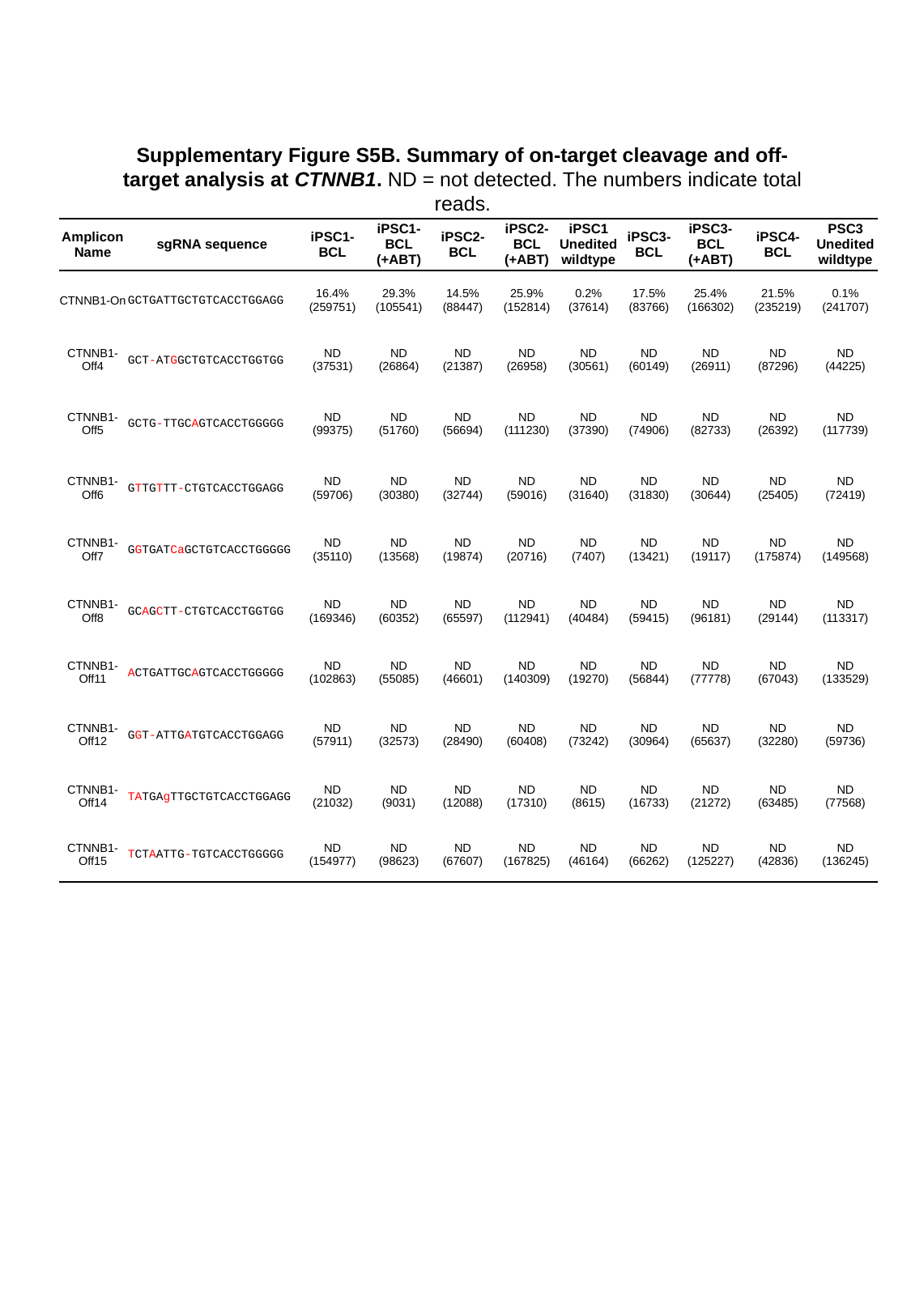

Supplementary Figure S5B. Summary of on-target cleavage and off-target analysis at CTNNB1. ND = not detected. The numbers indicate total reads.
| Amplicon Name | sgRNA sequence | iPSC1-BCL | iPSC1-BCL (+ABT) | iPSC2-BCL | iPSC2-BCL (+ABT) | iPSC1 Unedited wildtype | iPSC3-BCL | iPSC3-BCL (+ABT) | iPSC4-BCL | PSC3 Unedited wildtype |
| --- | --- | --- | --- | --- | --- | --- | --- | --- | --- | --- |
| CTNNB1-On | GCTGATTGCTGTCACCTGGAGG | 16.4% (259751) | 29.3% (105541) | 14.5% (88447) | 25.9% (152814) | 0.2% (37614) | 17.5% (83766) | 25.4% (166302) | 21.5% (235219) | 0.1% (241707) |
| CTNNB1-Off4 | GCT-ATGGCTGTCACCTGGTGG | ND (37531) | ND (26864) | ND (21387) | ND (26958) | ND (30561) | ND (60149) | ND (26911) | ND (87296) | ND (44225) |
| CTNNB1-Off5 | GCTG-TTGCAGTCACCTGGGGG | ND (99375) | ND (51760) | ND (56694) | ND (111230) | ND (37390) | ND (74906) | ND (82733) | ND (26392) | ND (117739) |
| CTNNB1-Off6 | GTTGTTT-CTGTCACCTGGAGG | ND (59706) | ND (30380) | ND (32744) | ND (59016) | ND (31640) | ND (31830) | ND (30644) | ND (25405) | ND (72419) |
| CTNNB1-Off7 | GGTGATCaGCTGTCACCTGGGGG | ND (35110) | ND (13568) | ND (19874) | ND (20716) | ND (7407) | ND (13421) | ND (19117) | ND (175874) | ND (149568) |
| CTNNB1-Off8 | GCAGCTT-CTGTCACCTGGTGG | ND (169346) | ND (60352) | ND (65597) | ND (112941) | ND (40484) | ND (59415) | ND (96181) | ND (29144) | ND (113317) |
| CTNNB1-Off11 | ACTGATTGCAGTCACCTGGGGG | ND (102863) | ND (55085) | ND (46601) | ND (140309) | ND (19270) | ND (56844) | ND (77778) | ND (67043) | ND (133529) |
| CTNNB1-Off12 | GGT-ATTGATGTCACCTGGAGG | ND (57911) | ND (32573) | ND (28490) | ND (60408) | ND (73242) | ND (30964) | ND (65637) | ND (32280) | ND (59736) |
| CTNNB1-Off14 | TATGAgTTGCTGTCACCTGGAGG | ND (21032) | ND (9031) | ND (12088) | ND (17310) | ND (8615) | ND (16733) | ND (21272) | ND (63485) | ND (77568) |
| CTNNB1-Off15 | TCTAATTG-TGTCACCTGGGGG | ND (154977) | ND (98623) | ND (67607) | ND (167825) | ND (46164) | ND (66262) | ND (125227) | ND (42836) | ND (136245) |

## Slide 31
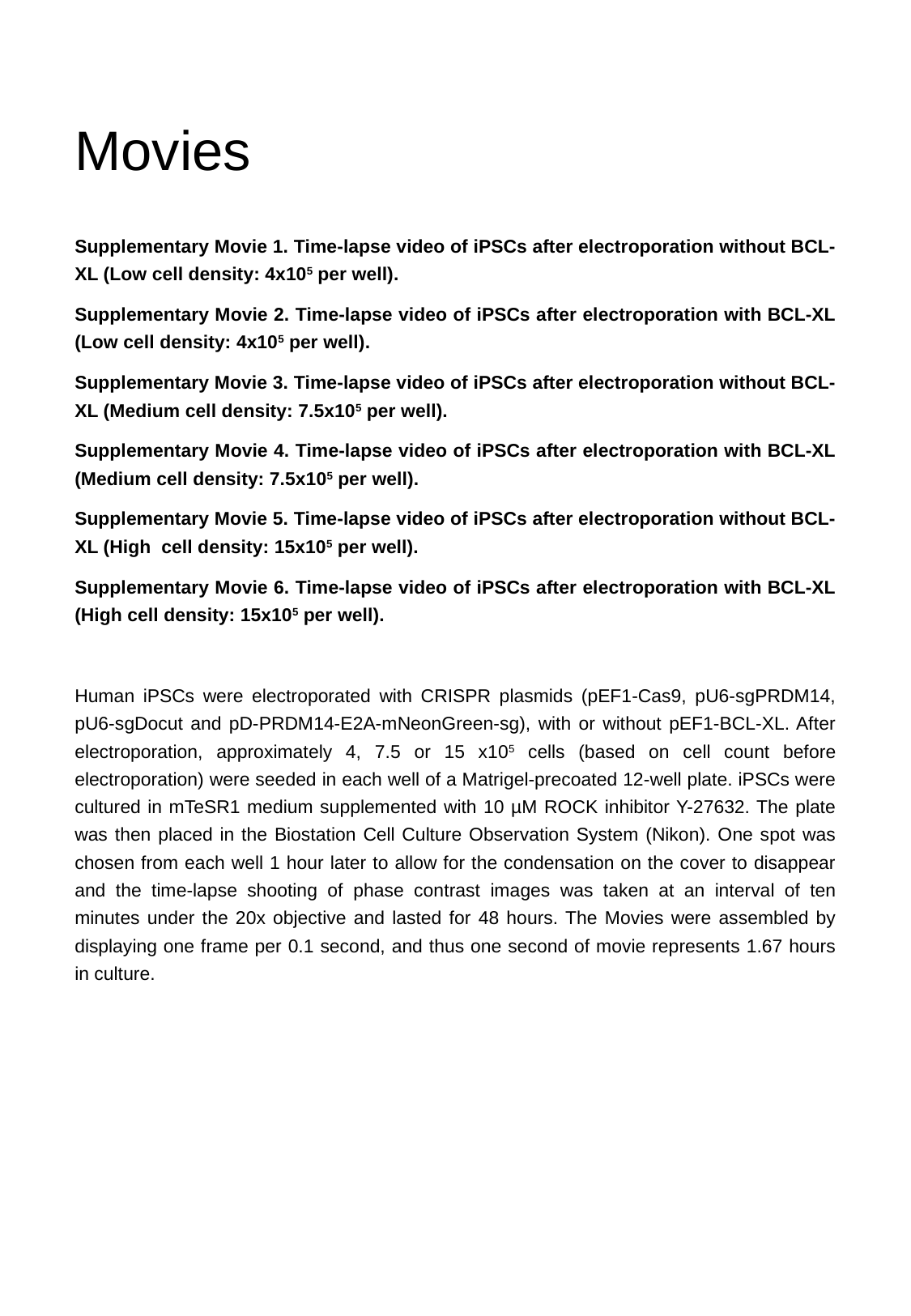

# Movies
Supplementary Movie 1. Time-lapse video of iPSCs after electroporation without BCL-XL (Low cell density: 4x10^5^ per well).
Supplementary Movie 2. Time-lapse video of iPSCs after electroporation with BCL-XL (Low cell density: 4x10^5^ per well).
Supplementary Movie 3. Time-lapse video of iPSCs after electroporation without BCL-XL (Medium cell density: 7.5x10^5^ per well).
Supplementary Movie 4. Time-lapse video of iPSCs after electroporation with BCL-XL (Medium cell density: 7.5x10^5^ per well).
Supplementary Movie 5. Time-lapse video of iPSCs after electroporation without BCL-XL (High cell density: 15x105 per well).
Supplementary Movie 6. Time-lapse video of iPSCs after electroporation with BCL-XL (High cell density: 15x105 per well).
Human iPSCs were electroporated with CRISPR plasmids (pEF1-Cas9, pU6-sgPRDM14, pU6-sgDocut and pD-PRDM14-E2A-mNeonGreen-sg), with or without pEF1-BCL-XL. After electroporation, approximately 4, 7.5 or 15 x105 cells (based on cell count before electroporation) were seeded in each well of a Matrigel-precoated 12-well plate. iPSCs were cultured in mTeSR1 medium supplemented with 10 µM ROCK inhibitor Y-27632. The plate was then placed in the Biostation Cell Culture Observation System (Nikon). One spot was chosen from each well 1 hour later to allow for the condensation on the cover to disappear and the time-lapse shooting of phase contrast images was taken at an interval of ten minutes under the 20x objective and lasted for 48 hours. The Movies were assembled by displaying one frame per 0.1 second, and thus one second of movie represents 1.67 hours in culture.
